# Impact of a TLR9 agonist and broadly neutralizing antibodies on HIV-1 persistence: the randomized phase 2a TITAN trial

**DOI:** 10.1038/s41591-023-02547-6

**Published:** 2023-09-11

**Authors:** Jesper D. Gunst, Jesper F. Højen, Marie H. Pahus, Miriam Rosás-Umbert, Birgitte Stiksrud, James H. McMahon, Paul W. Denton, Henrik Nielsen, Isik S. Johansen, Thomas Benfield, Steffen Leth, Jan Gerstoft, Lars Østergaard, Mariane H. Schleimann, Rikke Olesen, Henrik Støvring, Line Vibholm, Nina Weis, Anne M. Dyrhol-Riise, Karen B. H. Pedersen, Jillian S. Y. Lau, Dennis C. Copertino, Noemi Linden, Tan T. Huynh, Victor Ramos, R. Brad Jones, Sharon R. Lewin, Martin Tolstrup, Thomas A. Rasmussen, Michel C. Nussenzweig, Marina Caskey, Dag Henrik Reikvam, Ole S. Søgaard

**Affiliations:** 1 Department of Clinical Medicine, Aarhus University, Aarhus, Denmark; 2 Department of Infectious Diseases, Aarhus University Hospital, Aarhus, Denmark; 3 Department of Infectious Diseases, Oslo University Hospital, Oslo, Norway; 4 Department of Infectious Diseases, Alfred Hospital, Melbourne, VIC Australia; 5 Department of Biology, University of Nebraska at Omaha, Omaha, NE USA; 6 Department of Infectious Diseases, Aalborg University Hospital, Aalborg, Denmark; 7 Department of Clinical Medicine, Aalborg University, Aalborg, Denmark; 8 Department of Infectious Diseases, Odense University Hospital, University of Southern Denmark, Odense, Denmark; 9 Department of Infectious Diseases, Copenhagen University Hospital - Amager and Hvidovre, Hvidovre, Denmark; 10 Department of Clinical Medicine, University of Copenhagen, Copenhagen, Denmark; 11 Department of Internal Medicine, Gødstrup Hospital, Gødstrup, Denmark; 12 Viro-Immunology Research Unit, Department of Infectious Diseases, Rigshospitalet, Copenhagen, Denmark; 13 Department of Public Health, Clinical Pharmacology, Pharmacy and Environmental Medicine, University of Southern Denmark, Odense, Denmark; 14 Institute of Clinical Medicine, University of Oslo, Oslo, Norway; 15 Department of Infectious Diseases, University of Melbourne at the Peter Doherty Institute for Infection and Immunity, Melbourne, VIC Australia; 16 Victorian Infectious Diseases Service, Royal Melbourne Hospital at the Peter Doherty Institute for Infection and Immunity, Melbourne, VIC Australia; 17 Infectious Diseases Division, Department of Medicine, Weill Cornell Medical College, New York, NY USA; 18 Department of Microbiology and Immunology, Weill Cornell Graduate School of Medical Sciences, New York, NY USA; 19 Laboratory of Molecular Immunology, The Rockefeller University, New York, NY USA; 20 Howard Hughes Medical Institute, The Rockefeller University, New York, NY USA

**Keywords:** Randomized controlled trials, HIV infections

## Abstract

Inducing antiretroviral therapy (ART)-free virological control is a critical step toward a human immunodeficiency virus type 1 (HIV-1) cure. In this phase 2a, placebo-controlled, double-blinded trial, 43 people (85% males) with HIV-1 on ART were randomized to (1) placebo/placebo, (2) lefitolimod (TLR9 agonist)/placebo, (3) placebo/broadly neutralizing anti-HIV-1 antibodies (bNAbs) or (4) lefitolimod/bNAb. ART interruption (ATI) started at week 3. Lefitolimod was administered once weekly for the first 8 weeks, and bNAbs were administered twice, 1 d before and 3 weeks after ATI. The primary endpoint was time to loss of virologic control after ATI. The median delay in time to loss of virologic control compared to the placebo/placebo group was 0.5 weeks (*P* = 0.49), 12.5 weeks (*P* = 0.003) and 9.5 weeks (*P* = 0.004) in the lefitolimod/placebo, placebo/bNAb and lefitolimod/bNAb groups, respectively. Among secondary endpoints, viral doubling time was slower for bNAb groups compared to non-bNAb groups, and the interventions were overall safe. We observed no added benefit of lefitolimod. Despite subtherapeutic plasma bNAb levels, 36% (4/11) in the placebo/bNAb group compared to 0% (0/10) in the placebo/placebo group maintained virologic control after the 25-week ATI. Although immunotherapy with lefitolimod did not lead to ART-free HIV-1 control, bNAbs may be important components in future HIV-1 curative strategies. ClinicalTrials.gov identifier: NCT03837756.

## Abstract (web summary)

In people with HIV-1 undergoing antiretroviral treatment interruption, lefitolimod combined with broadly neutralizing antibodies (bNAbs) did not delay viral rebound beyond that achieved with bNAbs alone, raising the question of how to optimize combination immunotherapy to control HIV-1.

## Main

Human immunodeficiency virus type 1 (HIV-1) infection persists mainly in CD4^+^ T cells as a long-lived HIV-1 reservoir. Continuous lifelong antiretroviral therapy (ART) is needed to suppress viral replication and prevent disease progression. When ART is stopped, viral replication quickly resumes, leading to rebound of plasma viremia within weeks in almost all individuals^1–5^.

Many HIV-1 curative strategies aim to enhance HIV-1-specific immunity as a means to achieve ART-free HIV-1 virologic control. Until recently, HIV-1 cure trials have typically involved people with HIV-1 (PWH) on long-term suppressive ART, which is continued during the therapeutic interventions^6^. In these individuals, the level of HIV-1 antigen both in blood and tissues is extremely low due to the effective modern ART regimens. The scarcity of antigen may explain why experimental immunotherapies, such as cytokines and other immunostimulatory agents, have had limited impact on HIV-1-specific immunity and viral persistence in clinical trials^7,8^.

One promising immunostimulatory agent is lefitolimod (MGN1703), a dumbbell-shaped DNA molecule that triggers Toll-like receptor (TLR) 9 signaling in human plasmacytoid dendritic cells (pDCs) and B cells^9,10^. Moreover, TLR9 agonists have been shown to enhance potent antibody-dependent cellular cytotoxicity (ADCC) in vitro^11^. In vivo, TLR9 agonist administration among PWH on long-term suppressive ART induced activation of pDCs, leading to potent upregulation of antiviral immune responses^12–15^. During TLR9 agonist treatment, the proportion of activated cytotoxic (CD56^dim^CD16^+^) natural killer (NK) and CD8^+^ T cells expanded in blood^12,13^ and lymph nodes^14^. This might facilitate killing of infected cells, as ex vivo studies have shown that priming of effector cells, such as cytotoxic T lymphocytes (CTLs) and NK cells, enhances their ability to recognize and eliminate antigen-expressing infected cells^16–20^. Finally, TLR9 agonist treatment may directly impact the HIV-1 reservoir through cytokine release or cell-to-cell-mediated activation^21^. In a small clinical trial, an increased frequency in viral blips during lefitolimod administration was observed among PWH on ART, suggesting that lefitolimod may have a latency-reversing effect on the HIV-1 reservoir^12^.

The immunological effects of lefitolimod could potentially be boosted either by administering the drug in the presence of higher antigen load or by co-administration with broadly neutralizing anti-HIV-1 antibodies (bNAbs), such as 3BNC117 and 10-1074, which recognize non-overlapping epitopes on the HIV-1 envelope protein gp120 (refs. ^22,23^). In support of the latter concept, non-human primate (NHP) studies have shown that administration of a TLR7 agonist, which, similarly to a TLR9 agonist, activates pDCs and triggers antiviral immune responses, in combination with bNAbs led to ART-free virologic control in a large proportion of animals during ART interruption (ATI)^24–26^. In phase 1–2 clinical trials, repeated administration (3–8 doses) of the combination of 3BNC117 and 10-1074 maintained viral suppression for ~13 weeks after last bNAb infusion when administered instead of ART^27–29^. Finally, owing to Fc-mediated engagement of effector and antigen-presenting cells, bNAbs independently exert immunomodulatory effects, and bNAb treatment has been shown to augment HIV-1-specific T cell immunity both in viremic NHPs and in humans^6,30–32^.

**Fig. 1:**
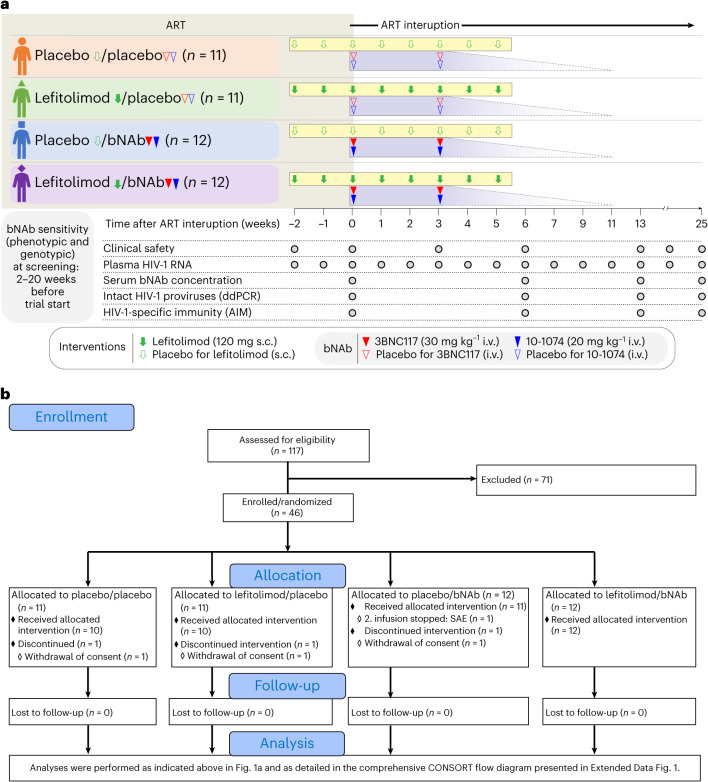
TITAN trial design (a) and abbreviated CONSORT flow diagram (b). Solid green arrows indicate lefitolimod injections. Solid red and blue triangles indicate 3BNC117 and 10-1074 infusions, respectively. Empty green arrows as well as red and blue triangles indicate placebo injections or infusions, respectively. Gray shaded areas indicate time on ART, and white shaded areas indicate still interrupting ART during the 25 weeks of ATI. The analysis section is presented in full in Extended Data Fig. 1. SAE, severe adverse event.

Collectively, these observations led us to hypothesize that lefitolimod alone or in combination with two doses of 3BNC117 and 10-1074 administered in the setting of ATI could potentially enhance elimination of infected cells and boost HIV-1-specific CD8^+^ T cell immunity, leading to ART-free virologic control (Fig. 1a)^33^. To address this hypothesis, we conducted an investigator-initiated, randomized, placebo-controlled, double-blinded phase 2a trial to determine the impact of lefitolimod and 3BNC117 + 10-1074 on virologic control among PWH undergoing ATI. Secondary endpoints were safety and viral rebound kinetics.

## Results

### Participants and follow-up

**Extended Data Fig. 1:**
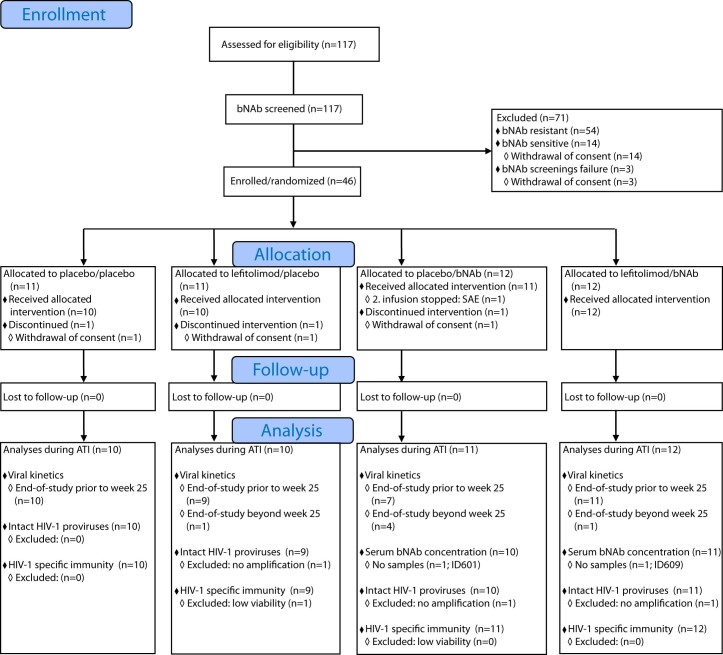
A comprehensive CONSORT Flow Diagram. ART, antiretroviral therapy; ATI, ART interruption; bNAb, broadly neutralizing antibody, SAE, severe adverse event.

Eligible individuals were recruited from 12 April 2019 to 5 November 2021, and follow-up concluded on 9 June 2022. Of 117 individuals who were screened for sensitivity to 3BNC117 and 10-1074 by phenotypic or sequence-based analysis, 46 participants were enrolled in the study, of whom three withdrew consent for personal reasons and did not receive the random allocation treatment (Fig. 1b and Extended Data Fig. 1). Of the remaining 43 participants, 10 were randomly allocated to placebo/placebo, 10 to lefitolimod/placebo, 11 to placebo/bNAb and 12 to lefitolimod/bNAb. Lefitolimod (120 mg) or placebo was dosed subcutaneously (s.c.) once weekly for 8 weeks starting 2 weeks before the ATI and ending at the beginning of week 6 of the ATI (Fig. 1a). 3BNC117 (30 mg kg^−1^) and 10-1074 (20 mg kg^−1^) or placebo were given as sequential intravenous (i.v.) infusions the day before starting the ATI and 3 weeks into the ATI. One participant in the placebo/bNAb group discontinued the ATI and re-started ART after an infusion-related reaction during the second bNAb infusion (Fig. 1b). This person was included in the analyses. No participants were lost to follow-up.

The four randomization groups were overall well balanced on key demographic and genetic parameters (Table 1 and Extended Data Table 1). Study participants were mainly white (87%) and male (85%). Median age at enrollment was 50 years (interquartile range (IQR): 41–54), and median time since HIV-1 diagnosis was 10 years (IQR: 6–15). Participants had been on ART for a median of 8 years (IQR: 5–12), and the median CD4^+^ T cell count was 801 cells per mm^3^ (range: 507–2,100) (Extended Data Table 1). Approximately three-quarters of the individuals (72%) had HIV-1 subtype B; the remaining participants had other HIV-1 subtypes (Table 1 and Extended Data Table 1). Across the four groups, human leukocyte antigen (HLA) class I alleles associated with rapid HIV-1 progression (that is, B*07 and B*35) or elite control (that is, B*27, B*57 and B*58) were represented in 43% and 11% of the participants, respectively (Extended Data Table 1).

**Table 1:** Baseline characteristics of the study population

	Placebo/placebo (*n* = 11)	Lefitolimod/placebo (*n* = 11)	Placebo/bNAb (*n* = 12)	Lefitolimod/bNAb (*n* = 12)
Age (years)	54 (45–60)	44 (38–53)	51 (41–54)	48 (31–53)
Female sex	2 (18)	1 (9)	3 (25)	1 (8)
Race and ethnicity				
African European	0 (0)	2 (18)	0 (0)	0 (0)
White	11 (100)	7 (64)	11 (92)	11 (92)
More than one	0 (0)	2 (18)	1 (8)	1 (8)
Time since HIV-1 diagnosis (years)	11 (4–37)	12 (1–15)	11 (2–29)	8 (2–26)
Time from HIV-1 diagnosis to ART (years)	0 (0–14)	1 (0–5)	0 (0–21)	0 (0–7)
Time on ART (years)	8 (4–23)	9 (1–14)	8 (2–23)	8 (2–26)
HIV subtype ^a^				
B	8 (73)	5 (45)	9 (75)	11 (92)
Non-B	0 (0)	4 (36)	2 (17)	1 (8)
Not available	3 (27)	2 (18)	1 (8)	0 (0)
CD4^+^ T cell count (cells per mm^3^)	743 (708–820)	694 (620–920)	1,027 (808–1,240)	832 (723–1,450)
HLA class I alleles ^a^				
Risk: HLA-B*07 and HLA-B*35	5 (45)	5 (45)	3 (25)	5 (42)
Protective: HLA-B*27, HLA-B*57 and HLA-B*58	1 (9)	2 (18)	2 (17)	0 (0)

Data are *n* (%) or median (range).

^a^ Individual HIV-1 subtypes and HLA class I can be found in Supplementary Table 1.

### Plasma HIV-1 RNA kinetics after ART interruption

**Fig. 2:**
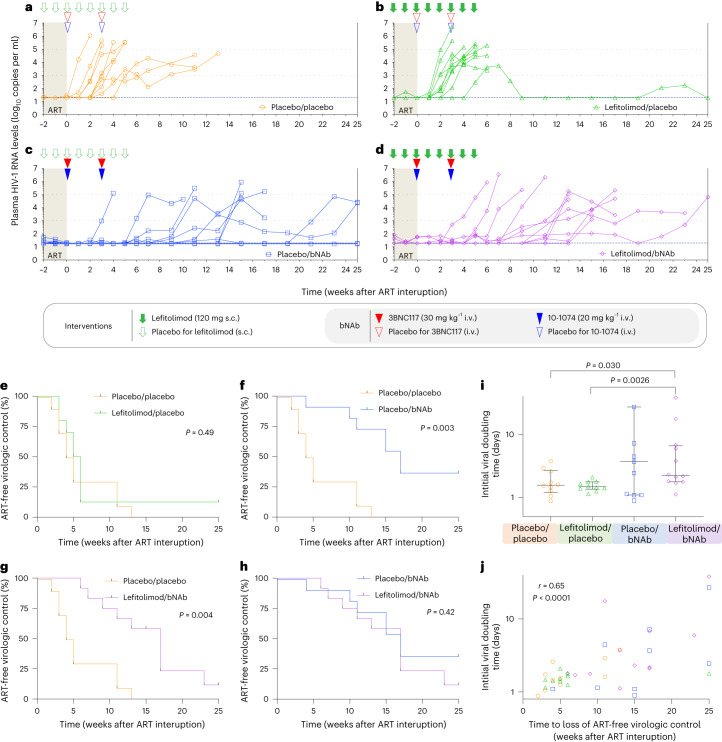
Viral kinetics and time to loss of virologic control during 25 weeks of ATI. **a**–**d**, Individual plasma HIV-1 RNA levels are shown in the four randomization groups during 25 weeks of ATI: **a**, placebo/placebo group (*n* = 10); **b**, lefitolimod/placebo (*n* = 10); **c**, placebo/bNAb (*n* = 11); and **d**, lefitolimod/bNAb (*n* = 12). Solid green arrows indicate lefitolimod injections. Solid red and blue triangles indicate 3BNC117 and 10-1074 infusions, respectively. Empty green arrows as well as red and blue triangles indicate placebo injections or infusions, respectively. Light gray shaded areas indicate time on ART, and white shaded areas indicate still interrupting ART during the 25 weeks of ATI. The three horizontal dotted lines indicates plasma HIV-1 RNA limit of quantification and 1,000 and 100,000 copies per milliliter, respectively. Viral rebound was defined as 4 weeks with plasma HIV-RNA >1,000 copies per milliliter or two consecutive measurements >100,000 copies per milliliter. **e**–**h**, Kaplan–Meier curves showing the percentage of individuals still interrupting ART during the 25 weeks of ATI. Time to loss of virologic control for the three active interventional groups compared to the placebo/placebo group: lefitolimod/placebo (**e**), placebo/bNAb (**f**) and lefitolimod/bNAb (**g**). **h**, Time to loss of virologic control for the placebo/bNAb group compared to the letifolimod/bNAb group. *P* values were calculated using the log-rank test. **i**, Dot plot of the initial viral doubling time in plasma HIV-1 RNA during ATI among the four randomization groups. The number of individuals per group is as stated for **a**–**d**, but the two individuals with completed virologic control in the placebo/bNAb group were given a high doubling time of 100 d beyond the axis to be included and to avoid skewing the data (lines at median and IQRs). *P* values comparing between groups were calculated using the two-tailed Mann–Whitney test. **j**, Correlation between time to loss of ART-free virologic control during the 25 weeks of ATI and initial viral doubling time among the four randomization groups. The number of individuals per group is as stated in **i**. *P* value was calculated using two-tailed Spearman’s correlation coefficient.

To determine the impact of the interventions on ART-free virologic control, all participants underwent a closely monitored 25-week ATI. The study’s primary endpoint was time from stopping ART to the date of meeting the criteria for loss of virologic control (defined as 4 weeks with sustained plasma HIV-1 RNA ≥1,000 copies per milliliter or two consecutive measurements >100,000 copies per milliliter). All participants in the placebo/placebo group experienced loss of virologic control, which occurred at a median of 4.5 weeks (IQR: 3.0–11) after stopping ART (Fig. 2a). In the lefitolimod/placebo group, median time to loss of virologic control was 5.0 weeks (IQR: 4.0–6.0) (Fig. 2b). One individual in this group had partial ART-free virologic control with viremia ranging from <20 to 5,500 copies per milliliter throughout the ATI (Fig. 2b). In the placebo/bNAb group, median time to loss of virologic control was 17 weeks (IQR: 11–25) (Fig. 2c). However, four of 11 participants (36%) maintained virologic control during the 25-week ATI. In the lefitolimod/bNAb group, the median time to reach criteria for viral rebound was 14 weeks (IQR: 10–17) (Fig. 2d). One individual had partial ART-free virologic control, with viremia ranging from <20 to 65,100 copies per milliliter during the ATI. All six individuals with either partial or complete ART-free virologic control tested negative for antiretroviral drugs in plasma at week 25 of the ATI. One partial controller in the placebo/bNAb group had a protective HLA class I allele (ID601: HLA*B27) (Extended Data Table 1), and one started ART less than 6 months from the presumed date of HIV-1 infection (ID142) (Extended Data Table 1).

**Extended Data Fig. 2:**
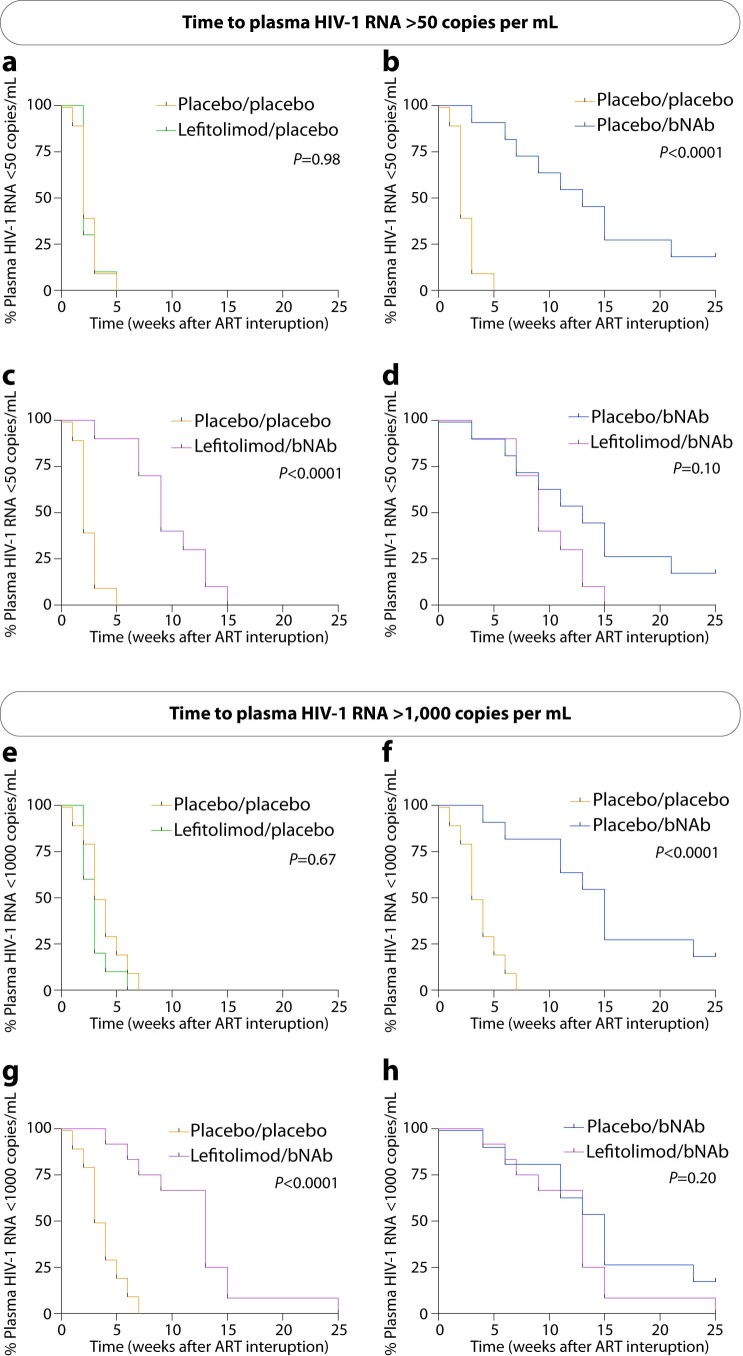
Time to plasma HIV-1 RNA above 50 and 1,000 copies/mL during 25 weeks of ATI. (**a-h**) Kaplan–Meier curves showing the percentage of individuals still interrupting ART with plasma HIV-1 RNA < 50 and <1,000 copies/mL during the 25 weeks of ATI for the three active interventional groups compared to the placebo/placebo group: Lefitolimod/placebo (**a, e**), placebo/bNAb (**b, f**) and lefitolimod/bNAb (**c, g**). Kaplan–Meier curves showing the percentage of individuals still interrupting ART with plasma HIV-1 RNA < 50 and <1,000 copies/mL during the 25 weeks of ATI for the placebo/bNAb group compared to the lefitolimod/bNAb group (**d, h**). *P* values were calculated using the log-rank test. ART, antiretroviral therapy; ATI, ART interruption; bNAb, broadly neutralizing antibody.

Although there was no difference in time to loss of virologic control between the lefitolimod/placebo group and the placebo/placebo group (*P* = 0.49) (Fig. 2e), loss of virologic control occurred significantly later in both the placebo/bNAb group (median delay, 12.5 weeks, *P* = 0.003) and the lefitolimod/bNAb group (median delay, 9.5 weeks, *P* = 0.004) compared to the placebo/placebo group (Fig. 2f,g). There was no difference in time to loss of virologic control between the placebo/bNAb and lefitolimod/bNAb group (*P* = 0.42) (Fig. 2h). Similar differences were observed when we analyzed time to first plasma HIV-1 RNA measurement >50 or >1,000 copies per milliliter (Extended Data Fig. 2). Of note, the median initial viral doubling time, defined as the trajectory of the increase of the initial slope in plasma HIV-1 RNA during ATI, was slower for the two bNAb groups compared to non-bNAb groups (Fig. 2i). Median initial viral doubling time was significantly slower for the lefitolimod/bNAb group compared to the placebo/placebo (*P* = 0.030) and lefitolimod/placebo (*P* = 0.0026) groups. The median initial viral doubling time strongly correlated with time to loss of virological control (*P* < 0.0001) (Fig. 2j).

**Extended Data Fig. 3:**
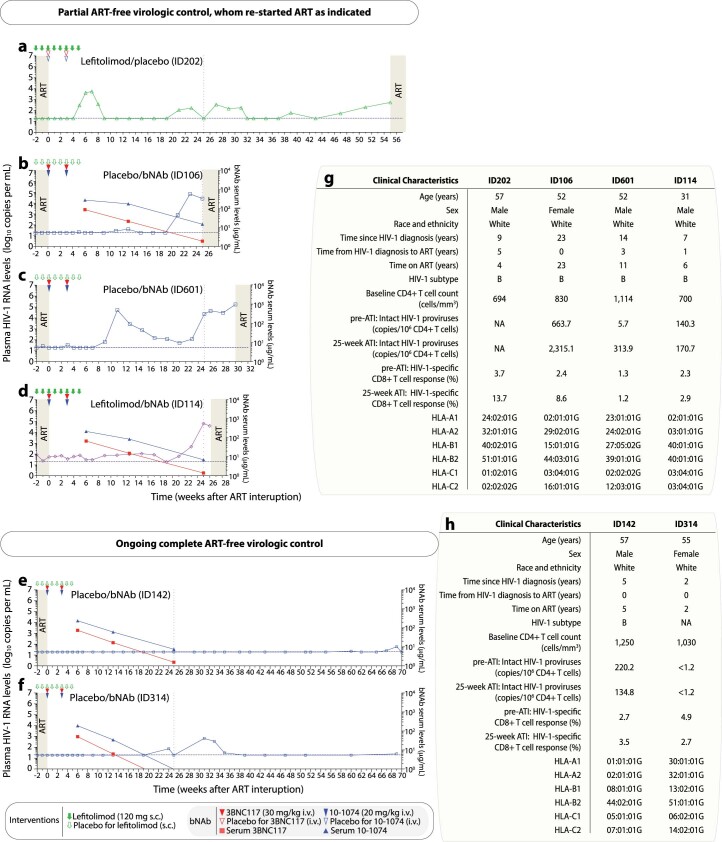
Plasma HIV-1 RNA kinetics for participants with partial and ongoing complete ART-free virologic control during the 25 weeks of ATI. (**a-d**) Individual plasma HIV-1 RNA levels are shown in the four participants (ID202, ID106, ID601 and ID114) with partial ART-free virologic control during 25 weeks of ATI, whom re-started ART as indicated. (**e, f**) Individual plasma HIV-1 RNA levels are shown in the two participants (ID142 and ID314) with ongoing complete ART-free virologic control during 25 weeks of ATI. Solid green arrows indicate lefitolimod injections. Solid red and blue triangles indicate 3BNC117 and 10-1074 infusions, respectively. Empty green arrows as well as red and blue triangles indicate placebo injections or infusions, respectively. Plasma HIV-1 RNA (group-colored symbols with line; left y-axis) and bNAb serum concentrations (3BNC117, solid red square with line; 10-1074, solid blue triangle with line; right y-axis) during ATI, ID601 did not have serum samples taken. Light grey shaded areas indicate time on ART, while white shaded areas indicate still interrupting ART. The vertical dotted line at week 25 indicates end-of-study time point. (**g-h**) Overview of the clinical characteristics for participants with partial and ongoing complete ART-free virologic control during the 25 weeks of ATI. ART, antiretroviral therapy; ATI, ART interruption; bNAb, broadly neutralizing antibody; HLA, human leukocyte antigen; NA, not available; i.v., intravenous; s.c., subcutaneous.

In the placebo/bNAb group, two of the four individuals who did not meet criteria for loss of virologic control displayed partial virologic control with viremia ranging from <20 to 70,000 copies per milliliter, whereas the other two individuals maintained complete virologic control with undetectable plasma HIV-1 RNA levels. These two individuals with undetectable viral load elected not to restart ART after the end of the ATI and continue to control under close monitoring 18 months after stopping ART (Extended Data Fig. 3). Collectively, these findings demonstrate that, in a placebo-controlled setting, administration of two bNAbs significantly delayed viral rebound and was associated with partial or complete ART-free virologic control among a subset of individuals. Lefitolimod did not impact the outcome of the ATI.

### bNAb sensitivity and serum concentrations

**Fig. 3:**
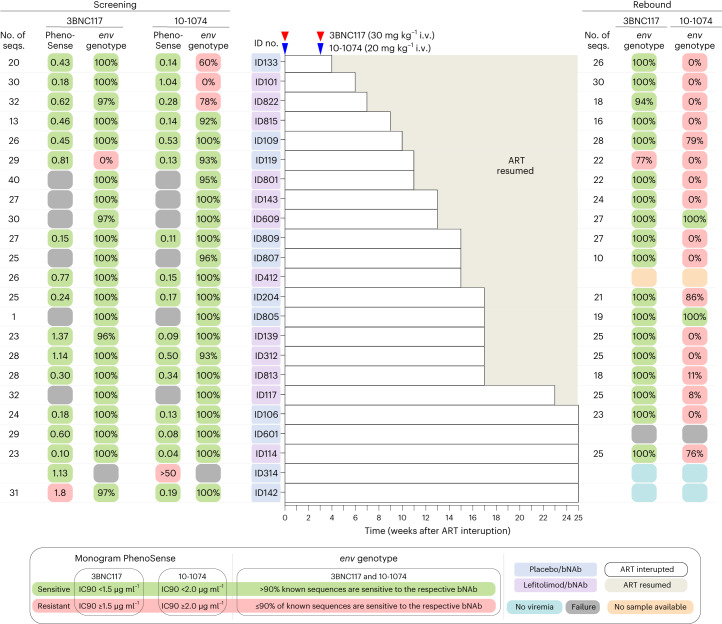
bNAb sensitivity at screening and viral rebound. bNAb sensitivity was primarily analyzed using the PhenoSense Monoclonal Antibody Assay with predefined IC_90_ thresholds for 3BNC117 (<1.5 µg ml^−1^) and 10-1074 (<2.0 µg ml^−1^) and MPI ≥98%. In six participants, the PhenoSense Assay failed, and we secondarily used proviral HIV-1 *env* sequences on a genotypic prediction algorithm with predefined thresholds of >90% known sequences are sensitive. One participant (ID142) was enrolled based upon the genotypically analysis, but post hoc phenotypic data showed an IC_90_ of 1.8 µg ml^−1^ for 3BNC117. In another participant (ID314), both the PhenoSense Assay and *env* sequencing initially failed, and we tertiary (Methods) included this individual based on the assumption that both assays failed due to a very small reservoir size, but post hoc phenotypic data showed archived 10-1074-resistant proviruses. For individuals enrolled based upon the phenotypic data, genotypical data were subsequently obtained except for one participant (ID314). If the participants had reached criteria for viral rebound before or were viremic at the end of the 25 weeks of ATI, bNAb sensitivity using plasma HIV-1 *env* sequences was analyzed using the same genotypic prediction algorithm. ID412 did not have samples available at viral rebound, and *env* sequencing failed for ID601. ID314 and ID142 had complete virologic control at the end of the 25 weeks of ATI. Solid red and blue triangles indicate 3BNC117 and 10-1074 infusions, respectively. Light gray shaded areas indicate time on ART, and white shaded boxes indicate still interrupting ART during the 25 weeks of ATI. Dark gray boxes are assay failures; orange boxes are no samples available; and dark blue boxes are no viremia at the end of 25-week ATI.

**Fig. 4:**
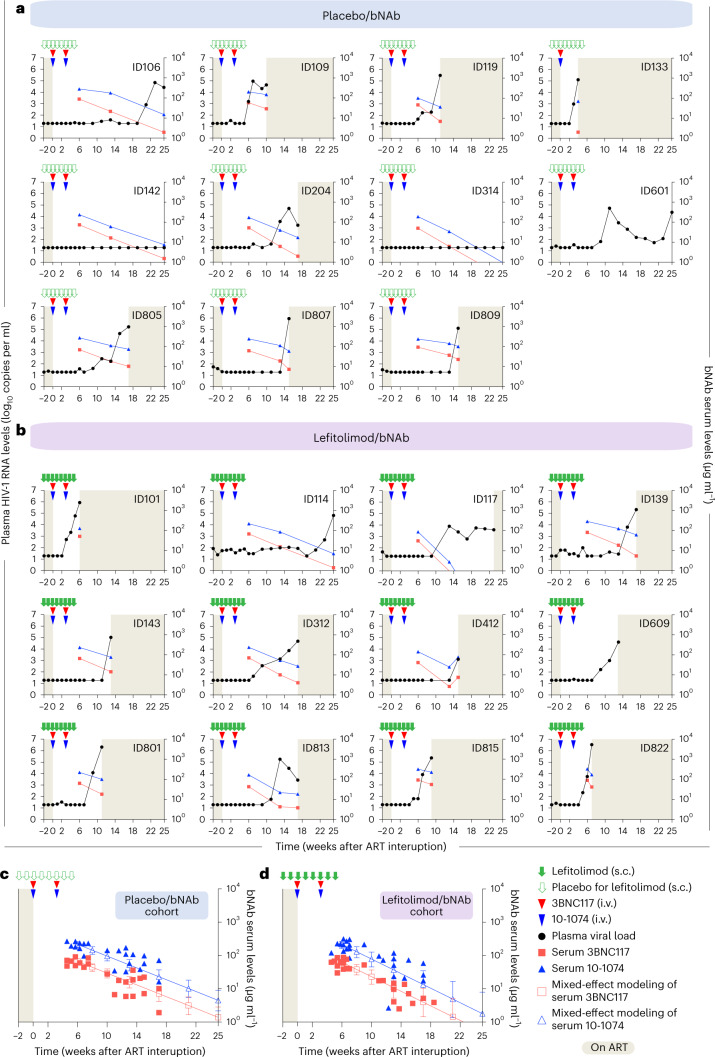
Plasma HIV-1 RNA and bNAb serum levels for the two bNAb groups. **a**,**b**, Plasma HIV-1 RNA (solid black dot with line; left *y* axis) and bNAb serum concentrations (3BNC117, solid red square with line; 10-1074, solid blue triangle with line; right *y* axis) for the placebo/bNAb group (**a**) and lefitolimod/bNAb group (**b**) during 25 weeks of ATI. Solid green arrows indicate lefitolimod injections. Solid red and blue triangles indicate 3BNC117 and 10-1074 infusions, respectively. Empty green arrows as well as red and blue triangles indicate placebo injections or infusions, respectively. Gray shaded areas indicate time on ART, and white shaded areas indicate still interrupting ART during the 25 weeks of ATI. The lower limit of quantification of plasma HIV-1 RNA was 20 copies per milliliter. In the placebo/bNAb group, serum concentration is shown for 10 individuals; ID601 did not have serum samples taken; and ID133 received only the first bNAb infusions due to a severe adverse event. In the lefitolimod/bNAb groups, serum concentration is shown for all individuals (*n* = 11) except ID609, who did not have serum samples taken. **c**,**d**, Group levels of bNAb serum concentrations for the placebo/bNAb group (*n* = 10) (**c**) and lefitolimod/bNAb group (*n* = 11) (**d**) during 25 weeks of ATI. Mixed-effect modeling with open squares and triangles with lines for mean (s.d.) 3BNC117 and 10-1074 serum concentrations, respectively, during 25 weeks of ATI.

**Extended Data Fig. 4:**
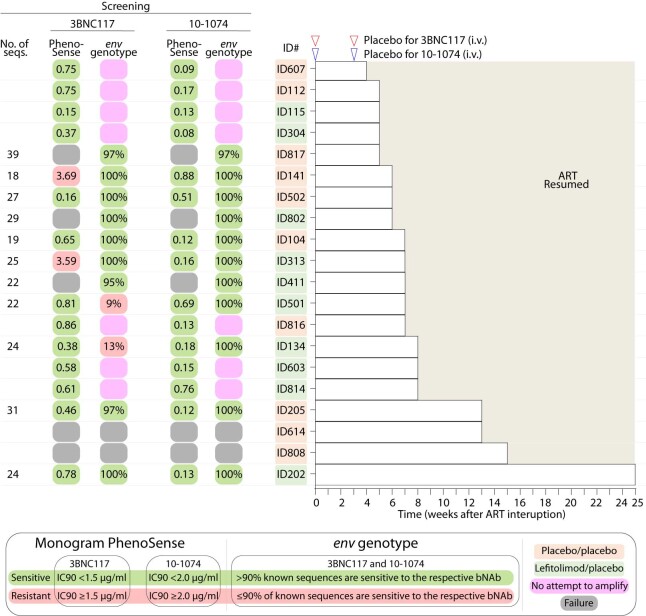
bNAb sensitivity at screening for the placebo/placebo and lefitolimod/placebo groups. bNAb sensitivity was primarily analyzed using the PhenoSense Monoclonal Antibody Assay with predefined IC90 thresholds for 3BNC117 ( < 1.5 µg/mL) and 10-1074 ( < 2.0 µg/mL) and maximum percentage inhibition (MPI) ≥ 98%. In seven participants, the PhenoSense Assay failed, and we secondarily used proviral HIV-1 envelope (*env*) sequences on a genotypic prediction algorithm with predefined thresholds of ≥90% known sequences are sensitive. Two participants (ID141 and ID313) were enrolled based upon the genotypically analysis, but post-hoc phenotypic data showed IC90 values of 3.69 and 3,59 µg/mL for 3BNC117, respectively. In another two participants (ID614 and ID808), both the PhenoSense Assay and *env* sequencing initially failed, and we tertiary-included (see Methods) these individuals based on the assumption that both assays failed due to a very small reservoir size. Empty red and blue triangles indicate placebo for 3BNC117 and 10-1074 infusions, respectively. Light grey shaded areas indicate time on ART, while white shaded boxes indicate still interrupting ART during the 25 weeks of ATI. Dark grey boxes are assay failures and pink boxes are no attempt to amplify. ART, antiretroviral therapy; ATI, ART interruption; *env*; HIV-1 envelope; IC90, concentration of bNAb required for 90% inhibition; i.v., intravenous.

The proviral reservoir’s sensitivity to neutralization by 3BNC117 and 10-1074 was assessed for each potential study participant as part of the inclusion criteria before randomization (see Methods for details). bNAb sensitivity screening was primarily done using the PhenoSense Monoclonal Antibody Assay (LabCorp–Monogram Biosciences). If phenotypic sensitivity results were inconclusive or the PhenoSense assay could not be carried out, single-genome amplification and sequencing of HIV-1 envelope (*env*) followed by genotypic sensitivity prediction was used as an alternate screening method (Fig. 3 and Extended Data Fig. 4). Among participants who received bNAb, plasma HIV *env* sequences were analyzed for known resistance mutations if viral rebound occurred (Fig. 3). As serum levels of 3BNC117 gradually declined to subtherapeutic levels, most participants in the bNAb groups experienced viral rebound while seemingly on 10-1074 monotherapy (Fig. 4a,b). Post hoc analyses demonstrated that the prevalence of genotypic 10-1074 resistance mutations increased from 13% at screening to 89% at viral rebound among bNAb-treated individuals (Fig. 3). At the time of loss of virologic control, the mean serum concentrations were 13.2 µg ml^−1^ (s.d. ± 10.6) for 3BNC117 and 73.3 µg ml^−1^ (s.d. ± 47.2) for 10-1074 in the placebo/bNAb group and 20.1 µg ml^−1^ (s.d. ± 21.0) and 88.2 µg ml^−1^ (s.d. ± 70.1), respectively, for the lefitolimod/bNAb group. Of note, in a mixed-effects model, the decline in serum bNAb concentrations after the second antibody dose was significantly faster in the lefitolimod/bNAb group compared to the placebo/bNAb group (*P* = 0.023 for 3BNC117 and *P* = 0.019 for 10-1074) (Fig. 4c,d).

Although their reservoir was predicted to be sensitive based on phenotypic screening, the three bNAb recipients (ID133, ID101 and ID822) with the shortest time to loss of virologic control (<8 weeks) were subsequently shown to harbor genotypic 10-1074-resistant proviruses at enrollment (Fig. 3). Notably, the two individuals with complete virologic control were enrolled based on their genotypic bNAb sensitivity screening, but post hoc phenotypic data indicated some level of resistance to either 3BNC117 or 10-1074 at enrollment. In summary, despite careful screening for bNAb sensitivity before enrollment, some participants appeared to harbor resistant proviruses that resulted in early viral rebound. These observations highlight the challenges with the current approaches to DNA-based bNAb sensitivity screening in PWH on long-term ART.

### Quantification of intact HIV proviruses

**Fig. 5:**
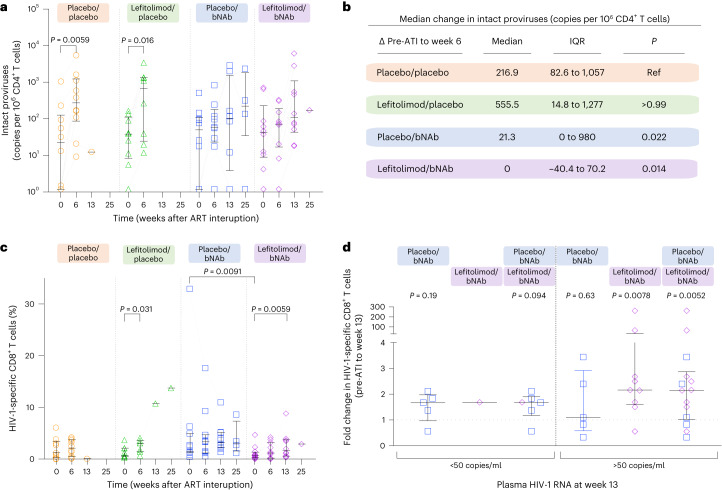
Size of the intact HIV-1 reservoir and HIV-1-specific CD8^+^ T cell immunity. **a**, Dot plot of the size of the intact HIV-1 reservoir at ATI start (day 0) and after 6, 13 and 25 weeks of ATI among individuals in the four randomization groups (lines at median and IQRs): placebo/placebo (*n* = 10); lefitolimod/placebo (*n* = 9); placebo/bNAb (*n* = 10); and lefitolimod/bNAb (*n* = 11). All weeks are shown, including weeks with no data available. *P* values comparing within groups were calculated using the paired two-tailed Wilcoxon test. Only *P* values below 0.05 are shown. **b**, Median change in the intact HIV-1 reservoir between week 6 of the ATI and pre-ATI for the four randomization groups are shown. Data are median and IQRs with the placebo/placebo group as the reference. Placebo/placebo group (*n* = 10); lefitolimod/placebo (*n* = 8); placebo/bNAb (*n* = 9); and lefitolimod/bNAb (*n* = 9). *P* values comparing between groups were calculated using the two-tailed Mann–Whitney test. **c**, Dot plot of the frequency of HIV-1-specific CD8^+^ T cells at ATI start (day 0) and after 6, 13 and 25 weeks of ATI among individuals in the four randomization groups (lines at median and IQRs): placebo/placebo (*n* = 10); lefitolimod/placebo (*n* = 9); placebo/bNAb (*n* = 11); and lefitolimod/bNAb (*n* = 12). All weeks are shown, including weeks with no data available. *P* values comparing within groups and between groups were calculated using the paired two-tailed Wilcoxon test and two-tailed Mann–Whitney test, respectively. Only *P* values below 0.05 are shown. **d**, Dot plot of median fold change in HIV-1-specific CD8^+^ T cells after 13 weeks of ATI among individuals with either plasma HIV-1 RNA below (left) or above (right) 50 copies per milliliter in the two randomization groups receiving bNAb combination (lines at median and IQRs). Plasma HIV-1 RNA below 50 copies per milliliter: placebo/bNAb (*n* = 5) and lefitolimod/bNAb (*n* = 1); plasma HIV-1 RNA above 50 copies per milliliter: placebo/bNAb (*n* = 5) and lefitolimod/bNAb (*n* = 9). *P* values comparing within groups were calculated using the paired two-tailed Wilcoxon test.

**Extended Data Fig. 5:**
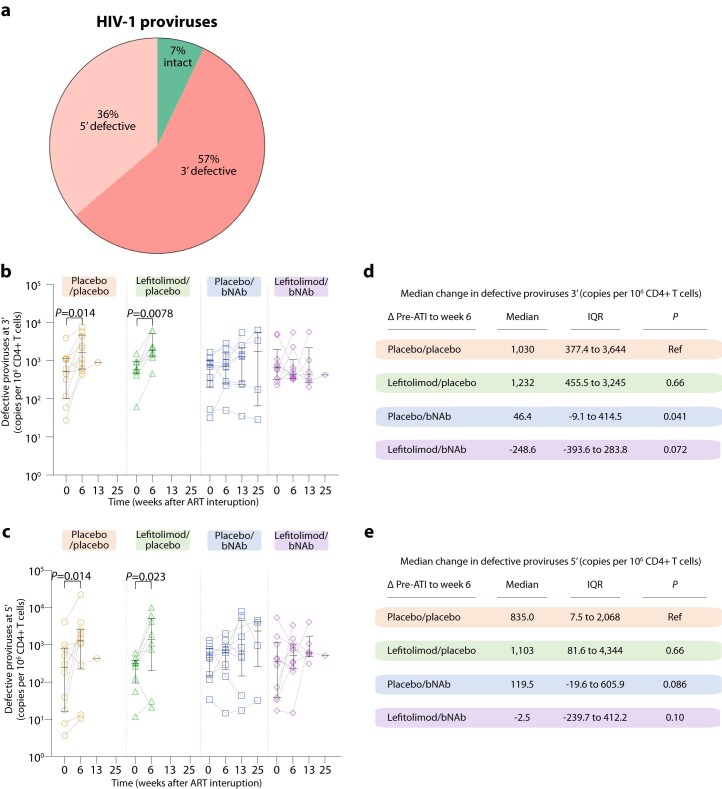
Fraction and size of the defective HIV-1 proviruses. Mean fraction of intact, 3′ and 5′ defective HIV-1 proviruses per 10^6^ CD4 + T cells from 39 individuals at ATI start (day 0) (**a**). The size of the 3’ (**b**) and 5’ (**c**) defective HIV-1 reservoir at ATI start (day 0) and after 6, 13 and 25 weeks of ATI among individuals in the four randomization groups (lines at median and IQRs). Placebo/placebo group (n = 10); lefitolimod/placebo (n = 9); placebo/bNAb (n = 10); and lefitolimod/bNAb (n = 11). All weeks are shown, including weeks with no data available. Median change in the 3’ (**d**) and 5’ (**e**) defective HIV-1 reservoir between week 6 of the ATI and pre-ATI for the four randomization groups are shown. Data are median and IQRs with the placebo/placebo group as the reference. Placebo/placebo group (n = 10); lefitolimod/placebo (n = 8); placebo/bNAb (n = 9); and lefitolimod/bNAb (n = 9). *P* values comparing within groups (only *P* values below 0.05 are shown) and between groups were calculated using the paired two-tailed Wilcoxon test and two-tailed Mann–Whitney test, respectively. ART, antiretroviral therapy; ATI, ART interruption; IQR, interquartile ranges; bNAb, broadly neutralizing antibody.

We used a digital droplet PCR (ddPCR) assay (Intact Proviral DNA Assay (IPDA)) to estimate the proportion of intact proviruses in CD4^+^ T cells. For some (9/31) individuals with subtype B for whom IPDA failed, as well as for the individuals with non-B subtypes (*n* = 6), we used alternative primer/probe or an IPDA-like duplexed ddPCR (3dPCR) assay (Supplementary Table 1) to estimate the intact reservoir^6^. The number of intact proviruses varied among individuals at baseline (range: 1.18–2,576.62 copies per 10^6^ CD4^+^ T cells; Extended Data Table 1) but did not differ between groups (*P* = 0.50). The mean proportion of intact proviruses at baseline was 7% (Extended Data Fig. 5a). Six weeks after starting ATI, the placebo/placebo and lefitolimod/placebo groups showed significant increases in circulating intact and defective proviruses (Fig. 5a and Extended Data Fig. 5b,c). At this time, 16 of 20 individuals in the placebo/placebo and lefitolimod/placebo groups had reached criteria for loss of virologic control (Fig. 2a,b). The largest median increase in intact and defective proviruses was observed among the lefitolimod/placebo group (Fig. 5b and Extended Data Fig. 5d,e). The median increase in intact proviruses among the placebo/placebo group was significantly higher than the median changes in the two bNAb groups (Fig. 5b). Similar changes were observed for defective proviruses (Extended Data Fig. 5d,e). In contrast, we found no significant increases in intact or defective proviruses in the bNAb recipients over the same time period, suggesting that the two bNAbs restricted HIV-1 replication during ATI. In summary, we observed that the frequency of CD4^+^ T cells harboring intact proviruses expanded after ATI in non-bNAb recipients but that bNAb administration prevented reservoir expansion during the early phase of the ATI.

### HIV-1-specific T cell immunity

**Extended Data Fig. 6:**
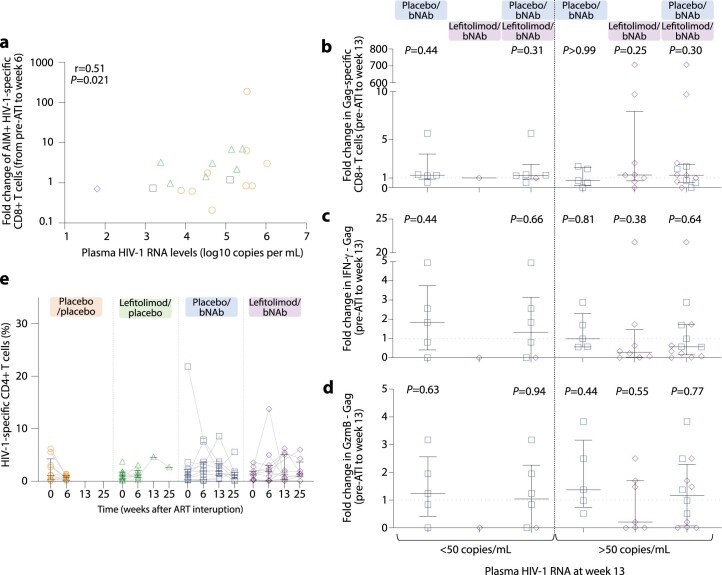
HIV-1-specific CD8+ and CD4+ immune responses during ATI. (**a**) Correlation between fold change of HIV-1-specific CD8 + T cell response from pre-ATI to week 6 of ATI and measurable plasma HIV-1 RNA at week 6 with for participants with plasma HIV-1 RNA > 50 copies/mL. Placebo/placebo group (n = 9); lefitolimod/placebo (n = 7); placebo/bNAb (n = 2); and lefitolimod/bNAb (n = 1). *P* value calculated using two-tailed Spearman’s correlation coefficient. (**b-d**) Dot plot of median fold change in Gag-specific CD8 + T cells and Gag-induced INF-γ or GzmB responses after 13 weeks of ATI among individuals with either plasma HIV-1 RNA below (left panel) or above (right panel) 50 copies per mL in the two randomization groups receiving bNAb combination (lines at median and IQRs). Plasma HIV-1 RNA below 50 copies/mL: Placebo/bNAb (n = 5); and lefitolimod/bNAb (n = 1), or above 50 copies/mL: Placebo/bNAb (n = 5); and lefitolimod/bNAb (n = 7 to 9). *P* values comparing within groups were calculated using the paired two-tailed Wilcoxon test. (**e**) Dot plot of the frequency of HIV-1-specific CD4 + T cells at ATI start (day 0) and after 6, 13 and 25 weeks of ATI among individuals in the four randomization groups (lines at median and IQRs). All weeks are shown, including weeks with no data available. Placebo/placebo group (n = 10); lefitolimod/placebo (n = 9); placebo/bNAb (n = 11); and lefitolimod/bNAb (n = 12). *P* values comparing within groups were calculated using the paired two-tailed Wilcoxon test. Only *P* values below 0.05 are shown. ART, antiretroviral therapy; ATI, ART interruption; bNAb, broadly neutralizing antibody; IQR, interquartile ranges.

The median frequency of HIV-1-specific CD8^+^ T cells increased from pre-ATI to week 6 in all four treatment groups as measured by the activation-induced marker (AIM) assay, but the increase was significant only for the lefitolimod/placebo group (Fig. 5c). This change in HIV-1-specific CD8^+^ T cell responses from pre-ATI to week 6 was significantly correlated with measurable plasma HIV-1 RNA at week 6 (*r* = 0.51, *P* = 0.021) (Extended Data Fig. 6a). The frequency of HIV-1-specific CD8^+^ T cells also increased significantly from the pre-ATI timepoint to week 13 for the lefitolimod/bNAb group (*P* = 0.0059). To disentangle increased antigen exposure from a bNAb-mediated vaccinal effect as potential cause of increased HIV-1 specific immunity, we separated bNAb-treated individuals in a post hoc analysis into two groups based on whether their plasma HIV-1 RNA was below or above 50 copies per milliliter during the ATI. As expected, bNAb-treated individuals with plasma HIV-1 RNA above 50 copies per milliliter had higher HIV-1-specific CD8^+^ T cell responses at week 13 compared to pre-ATI (*P* = 0.0052) (Fig. 5d). We also observed a non-significant increase in total HIV-1-specific CD8^+^ T cell responses among bNAb-treated individuals whose plasma HIV-1 RNA was below 50 copies per milliliter at week 13 (*P* = 0.094) (Fig. 5d), but we found no detectable changes in Gag-specific CD8^+^ T cell responses or in Gag-induced interferon (IFN)-γ or granzyme B (GzmB) responses (Extended Data Fig. 6b–d). The frequency of HIV-1-specific CD4^+^ T cells remained relatively stable over time in all four groups (Extended Data Fig. 6e). Thus, whereas HIV-1-specific CD8^+^ T cell responses increased with increasing plasma HIV-1 RNA during the ATI, bNAb recipients who maintained plasma HIV-1 RNA <50 copies per milliliter during 3 months of ATI did not experience substantial increases in their HIV-1-specific CD8^+^ T cell responses that would be suggestive of a bNAb-mediated vaccinal effect or low-level antigen exposure during bNAb-mediated viral suppression.

### Safety

**Extended Data Fig. 7:**
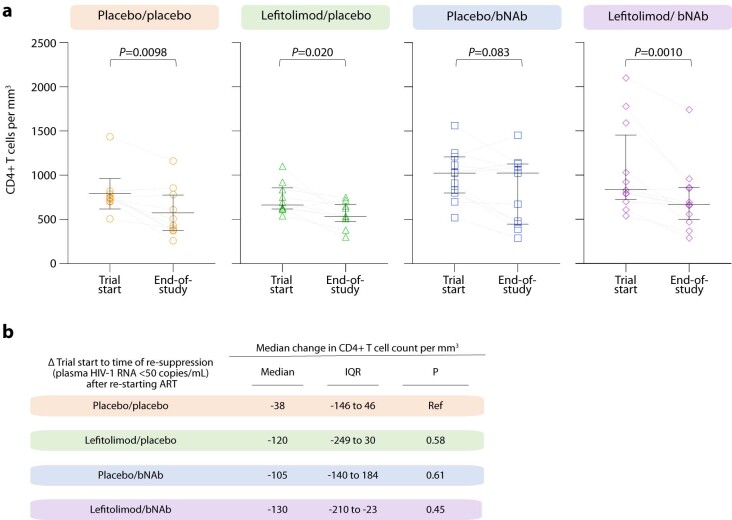
CD4 + T cell count at trial start and end-of-study. (**a**) Dot plots of the CD4 + T cell count in the four groups at trial start (week -2) and end-of-study (lines at median and IQRs). Placebo/placebo group (n = 10); lefitolimod/placebo (n = 10); placebo/bNAb (n = 11); and lefitolimod/bNAb (n = 12). *P* values comparing within groups were calculated using the paired two-tailed Wilcoxon test. (**b**) Median change in CD4 + T cell count in the four groups between time point of viral suppression (plasma HIV-1 RNA < 50 copies/mL) and trial start are shown. Data are median and IQRs with the placebo/placebo group as the reference. Placebo/placebo group (n = 8); lefitolimod/placebo (n = 7); placebo/bNAb (n = 6); and lefitolimod/bNAb (n = 10). *P* values between groups were calculated using the two-tailed Mann–Whitney test. bNAb, broadly neutralizing antibody; IQR, interquartile ranges.

Both lefitolimod and bNAbs were overall safe. A total of 253 adverse events (AEs) were registered, of which 94 were determined to be unrelated to any investigational drug, including placebo (Extended Data Tables 2 and 3). Of 81 AEs considered related to lefitolimod, 75 were graded mild and six were graded moderate, with the most common AEs being injection site reaction (*n* = 39) and fatigue (*n* = 6). Of 14 AEs considered related to bNAb, 11 were graded mild, two were graded moderate and one was graded severe, with fatigue (*n* = 5) being the most commonly reported AE. An infusion-related reaction categorized as severe and related to the second 3BNC117 infusion (ID133) resolved with fluid therapy, antihistamine and corticosteroid without any sequelae. Another AE, not related to study drugs, was categorized as severe, where a participant (ID813) had a vasovagal reaction just after insertion of the intravenous needle before bNAb infusion. The reaction resolved with fluid therapy without any sequelae. Median CD4^+^ T cell counts decreased from study start to end of study in three of the four groups (Extended Data Fig. 7) but was stable in the placebo/bNAb group. Among participants who re-initiated ART after viral rebound, all but one (ID807) achieved viral re-suppression after a median of 71 d (IQR: 38–116), and median CD4^+^ T cell counts had recovered to pre-ATI levels in all four groups (Extended Data Fig. 7b).

## Discussion

In this randomized, placebo-controlled HIV-1 cure trial among PWH on long-term ART who screened sensitive to the study bNAbs, we found that two doses of 3BNC117 and 10-1074 in the setting of ATI led to a significant delay in viral rebound compared to placebo. We also observed that 36% of individuals in the placebo/bNAb group compared to none in the placebo group maintained partial or complete virologic control at the end of the 25-week ATI. There were no differences in time to viral rebound or change in reservoir size between the placebo/placebo and the lefitolimod/placebo group or between the placebo/bNAb and lefitolimod/bNAb group. Thus, although there was no added clinical or immunological benefit of combining lefitolimod with bNAbs, antibody treatment alone led to a significant delay in viral rebound, and some bNAb-treated individuals experienced long-term ART-free virologic control.

Single-arm trials show that bNAb-sensitive PWH on long-term ART receiving 3–8 infusions of 3BNC117 and 10-1074 maintain prolonged viral suppression in the absence of ART^27–29^. Notably, our study was not designed to evaluate the ability of bNAbs to replace ART as a means to durably suppress viral replication but, rather, to address an important HIV-1 cure hypothesis: could a TLR9 agonist alone or in combination with bNAbs increase the chance of long-term ART-free control? We addressed this question using the most rigorous study design: a factorial, randomized, placebo-controlled, double-blinded trial. We administered bNAbs only twice—one infusion before stopping ART and a second infusion 3 weeks after ATI—but found that time to viral rebound after the last infusion was similar to that seen in previous multi-dose trials^27–29^.

Of note, we administered 10-1074 at 20 mg kg^−1^ compared to 30 mg kg^−1^ for 3BNC117 to compensate for the longer half-life of 10-1074 compared to 3BNC117, but, despite differential dosing, 3BNC117 was cleared from plasma faster than 10-1074, leading to a period of effective monotherapy with the latter. Consistent with the pharmacokinetics, 89% of our participants developed new 10-1074 resistance mutations at viral rebound indicative of residual 10-1074 monotherapy, which has also been reported in other trials. Collectively, these studies demonstrate that 10-1074 monotherapy is not sufficient to maintain virologic suppression^34^.

In our study, 18% of participants (2/11) in the placebo/bNAb group maintained complete virologic control (HIV RNA <20 copies per milliliter) throughout the 25-week ATI, and both elected to continue off ART after the ATI ended. In previous trials using the same bNAb combination, 0% (0/7)^28^, 11% (1/9)^27^ and 17% (2/12)^29^ of bNAb-sensitive individuals maintained complete virologic control during ATI. Furthermore, in our study, bNAb treatment changed the trajectory of viral rebound as evidenced by slower plasma virus doubling time during the ATI, which is suggestive of stronger immune pressure against the replicating virus in bNAb recipients.

Although virologic or immunological features characteristic of post-bNAb controllers have yet to be identified, pre-ATI HIV-1 reservoir size was seven-fold lower in post-ART controllers compared to non-controllers in a cohort study^35^. Further analysis showed that the reservoir in post-treatment controllers remained stable during ATI^36^. We found that the frequency of intact HIV-1 in CD4^+^ T cells remained stable during bNAb-mediated suppression of plasma viral load but expanded upon loss of virologic control. The two individuals who maintained complete virologic control maintained a stable reservoir throughout the ATI.

As expected, we found that HIV-1-specific CD8^+^ T cell responses increased during viral rebound. We also observed a modest increase in HIV-1-specific CD8^+^ T cell responses among bNAb recipients who maintained viral suppression, but, in contrast to a previous report^37^, we observed no change in Gag-specific CD8^+^ T cell responses or Gag-induced cytokine release. Thus, there was no clear indication of a vaccinal effect^31,37–42^ in the present study. The failure to broadly stimulate cellular immunity could be due to low amounts of antigen at the time of bNAb administration because bNAb dosed at ART initiation among newly diagnosed viremic individuals induced sustained strong HIV-1-specific CD8^+^ T cell responses^6,30^. Potent HIV-1-specific CD8^+^ T cell immunity is critical for maintaining virologic suppression in humans and bNAb-treated macaques^31,32,43^. Whether increases in HIV-1-specific cellular immunity after bNAb administration contributed to virologic control in the present study is unclear, but it seems plausible that a combined restriction of outgrowth from the viral reservoir, along with potent cellular responses, contributed to long-term ART-free control in some individuals.

The combination of 3BNC117 and 10-1074 neutralizes a broad range of HIV-1 subtypes ex vivo^44^, but bNAb treatment is still challenged by archived resistant mutations^29,45^. To address this issue, we pre-screened all participants for bNAb sensitivity using both genotypic and phenotypic approaches. However, neither bNAb sensitivity analysis accurately predicted time to viral rebound, adding further evidence of the challenge of effective screening for bNAb sensitivity. This finding is in line with other recent studies, which indicate that currently available bNAb sensitivity testing of the proviral reservoir does not accurately predict the clinical outcome of bNAb treatment^6,29^. Of note, the average serum concentrations of 3BNC117 at viral rebound were slightly higher than previously reported, where viral rebound generally occurred after 3BNC117 decreased below 10 μg ml^−1^ (refs. ^27,29^). However, both studies were relatively small, and serum antibody concentrations were measured by different methods.

Lefitolimod alone or in combination with bNAbs did not significantly impact time to viral rebound or any of the immunological or virologic outcomes in this trial. Unexpectedly, co-administration of lefitolimod led to a faster decline in bNAb serum concentrations^24,25^. Lefitolimod administration has been shown to increase proportions of CD16^+^ NK cells among PWH^12–14^, which theoretically could facilitate increased binding and faster clearance of bNAbs. Of note, a faster decline in serum concentration of an Fc-modified version of PGT121, a bNAb with similar properties to 10-1074, was also observed when it was co-administered with a TLR7 agonist in NHPs^26^. Anti-drug antibodies (ADAs) against anti-HIV-1 bNAbs rarely develop in humans, and, when they do, the presence of ADA has discernible impact on bNAb elimination kinetics^44^.

The combination of a TLR7 agonist and PGT121 was shown to induce long-term ART-free virologic control among 34–45% of NHPs^24,46^, so why did a TLR9 agonist in combination with two bNAbs fail to produce similar effects in humans? Potential explanations include the lower degree of fitness and greater bNAb sensitivity of simian–human immunodeficiency virus (SHIV) versus HIV-1, a smaller reservoir in macaques and very early ART initiation (day 7 or day 9 after infection) in the NHP model. In contrast, our study population consisted mainly of people who started ART during the chronic phase of infection and had large genetically diverse reservoirs with varying degrees of bNAb sensitivity. Early ART initiation and bNAb sensitivity are two key parameters that determine the outcome of ATIs in bNAb studies in humans and NHPs. In two complementary studies with NHPs that started ART later (day 14 and day 365 after infection), the combination of TLR7 agonist and one or more bNAbs tended to be associated with a lower frequency of post-intervention control (0%, 22% and 50%) than in the studies where animals started ART at day 7 or day 9 after infection^25,26^. Another potential explanation is differences in immune signaling between TLR7, which recognizes viral single-stranded RNA, and TLR9, which recognizes unmethylated DNA with CpG motifs^10^. Although both TLR7 and TLR9 are expressed in the endosomal compartment and signal via myeloid differentiation factor 88 (MyD88), there are notable differences^10^. In human peripheral blood mononuclear cells (PBMCs), TLR7 agonists induce a rapid burst of type I IFN transcripts, whereas TLR9 agonists trigger a slower but sustained expression of type I IFN^47^. The subtypes of induced type I IFNs also differ between the two agonists. Collectively, these experimental and biological differences may explain the divergent results between macaque and human HIV-1 cure trials.

Despite lefitolimod’s well-documented ability to enhance DC-mediated cross-presentation, upregulate IFN-stimulated genes and activate multiple innate and adaptive immune cell subsets^12–15^, we observed no discernible impact of TLR9 agonist treatment on T cell immunity. Our findings may indicate that such immunostimulatory molecules may not effectively boost HIV-1-specific immunity unless substantial amounts of HIV-1 antigen are concurrently present in the same anatomical location. This hypothesis gains support from recent discoveries in cancer research, wherein intra-tumoral injection of cytokines or TLR9 agonists has demonstrated substantially greater effects on tumor size and tumor-specific immune responses compared to the same therapies administered s.c. or i.v.^48,49^. However, ongoing trials will yield additional insights into the potential of an HIV-1 cure strategy that centers on the combination of bNAbs with immunostimulatory agents, such as other TLR agonists, pegylated interferon-a2b or IL-15 super-agonists. These trials will determine whether this approach can induce post-treatment control or if it necessitates a re-evaluation of our current strategy.

The reported results have limitations and may not be generalizable to all PWH. Specifically, our ability to predict proviral bNAb sensitivity based on the PhenoSense assay or sequence analysis is limited. Undetected bNAb resistance leading to treatment failure may have clinical implications, particularly if bNAbs were to replace modern ART regimens as suppressive antiviral treatment or if used in combination with the new long-acting small-molecule-based injectable antivirals. Most enrolled participants were male. In contrast to some bNAb trials^28^, we did not exclude individuals who initiated ART during the chronic phase of infection. Although this inclusive approach made our study population more representative of the overall population, it might also have contributed to raising the bar for success, as PWH who started ART during the acute/early phase of infection harbor smaller and less diverse HIV-1 reservoirs and may be more likely to become post-treatment controllers^50–52^.

Although lefitolimod and bNAbs were overall safe, the CD4^+^ T cell count dropped in response to recrudescent viremia. Reassuringly, once plasma HIV-1 RNA was re-suppressed, the CD4^+^ T cell counts returned to pre-ATI levels. We also found that the size of the HIV-1 reservoir expanded at rebound. In a previous study^53^, 22 PWH interrupted ART for a median of 4 months and were followed for ~2 years after restarting ART. The investigators found no difference in HIV-1 reservoir size from pre-ATI and until the last follow-up timepoint. Thus, although viral rebound might cause transient CD4^+^ cell count declines and HIV-1 reservoir size increases, the long-term clinical implications of these changes, if any, appear to be limited. Of note, one individual in this study (ID807) did not achieve rapid viral re-suppression despite having therapeutic ART drug concentrations and no evidence of emerging or archived resistance mutations. We speculate that this case of slow viral resuppression could be due to clonally expanded defective proviruses, which were recently reported to be a potential source of non-suppressible viremia among fully ART-adherent individuals^54^.

In conclusion, although there was no added benefit of combining a TLR9 agonist with bNAbs compared to bNAbs alone, this was, to our knowledge, the first placebo-controlled, double-blinded trial to show prolonged ART-free virologic control during ATI in individuals receiving just two doses of bNAbs. This finding provides further support for investigating bNAbs as a component in long-acting treatment and HIV-1 curative strategies. However, additional interventions, optimization of bNAbs and bNAb drug combinations^55,56^ and/or administration of bNAbs in the setting of higher antigen load will likely be needed to achieve ART-free HIV-1 remission in the majority of PWH on long-term suppressive ART.

## Methods

### Study design

This was a phase 2a, investigator-initiated, randomized, placebo-controlled, double-blinded international multicenter trial enrolling at six sites in Denmark (Aalborg, Aarhus, Gødstrup, Hvidovre, Odense and Rigshospitalet); one site in Oslo, Norway; and one site in Melbourne, Australia (EudraCT: 2018-001165-16). The first participants were enrolled on 16 May 2019. Before any study-related procedures, written informed consent was obtained from the participants. Participants were randomized into one of four groups— placebo/placebo, lefitolimod/placebo, placebo/bNAb or lefitolimod/bNAb—in a 1:1:1:1 ratio (Fig. 1a,b). Sex of participants was determined based on self-reporting. The planned sample size was 48 participants. Study procedures were unfortunately severely impacted by the coronavris disease 2019 (COVID-19) pandemic, and enrollment had to be paused for longer periods in 2020 and 2021. Due to expiration dates of the study drugs, enrollment closed on 5 November 2021 with 46 of the 48 planned participants enrolled. Screening took place 2–20 weeks before entering the trial. Study participants remained on ART for the first 2 weeks (week −2 to week 0) of the 8-week interventional period. The 25-week ATI started at week 0; thus, administration of study medication ended in week 5 of the ATI. Regular follow-up visits continued for 20 weeks (weeks 6–25) after the last dose of the study medication. Participants who resumed ART during ATI were followed at 4-week intervals until plasma HIV-1 RNA was <50 copies per milliliter. The study was conducted in accordance with Good Clinical Practice and is reported in accordance with the CONSORT 2010 statement^57^. The protocol was approved by the Danish Medicine Authorities (2018092874) and the Norwegian Medicines Agency (20/16305-25) as well as the National Committee on Health Research Ethics in Denmark (1-10-72-292-18), the Regional Committee on Medical and Health Research Ethics in Norway (184485) and the Alfred Human Research Ethics Committee in Australia (project 258/20). Study data were collected and managed in Research Electronic Data Capture (REDCap) electronic data capture tools hosted at the Clinical Trial Unit, Department of Clinical Medicine, Aarhus University in Aarhus, Denmark^58,59^. The study was monitored by the Danish Good Clinical Practice Units (https://gcp-enhed.dk/english/) in Denmark and Australia and by the Section for Monitoring, Clinical Trial Unit, Oslo (https://www.ous-research.no/ctu/) in Norway from screening to the final visit.

### Participants

PWH were aged 18–65 years and on ART for at least 18 months, with plasma HIV-1 RNA <50 copies per milliliter for at least 15 months and a CD4 T cell count >500 cells per mm^3^. Detailed inclusion/exclusion criteria can be found in the study protocol: TITAN-001, version 3.0, 2 July 2021 (Supplementary Information). Participants were reimbursed for transport expenses relating to the study and compensation for lost earnings during study visits but otherwise did not receive any financial compensation for participating in the study.

The sample size calculation was based on the primary endpoint: time to viral rebound during ATI. Time from stopping ART to loss of virological control was compared in the four randomization groups. If loss of virological control occurred, the date of the last measurement of plasma HIV-1 RNA ≥1,000 copies per milliliter or confirmed >100,000 copies per milliliter was defined as ‘date of viral rebound’. Using a two-sample comparison of means with an s.d. of 11 d^1,4^, 10 evaluable participants in each of the two groups would have 90% power to detect a ≥16-d difference in time to viral rebound at a 5% significance level. To accommodate for dropouts, we aimed for 12 participants in each group. We considered a two-sided α value of less than 0.05 to be significant, with no adjustments made for multiple comparisons. We used the IPDA and d3PCR assays as our primary reservoir measurement, as intact proviral DNA is superior to total HIV-1 DNA in terms of estimating the intact HIV-1 reservoir^6^. Protocol amendments did not affect the analysis plan besides the reservoir size analyses described above.

### Randomization

The Clinical Trial Unit at Aarhus University generated the randomization sequence using permuted blocks of four or eight by computer-generated random numbers without stratification to sex and age. Randomization assignment was provided to each site using REDCap.

### Blinding

Participants, study physicians and nurses handling administrations of the study drugs, as well as those individuals doing the analyses, were blinded to interventions. Only the pharmacy and study personnel preparing the study drugs were unblinded to interventions.

The placebo for lefitolimod, 3BNC117 and 10-1074 was sterile physiological saline. Lefitolimod or placebo for lefitolimod was provided to the study physicians and nurses handling the injections as 4× 2-ml syringes. Placebo for 3BNC117 and 10-1074 or the bNAbs was administered by the study physicians and nurses handling the infusions as 250-ml piggy bags. Labeling contained only information of the study visit and an expiration date and time irrespective of either placebo or study drug.

### Procedures

Lefitolimod or placebo was administered s.c. at a dose of 120 mg once weekly for the first 8 weeks from week −2 to week 5. Lefitolimod dosing was based on previously observed effects in clinical trials^12,13^. 3BNC117 (30 mg kg^−1^) and 10-1074 (20 mg kg^−1^) were sequentially administered as i.v. infusions over 60 min at weeks 0 and 3. The dosing of bNAbs was based on previously observed antiviral efficacy in clinical studies^27–29,34,44,60,61^. The infusions were performed in the Clinical Research Units at Aarhus University Hospital, Copenhagen University Hospital–Hvidovre or Rigshospitalet in Denmark; at the Clinical Research Section at the Department of Infectious Diseases, Oslo University Hospital in Norway; and in the Medical Day Unit at the Alfred Hospital, in Melbourne, Australia. Follow-up visits were conducted at the respective outpatient clinics of participating hospitals. End of study was defined as the timepoint for reaching criteria for viral rebound or week 25, whichever came first. Blood samples were collected at weeks −2, −1, 0, 1, 2, 3, 4, 5, 6, 7, 9, 11, 13, 15, 17, 19, 21, 23 and 25 after ATI depending on when the participants reached criteria for viral rebound. Blood samples were processed within 4 h of collection, and serum and plasma samples were stored at −80 °C. PBMCs were isolated by density gradient centrifugation and cryopreserved (−150 °C) in FBS with 10% DMSO. Clinical safety assessments included directed physical examinations and vital sign measurement if indicated and review of AEs and concomitant medications at every visit and reported until the end of study (Fig. 1a). The Common Terminology Criteria for Adverse Events (CTCAE) version 5.0 grading scale was used to grade AEs. A safety monitoring committee was established to monitor the blinded safety data of the trial. Safety biochemistry was taken at weeks −2, 0, 3, 6, 9, 13 and 25 after ATI. CD4^+^ T cell counts were measured on weeks −2, 0, 1, 3, 5, 6, 9, 13, 17, 21 and 25. After resumption of ART, we monitored CD4^+^ T cell count every fourth week until plasma HIV-1 RNA levels were undetectable (<50 copies per milliliter).

### 25-week analytical treatment interruption

At week 0, participants were instructed to discontinue ART the following day and continue off ART for 25 weeks unless one of four criteria was met. Details can be found in the study protocol.

### Plasma HIV-1 RNA measurements

Plasma HIV-1 RNA levels were measured with standardized clinical assays at every visit. After resumption of ART, we monitored plasma HIV-1 RNA every fourth week until levels were undetectable (<50 copies per milliliter).

### Doubling time of plasma HIV-1 RNA

The initial increase in plasma HIV-1 RNA was calculated based on the first two consecutive plasma HIV-1 RNA measurements that increased, and the second measurement had to increase by 1,000 copies per milliliter compared to the previous measurement. The slope of this increase was calculated using a linear regression model.

### bNAb sensitivity

bNAb sensitivity prediction was performed at screening for all participants primarily using the LabCorp–Monogram Biosciences PhenoSense HIV Monoclonal Antibody Assay on proviral HIV-1 DNA. bNAb sensitivity was a pre-specified inclusion criterion. For the PhenoSense assay, sensitivity was determined based on the concentration of bNAb required to inhibit viral replication by 90% (IC_90_). The sensitivity cutoffs were pre-defined as IC_90_ <1.5 for 3BNC117 and <2.0 µg ml^−1^ for 10-1074 combined with a maximum percent inhibition (MPI) observed at the highest bNAb concentration tested ≥98%. If the PhenoSense assay did not yield a result (for example, due to insufficient amplification), *env* sequences obtained by single-genome amplification (SGA) of proviral HIV-1 DNA were secondarily analyzed using a genotypic prediction algorithm. For this genotypic assay, the pre-defined cutoff was >90% of sequences being sensitive to the bNAb for the proviral reservoir to be classified as ‘sensitive’. If the *env* sequencing failed, the participant could still be enrolled based on the assumption of a lower HIV-1 reservoir size among these individuals (see the study protocol).

### Proviral HIV-1 *env* sequencing

For screening of participants who failed the PhenoSense assay and post hoc for all participants receiving the bNAb combination, SGA, sequencing and genotypic analysis were performed on proviral HIV-1 *env*. DNA was isolated from PBMCs using the DNeasy Blood and Tissue Kit (Qiagen), and Platinum Taq DNA Polymerase (Invitrogen) was used for SGA (<30% of wells containing *env*). The *env* amplification was conducted in 10-µl reactions as nested PCRs. *env* amplification was, in most cases, first attempted with primers listed in the first box (Supplementary Table 4).

For participants in whom these primers did not amplify *env*, alternative primer sets and combinations were tested (second box, Supplementary Table 4). For a few of the participants, near full-length (NFL) HIV-1 sequencing was conducted to obtain sequences that could be used for individual *env* primer design. NFL amplification was conducted as SGA using Platinum Taq DNA Polymerase High Fidelity (Invitrogen) in 10-µl reactions. If the NFL sequences were resistant to one or both bNAbs, further analysis and amplification was not conducted.

All primer sequences, an overview of which primer set was used for each of the included participants and thermal cycler conditions can be found in Supplementary Table 4.

The *env*-positive wells were collected and prepared for sequencing^6^. In brief, tagmentation was conducted using TDE1 Tagment DNA Enzyme (Illumina) after ligation of barcoded primers using KAPA HiFi HotStart ReadyMix (Roche). The amplicons were pooled to one library, purified and paired-end sequenced on a MiniSeq (Illumina), using MiniSeq Mid-Output Kit (Illumina).

### Rebound plasma HIV-1 *env* sequencing

Plasma rebound virus was sequenced for participants who received bNAbs and rebounded within the study period or were viremic at the end of the 25-week ATI. Extraction of RNA, cDNA synthesis and SGA of plasma HIV^6^, library preparation and sequencing were conducted as for proviral HIV-1 *env* sequencing.

### HIV-1 sequence assembly and annotation

Assembly and annotation of HIV-1 sequences was performed by The Rockefeller University pipeline (Defective and Intact HIV Genome Assembler)^6^. The pipeline implements quality control checks to remove PCR-amplified reads, correct errors, trim adaptors and low-quality bases and remove potential contaminant reads. Paired overlapping reads are merged and subsequently used to reconstruct HIV-1 contigs. The longest reconstructed HIV-1 contig is annotated by aligning it to HXB2 reference. Finally, sequences not classified as double peaks (cutoff consensus identity for any residue <70%) are classified as intact or defective. Samples with <500 sequencing reads and sequences with double peaks were omitted from downstream analysis. Only intact HIV-1 *env*s were considered in the subsequent analysis.

#### Sequence-based assessment of bNAb sensitivity

The HIV-1 *env* sequences were analyzed for sensitivity to 3BNC117 and 10-1074 by searching for resistance mutations using a model based on sequencing and neutralization data (Supplementary Table 1).

### Plasma antiretroviral drug concentrations

Antiretroviral drug concentrations in plasma were determined at BioXpedia using liquid chromatography with tandem mass spectrometry (LC–MS/MS) assay for individuals not fulfilling the criteria for viral rebound at week 13. Two timepoints were analyzed: (1) week 0 and (2) end of study, defined as the timepoint for reaching criteria for viral rebound or week 25, whichever came first.

### Serum bNAb concentrations

Each bNAb concentration in human serum was quantitatively measured using a sandwich immunoassay on Meso Scale Discovery Electrochemiluminescence (MSD-ECL) platform at PPD Bioanalytical Lab. In this assay, the bNAb was captured by biotinylated anti-idiotypic anti-bNAb antibody coated onto an MSD streptavidin plate, which was then detected by SULFO-TAG-conjugated anti-idiotypic anti-bNAb antibody. The plate was read on an MSD plate reader, resulting in assay signal proportional to the concentration of each bNAb. The method was validated following current regulatory guidance, and a sensitivity of 100 ng m^−1^ was established for each pharmacokinetic assay. We measured the serum bNAb concentrations at weeks 0, 6, 13 and 25 after ATI.

We used multiple imputation to impute missing values in serum bNAb concentrations. A detailed description can be found in Supplementary Table 2.

### Intact HIV-1 proviruses

The IPDA is used to measure intact and defective HIV-1 proviruses by targeting the Ψ and RRE, which are frequently deleted or mutated in defective proviruses^62^. We applied the IPDA protocol for all individuals regardless of subtype and found 20% (8/40) of the participants with Ψ, RRE or Ψ and RRE failure. For RRE failure, an alternative primer/probe set, reported to correlate with the original IPDA RRE primer/probe set, designed by Kinloch et al.^63^, was tested; this primer/probe set is located slightly downstream of the original IPDA RRE primer/probe set^63^. This alternative primer/probe set rescued all participants with RRE failure (*n* = 5) and worked for the participant with dual failure, thus leaving three participants with Ψ failure. For these three participants, the IPDA-like 3dPCR assay was applied, where individual primers and probes are designed for the participants based on sequencing of the participantsʼ virus^6^. The level of intact proviruses measured by IPDA and 3dPCR were previously shown to strongly correlate in individuals with HIV-1 subtype B; thus, 3dPCR provides a reasonable estimate of intact HIV-1 reservoir size, although it is possible that the assay’s positive predictive value for intact proviruses may, like the IPDA, vary between individuals^64^.

Regardless of the downstream assay applied—IPDA, IPDA with alternative RRE primer/probe or IPDA-like 3dPCR—CD4^+^ T cells (CD4^+^ T Cell Isolation Kit, Miltenyi Biotec) were isolated from 30 × 10^6^ PBMCs, and genomic DNA was extracted (DNeasy Blood and Tissue Kit, Qiagen). IPDA, IPDA with alternative RRE primer/probe and the IPDA-like 3dPCR were conducted^6,62,63^. An overview of the assay and primer/probe set used for each participant is provided in Supplementary Tables 3 and 4. In parallel with HIV-1 Ψ and RRE, all DNA extracts were also assayed in a separate duplexed ddPCR reaction targeting the human *RPP30* gene^63^; primers and probes are listed in Supplementary Table 5; and a detailed description of the method can be found in Supplementary Table 6.

### HIV-1-specific T cell immunity

HIV-1-specific T cell immunity was assessed using the AIM assay by flow cytometry at weeks 0, 6, 13 and 25 after ATI. Aliquots of cryopreserved PBMCs were thawed, washed and rested at 37 °C for 3 h. Cells were then plated into wells of a 96-well plate at a total of 1 × 10^6^ PBMCs per well in RMPI glutamine supplemented with penicillin–streptomycin and 10% FBS. Cells were then stimulated at a final concentration of 2 µg ml^−1^ of total peptide with four different HIV peptide pools for 20 h at 37 °C. Detailed description of the methods can be found in Supplementary Table 7, and the gating strategy can be found in Supplementary Fig. 1.

### Cytokine detection

Cytokine detection of IFN-γ and GzmB were measured in supernatants from Gag and non-stimulated AIM assay using MSD U-PLEX Custom Immuno-Oncology (K151AEM-2) according to the manufacturer’s instructions. The concentration of IFN-γ (pg ml^−1^) and GzmB (pg ml^−1^) in the supernatant of Gag-specific cells (AIM^+^ cells) was determined by subtracting the concentration of the non-stimulation condition from the stimulated condition.

### HLA class I typing

HLA class I (HLA-A, HLA-B and HLA-C) alleles were genotyped at the American Safety and Health Institute-accredited laboratory HistoGenetics using sequence-based typing.

### Outcomes

The primary endpoint was time to loss of virological control during ATI (sustained plasma HIV-1 RNA ≥1,000 copies per milliliter for 4 weeks or confirmed plasma HIV-1 RNA >100,000 copies per milliliter). Secondary endpoints were (1) safety, including CD4^+^ T cell counts, and (2) viral kinetics during ATI: time to plasma HIV-1 RNA >50 and >1,000 copies per milliliter as well as doubling time of the initial increase in plasma HIV-1 RNA. Exploratory endpoints were (1) changes in reservoir size measured by intact HIV-1 proviruses and (2) effects on HIV-1-specific T cell immunity using the AIM assay.

### Statistical methods

The analyses performed on primary, secondary and exploratory endpoints were pre-specified in the protocol. Paired two-tailed Wilcoxon tests and two-tailed Mann–Whitney tests were used to analyze non-parametric outcomes within and between groups, respectively. When more than two groups were compared, the Kruskal–Wallis test was used. Data are presented as median (IQR), median (range) or mean ± s.d. as indicated in each respective figure legend. The Kaplan–Meier estimator was used to assess the magnitude of the difference between the survival curves, and the log-rank test was used to compare time to loss of virologic control during ATI between groups. For correlations, Spearman’s correlation coefficient was used. We used the full analysis set, comprising all individuals receiving at least one dose of active treatment with assessable data, for the efficacy analyses and all enrolled individuals for the safety analyses. We used Stata version 17.0 and Prism version 7.0 software for statistical analyses.

### Reporting summary

Further information on research design is available in the Nature Portfolio Reporting Summary linked to this article.

## Online content

Any methods, additional references, Nature Portfolio reporting summaries, source data, extended data, supplementary information, acknowledgements, peer review information; details of author contributions and competing interests; and statements of data and code availability are available at 10.1038/s41591-023-02547-6.

### Supplementary information

Supplementary Information Supplementary Tables 1–7, Supplementary Fig. 1 and CONSORT 2010 checklist and study protocol: TITAN-001, version 3.0, 2 July 2021.

Reporting Summary

## Figures and Tables

**Extended Data Table 1:**
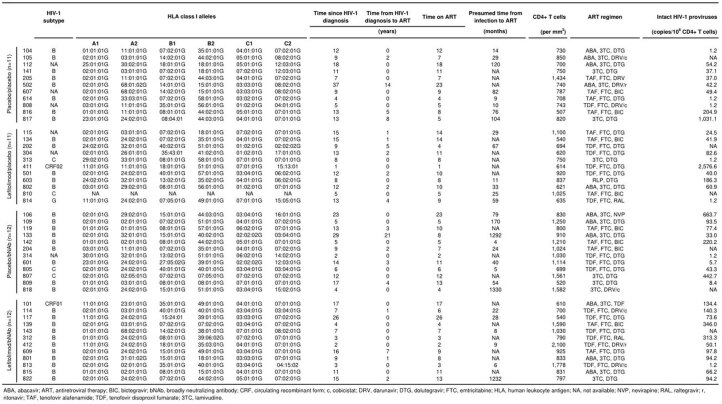
Individual characteristics of the study population at baseline

**Extended Data Table 2:**
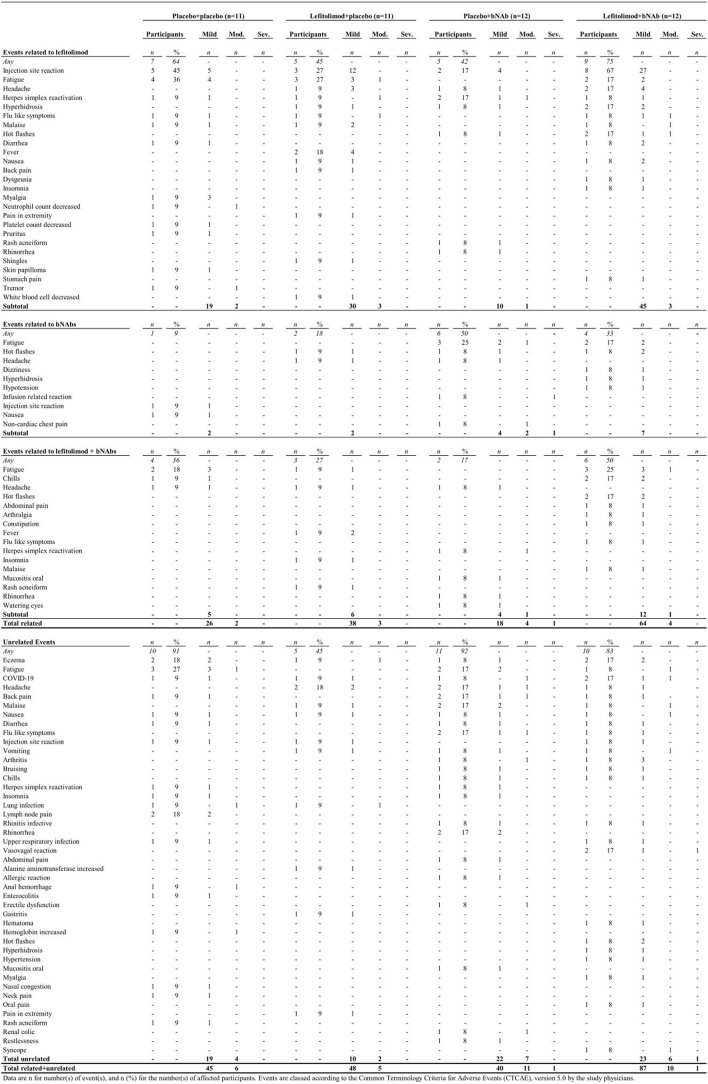
AEs by group

**Extended Data Table 3:**
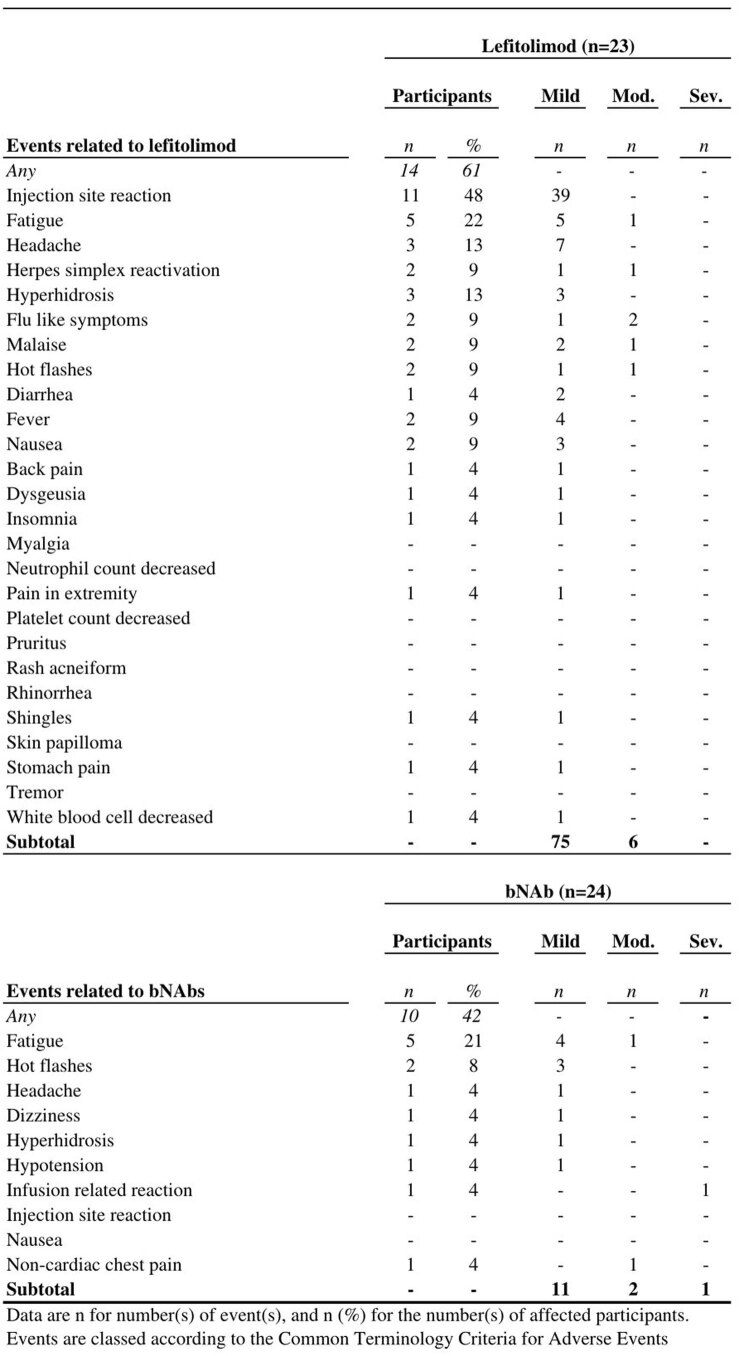
AEs by intervention

## Data Availability

Data are not available for download due to privacy/ethical restrictions under the European Union General Data Protection Regulation. Specific requests for access to the trial data may be sent to olesoega@rm.dk, and access may be provided to a named individual in agreement with the rules and regulations (https://www.datatilsynet.dk/english/legislation) of the Danish Data Protection agency and the Danish National Center for Ethics with a 2-week response timeframe for requests. All viral sequences have been deposited in GenBank with accession numbers OR014503 to OR015782 (www.ncbi.nlm.nih.gov/nucleotide/).
