# Supplementary material for: Impact of a TLR9 agonist and broadly neutralizing antibodies on HIV-1 persistence: the randomized phase 2a TITAN trial
Source: Nat Med. 2023 Sep 11;29(10):2547–58. doi: 10.1038/s41591-023-02547-6 (PMC10579101; doi:10.1038/s41591-023-02547-6)
Supplement: Supplementary file 1 — Supplementary Tables 1–7, Supplementary Fig. 1 and CONSORT 2010 checklist and study protocol: TITAN-001, version 3.0, 2 July 2021. [file 41591_2023_2547_MOESM1_ESM.pdf]

nature medicine

Article https://doi.org/10.1038/s41591-023-02547-6

Impact of a TLR9 agonist and broadly neutralizing antibodies on HIV-1 persistence: the randomized phase 2a TITAN trial

In the format provided by the authors and unedited

SUPPLEMENTARY MATERIALS

Impact of a TLR9 agonist and broadly neutralizing antibodies on HIV-1 persistence: the randomized phase 2a TITAN trial.

LIST OF SUPPLEMENTARY MATERIALS	PAGE
1. SUPPLEMENTARY TABLES AND FIGURE	2-11
2. CONSORT 2010 checklist of information to include when reporting a randomized trial	12-13
3. STUDY PROTOCOL: TITAN-001, version 3.0, 02 July 2021	14-

SUPPLEMENTARY TABLES AND FIGURE

TITLE	PAGE
**Supplementary Table S1.** Determination of bNAb-sensitive strains to 3BNC117 and 10-1074.	3
**Supplementary Table S2.** Detailed description to impute missing values in serum bNAb concentrations.	4
**Supplementary Table S3.** Assays used for quantification of intact and defective proviruses.	5
**Supplementary Table S4.** Primer set and combinations used for proviral HIV-1 *env* sequencing.	6-7
**Supplementary Table S5.** Primers and probes for targeting the human RPP30 gene.	8
**Supplementary Table S7.** Detailed description of the IPDA method.	9
**Supplementary Table S6.** Detailed description of surface marker antibodies used in the AIM assay.	10

TITLE	PAGE
**Supplementary Figure S1.** Gating strategy for the AIM assay.	11

Supplementary Table S1. Determination of bNAb-sensitive strains to 3BNC117 and 10-1074.

Based on published data, the following amino acid rules (using HXB2 numbering) were used to determine bNAb-sensitive strains:

**3BNC117:**	**10-1074:**
N or D at 279	D, N or T at 325
not K or D at 280	H or Y at 330
R at 456	N at 332
D at 457	Not P at 333
G at 458	Not S or T at 334
G at 459	
No PNGS at 279 (not N279 and S or T at 281)	

All viral sequences have been deposited in GenBank with accession numbers OR014503 to OR015782 (www.ncbi.nlm.nih.gov/nucleotide/).

Supplementary Table S2. Detailed description to impute missing values in serum bNAb concentrations.

Imputed missing values in serum bNAb concentrations could occur due to a measurement not being conducted or if the participant had reached criteria for viral rebound. After natural log transformation of the serum bNAb concentrations, we used linear regression in the imputation model for absent measurements. In brief, based on the observed values, the method estimates how the mean of natural log-transformed serum bNAb concentration depends on a linear function of covariates, assuming that the outcomes follow a normal distribution with an unknown but constant variance. This model is similar to ordinary linear regression, although observations may now be intervals instead of specific values. Once the mean function and variance were estimated, imputation values for the missing data were generated from a truncated normal distribution. To achieve good asymptotic properties of estimates, we imputed 100 datasets. The analysis of multiply imputed datasets was done via Rubin's formula, which states that analyses of each imputed dataset may be validly joined into a single combined estimate. Mixed-effects linear regression models with a fixed effect for individual participants were used to analyze decay rates between groups. The mean percentage decay rates per day were 18.4% (95% confidence interval [95% CI], 16.2%, 20.7%) for 3BNC117 and 18.5% (95% CI, 16.3%, 20.6%) for 10-1074 in the placebo/bNAb group, and 22.4% (95% CI, 16.7%, 27.6%) and 22.3% (95% CI, 17.0%, 27.3%), respectively, for the lefitolimod/bNAb group.

Supplementary Table S3. Assays used for quantification of intact and defective proviruses.

Intact Provirus DNA Assay (IDPA)
ID: 104, 106, 109, 112, 115, 119, 133, 134, 141, 304, 313, 411, 412, 501, 502, 601, 603, 607, 609, 614, 801, 802, 805, 807, 808, 809, 813, 814, 815, 816, 817 and 822 (n=32).

Envelope	RRE forward	RRE probe	anti-Hypermutant *env* Probe	RRE reverse
	AGTGGTGCAGAGAGAAAAAAGAGC	CCTTGGGTTCTTGGGA	CCTTAGGTTCTTAGGAGC	GTCTGGCCTGTACCGTCAGC

Psi	Ψ forward	Ψ probe	Ψ reverse
	CAGGACTCGGCTTGCTGAAG	TTTTGGCGTACTCACCAGT	GCACCCATCTCTCTCCTTCTAGC

IPDA – alternative RRE
ID: 101, 117, 139, 142 and 205 (n=5).

Envelope	RRE forward	RRE probe	RRE reverse
	ACTATGGGCGCAGCGTC	CTGGCCTGTACCGTCAG	CCCCAGACTGTGAGTTGCA

Psi (original IPDA)	Ψ forward	Ψ probe	Ψ reverse
	CAGGACTCGGCTTGCTGAAG	TTTTGGCGTACTCACCAGT	GCACCCATCTCTCTCCTTCTAGC

IPDA-like 3dPCR
ID: 114, 312 and 314 (n=3).

Envelope (original IPDA)	RRE forward	RRE probe	anti-Hypermutant *env* Probe	RRE reverse
	AGTGGTGCAGAGAGAAAAAAGAGC	CCTTGGGTTCTTGGGA	CCTTAGGTTCTTAGGAGC	GTCTGGCCTGTACCGTCAGC

Psi	Ψ forward	Ψ probe	Ψ reverse
Original oligo	CAGGACTCGGCTTGCTGAAG	TTTTGGCGTACTCACCAGT	GCACCCATCTCTCTCCTTCTAGC
114	A - - - - - - - - - - - - - - - - C T -	- - - - - - - - - - - - - - - - - - -	- -
143	- - - - - - - - - - - - - - - - - - - -	A - - - - - - - - - - - - - - - G T -	- -
312	- - - - - - - - - - - - - - - - - A - G C	- - - - - - - - - - - - - - - - - - -	- -
314*	- - - - - - - - - - - - - - - - - - - -	A A - - - - - - - - - - - - - - - - C	- -

*For this participant, the alternative env primers and probe was used.

Supplementary Table S4. Primer set and combinations used for proviral HIV-1 *env* sequencing.

Primer	Primer sequence	Thermal cycler conditions	ID
env B3 out[1]	5'- TTGCTACTTGTGATTGCTCCATGT -3'	94°C for 2 min., 35 cycles of 94°C for 15 sec., 58.5°C for 30 sec. and 68°C for 3 min and lastly 68°C for 15 min.	105, 117, 133, 141, 142, 143, 205, 411, 501, 502, 609, 813, 817, 822*
env B5 out[1]	5'- TAGAGCCCTGGAAGCATCCAGGAAG -3'		
env B3 in[1]	5'- GTCTCGAGATACTGCTCCCACCC -3'	94°C for 2 min., 45 cycles of 94°C for 15 sec., 61°C for 30 sec. and 68°C for 3 min. and lastly 68°C for 15 min.	
env B5 in[1]	5'- TTAGGCATCTCCTATGGCAGGAAGAAG -3'		

Primer	Primer sequence	Thermal cycler conditions	ID
B3F3[2]	5'- TGGAAAGGTGAAGGGGCAGTAGTAATAC -3'	94°C for 2 min., 45 cycles of 94°C for 15 sec., 55°C for 30 sec. and 68°C for 5 min and lastly 68°C for 15 min.	101, 104, 106, 109, 114, 119, 134, 139, 202, 204, 312, 412, 601, 801, 802, 809, 810, 818, 822*
R3B6R[2]	5'- TGAAGCACTCAAGGCAAGCTTTATTGAGGC -3'		
env B3 in[1]	5'- GTCTCGAGATACTGCTCCCACCC -3'	94°C for 2 min., 45 cycles of 94°C for 15 sec., 61°C for 30 sec. and 68°C for 3 min and lastly 68°C for 15 min.	
env B5 in[1]	5'- TTAGGCATCTCCTATGGCAGGAAGAAG -3'		

Primer	Primer sequence	Thermal cycler conditions	ID
805 B3 out[1]	5'- TTACTGGTTGTAAGTGCTCTATGT -3'	94°C for 2 min., 35 cycles of 94°C for 15 sec., 57°C for 30 sec. and 68°C for 3 min and lastly 68°C for 15 min.	805
805 B5 out[1]	5'- TAGAGCCCTAGAACCATCCAAGAAG -3'		
805 B3 in[1]	5'- GTCTTGAGATGCTGCTCTTACTT -3'	94°C for 2 min., 45 cycles of 94°C for 15 sec., 53°C for 30 sec. and 68°C for 3 min. and lastly 68°C for 15 min.	
805 B5 in[1]	5'- TTAGGCATTTGCTATGGCAGGAAGAAG -3'		

Primer	Primer sequence	Thermal cycler conditions	ID
B3F3[2]	5'- TGGAAAGGTGAAGGGGCAGTAGTAATAC -3'	94°C for 2 min., 45 cycles of 94°C for 15 sec., 55°C for 30 sec. and 68°C for 5 min and lastly 68°C for 15 min.	313, 807
R3B6R[2]	5'- TGAAGCACTCAAGGCAAGCTTTATTGAGGC -3'		
807 B3 in[1]	5'- GTCTCGAGACGCTGGTCCTACTC -3'	94°C for 2 min., 45 cycles of 94°C for 15 sec., 61°C for 30 sec. and 68°C for 3 min. and lastly 68°C for 15 min.	
env B5 in[1]	5'- TTAGGCATCTCCTATGGCAGGAAGAAG -3'		

Primer	Primer sequence	Thermal cycler conditions	ID
OFM19[3]	5'– GCACTCAAGGCAAGCTTTATTGAGGCTTA -3'	94°C for 2 min., 35 cycles of 94°C for 15 sec., 55°C for 30 sec. and 68°C for 4 min and lastly 68°C for 15 min.	815
Vif1[3]	5'– GGGTTTATTACAGGGACAGCAGAG -3'		
Env A[3]	5'– GGCTTAGGCATCTCCTATGGCAGGAAGAA -3'	94°C for 2 min., 45 cycles of 94°C for 15 sec., 55°C for 30 sec. and 68°C for 4 min. and lastly 68°C for 15 min.	
Env N[3]	5'– CTGCCAATCAGGGAAGTAGCCTTGTGT -3'		

Near-Full-Length[4]

Primer	Primer sequence	Thermal cycler conditions	ID
BLOuterF	5'– AAATCTCTAGCAGTGGCGCCCGAACAG -3'	94°C for 2 min, three cycles of 94°C for 30 s, 64°C for 30 s, and 68°C for 10 min, then three cycles of 94°C for 30 s, 61°C for 30 s, and 68°C for 10 min, then three cycles of 94°C for 30 s, 58°C for 30 s, and 68°C for 10 min, then 41 cycles of 94°C for 30 s, 55°C for 30 s, and 68°C for 10 min and lastly 68°C for 10 min.	805, 807
BLOuterR	5'– TGAGGGATCTCTAGTTACCAGAGTC -3'		
275F	5'– ACAGGGACCTGAAAGCGAAAG -3'	94°C for 2 min, three cycles of 94°C for 30 s, 64°C for 30 s, and 68°C for 10 min, then three cycles of 94°C for 30 s, 61°C for 30 s, and 68°C for 10 min, then three cycles of 94°C for 30 s, 58°C for 30 s, and 68°C for 10 min, then 41 cycles of 94°C for 30 s, 55°C for 30 s, and 68°C for 10 min and lastly 68°C for 10 min.	
280R	5'– CTAGTTACCAGAGTCACACAACAGACG -3'		

*ID822: Virus was amplified with two different primer set.

References

1. Wei, X. *et al.* Emergence of Resistant Human Immunodeficiency Virus Type 1 in Patients Receiving Fusion Inhibitor (T-20) Monotherapy. *Antimicrob. Agents Chemother.* **46**, 1896–1905 (2002).
2. Salazar-Gonzalez, J. F. *et al.* Genetic identity, biological phenotype, and evolutionary pathways of transmitted/founder viruses in acute and early HIV-1 infection. *J. Exp. Med.* **206**, 1273–1289 (2009).
3. Salazar-Gonzalez, J. F. *et al.* Deciphering Human Immunodeficiency Virus Type 1 Transmission and Early Envelope Diversification by Single-Genome Amplification and Sequencing. *J. Virol.* **82**, 3952–3970 (2008).
4. Ho, Y.-C. *et al.* Replication-Competent Noninduced Proviruses in the Latent Reservoir Increase Barrier to HIV-1 Cure. *Cell* **155**, 540–551 (2013).

Supplementary Table S5. Primers and probes for targeting the human RPP30 gene.

Primer/probe	Sequence
RPP30 Forward Primer	5'– GATTTGGACCTGCGAGCG -3'
RPP30 Probe	5'– VIC-CTGACCTGAAGGCTCT-MBBNFQ -3'
RPP30 Reverse Primer	5'– GCGGCTGTCTCCACAAGT -3'
RPP30-Shear Forward Primer	5'– CCATTTGCTGCTCCTTGGG -3'
RPP30- Shear Probe	5'– FAM-AAGGAGCAAGGTTCTATTGTAG-MGBNFQ -3'
RPP30 Shear Reverse Primer	5'– CATGCAAAGGAGGAAGCCG -3'

Supplementary Table S6. Detailed description of the method for IPDA.

HIV-1 and human *RPP30* copies were then normalized to the quantity of input DNA to estimate intact HIV-1 copies/10^6 CD4+ T-cells. Each ddPCR reaction contained genomic DNA, ddPCR Supermix for Probes (no dUTPs, BioRad), primers (final concentration 900nM, Integrated DNA Technologies), probes (final concentration 250nM, ThermoFisher Scientific), *XhoI* restriction enzyme and nuclease free water. Droplets were prepared using a QX200 Droplet Generator (BioRad) and cycled at 95°C for 10 min; 45 cycles of (94°C for 30 sec, 59°C for 1 min) and 98°C for 10 min, using a ramp rate of 2°C to improve droplet separation. Droplets were analyzed on a QX200 Droplet Reader (BioRad) using QuantaSoft software (BioRad, version 1.7.4), where replicate wells were merged prior to analysis. Intact HIV-1 copies (Ψ and *env* double-positive droplets) were corrected for DNA shearing based on the frequency of *RPP30* and *RPP30*-Shear double-positive droplets. The median (IQR) DNA shearing index, measuring the proportion of sheared DNA in a sample, was 0.34 (0.32–0.35), comparable to that reported in the original development of the IPDA. Frequencies of double-positive HIV-1 proviruses (intact), 5' and 3' defective HIV-1 proviruses were measured at week 0, 6, 13 and 25 after ATI.

Supplementary Table S7. Detailed description of the method for the AIM assay.

(HIV-Gag (JPT, PM-HIV-Gag), HIV-Env (JPT, PM-HIV-ENV), HIV-Nef (JPT, PM-HIV-Nef) and HIV-Pol (JPT, PM-HIV-Pol)). No exogenous stimulation with DMSO was used as negative control, and CEF (CMV, EBV and influenza virus) (JPT, PM-CEF-S-3) and staphylococcal enteroxin B (SEB, 1 µg/ml) were used as positive control. Following stimulation, cells were washed with Phosphate Buffered Saline (PBS) and stained for viability with Near IR Dead Live Dead for 20 minutes. After 10 minutes incubation with Human TruStain FcX (BioLegend) in PBS 2% FBS, cells were stained for 30 minutes with surface markers antibodies (see below). After washing, cells were acquired on a MACSQuant16. Analysis was performed using FlowJo 10.7.2. The frequency of antigen-specific cells (AIM+ cells) was determined by subtracting the frequency of the non-stimulation condition from the antigen stimulated conditions (Gag, Env, Nef and Pol).

Surface marker antibody	Details
CD3	PerCP/Cy5.5 anti-human CD3, SK7, BioLegend
CD4	BV650 anti-human CD4, RPA-T4, BioLegend
CD8	BV605 anti-human CD8a, RPA-T8, BioLegend
4-1BB	PE anti-human CD137, 4B4-1, BioLegend
CD69	APC anti-human CD69, FN50, BioLegend
PD-L1	BV421 anti-human CD274, B7-H1, BioLegend
OX40	FITC anti-human CD134, Ber-ACT35 (ACT35), BioLegend

Supplementary Figure S1. Gating strategy for the AIM assay.

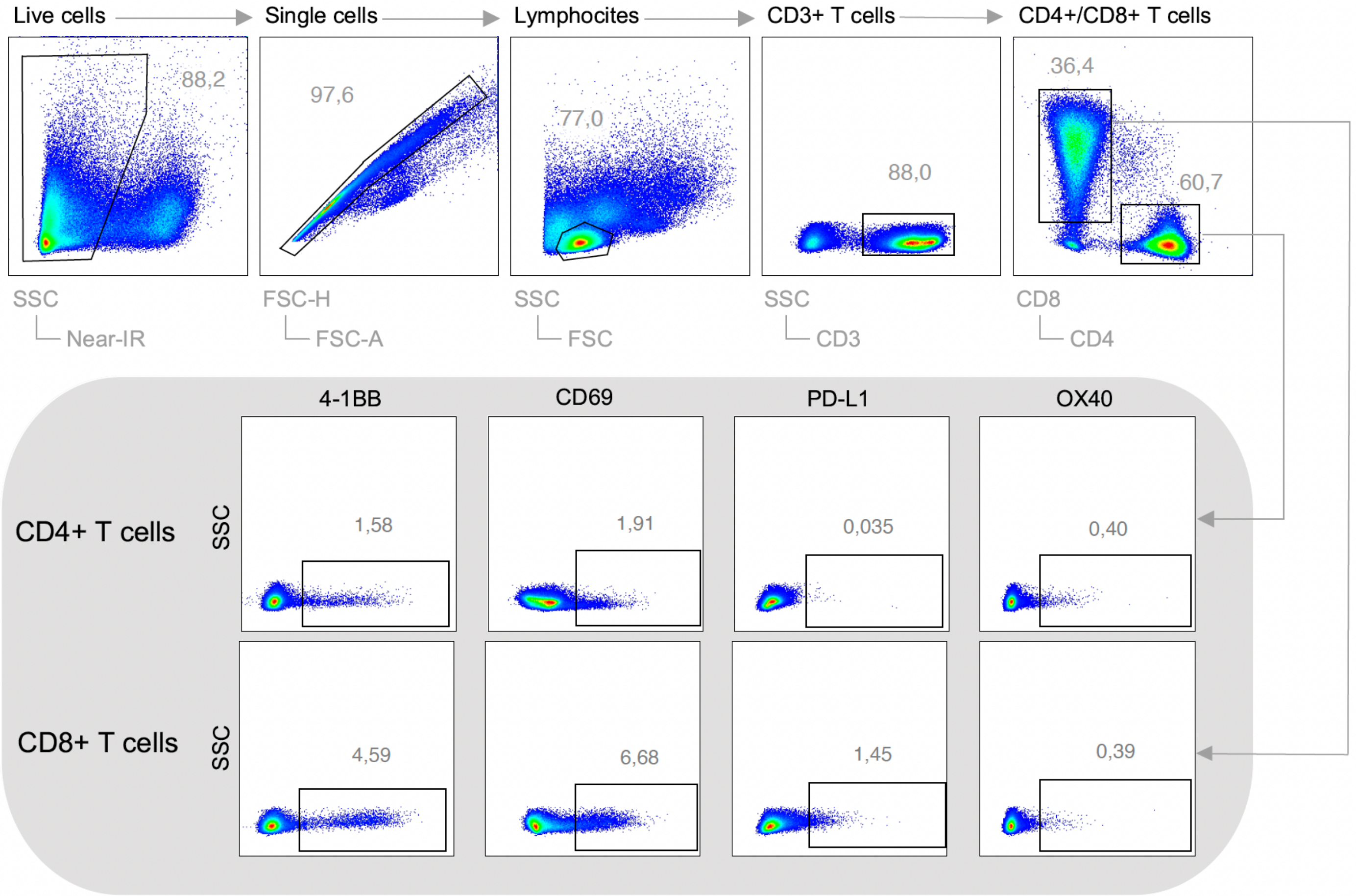

Gag-specific AIM+ cells were considered as the addition of the frequency of cells that were either CD69+PD-L1+4-1BB+OX40+, CD69+PD-L1+OX40+, 4-1BB+OX40+PD-L1+, CD69+PD-L1+4-1BB+, OX40+CD69+4-1BB+, CD69+PD-L1+, CD69+4-1BB+, OX40+PD-L1+, CD69+OX40+, 4-1BB+OX40+ or PD-L1+4-1BB+. Total HIV-specific AIM+ cells was calculated as summation of each of the 11 populations for the four antigen-stimulations.

CONSORT 2010 checklist of information to include when reporting a randomised trial*

Section/Topic	Item No	Checklist item	Reported on page No
**Title and abstract**			
	1a	Identification as a randomised trial in the title	Title
	1b	Structured summary of trial design, methods, results, and conclusions (for specific guidance see CONSORT for abstracts)	Abstract
**Introduction**			
Background and objectives	2a	Scientific background and explanation of rationale	Main 4-5
	2b	Specific objectives or hypotheses	Main 5
**Methods**			
Trial design	3a	Description of trial design (such as parallel, factorial) including allocation ratio	Main 21
	3b	Important changes to methods after trial commencement (such as eligibility criteria), with reasons	NA
Participants	4a	Eligibility criteria for participants	Main 21-22 + Supplement
	4b	Settings and locations where the data were collected	Main 21
Interventions	5	The interventions for each group with sufficient details to allow replication, including how and when they were actually administered	Main 21
Outcomes	6a	Completely defined pre-specified primary and secondary outcome measures, including how and when they were assessed	Main 22-28
	6b	Any changes to trial outcomes after the trial commenced, with reasons	Main 29
Sample size	7a	How sample size was determined	Main 29
	7b	When applicable, explanation of any interim analyses and stopping guidelines	NA
Randomisation:			
Sequence generation	8a	Method used to generate the random allocation sequence	Main 22
	8b	Type of randomisation; details of any restriction (such as blocking and block size)	Main 22
Allocation concealment mechanism	9	Mechanism used to implement the random allocation sequence (such as sequentially numbered containers), describing any steps taken to conceal the sequence until interventions were assigned	Main 22
Implementation	10	Who generated the random allocation sequence, who enrolled participants, and who assigned participants to interventions	Main 22
Blinding	11a	If done, who was blinded after assignment to interventions (for example, participants, care providers, those assessing outcomes) and how	Main 22

	11b	If relevant, description of the similarity of interventions	Main 22
Statistical methods	12a	Statistical methods used to compare groups for primary and secondary outcomes	Suppl 10-11
	12b	Methods for additional analyses, such as subgroup analyses and adjusted analyses	NA
**Results**			
Participant flow (a diagram is strongly recommended)	13a	For each group, the numbers of participants who were randomly assigned, received intended treatment, and were analysed for the primary outcome	Main 6
	13b	For each group, losses and exclusions after randomisation, together with reasons	Main 6
Recruitment	14a	Dates defining the periods of recruitment and follow-up	Main 6
	14b	Why the trial ended or was stopped	Main 6
Baseline data	15	A table showing baseline demographic and clinical characteristics for each group	Tab 1
Numbers analysed	16	For each group, number of participants (denominator) included in each analysis and whether the analysis was by original assigned groups	Ext Fig. 1
Outcomes and estimation	17a	For each primary and secondary outcome, results for each group, and the estimated effect size and its precision (such as 95% confidence interval)	Main 6-7
	17b	For binary outcomes, presentation of both absolute and relative effect sizes is recommended	NA
Ancillary analyses	18	Results of any other analyses performed, including subgroup analyses and adjusted analyses, distinguishing pre-specified from exploratory	Main 7-10
Harms	19	All important harms or unintended effects in each group (for specific guidance see CONSORT for harms)	Main 10
**Discussion**			
Limitations	20	Trial limitations, addressing sources of potential bias, imprecision, and, if relevant, multiplicity of analyses	Main 13-14
Generalisability	21	Generalisability (external validity, applicability) of the trial findings	Main 13-14
Interpretation	22	Interpretation consistent with results, balancing benefits and harms, and considering other relevant evidence	Main 11-14
**Other information**			
Registration	23	Registration number and name of trial registry	Main 1+3
Protocol	24	Where the full trial protocol can be accessed, if available	Supplement
Funding	25	Sources of funding and other support (such as supply of drugs), role of funders	3+15

*We strongly recommend reading this statement in conjunction with the CONSORT 2010 Explanation and Elaboration for important clarifications on all the items. If relevant, we also recommend reading CONSORT extensions for cluster randomised trials, non-inferiority and equivalence trials, non-pharmacological treatments, herbal interventions, and pragmatic trials. Additional extensions are forthcoming: for those and for up to date references relevant to this checklist, see www.consort-statement.org.

Confidential

Clinical Trial Protocol

Combining a TLR9 agonist with broadly neutralizing antibodies for reservoir reduction and immunological control of HIV infection: An investigator-initiated randomized, placebo-controlled, phase IIa trial

(TITAN)

Trial Identification: TITAN-001
EudraCT No: 2018-001165-16

Version: 3.0 - DK
Date: 02 July 2021

This Study Protocol is the property of Aarhus University Hospital and is a confidential document. It is not to be copied or distributed to other parties without written approval from Aarhus University Hospital.

1 **Responsibilities and Partnerships** ... 6
1.1 Sponsor ... 6
1.2 EudraCT number ... 6
1.3 Lead Principal Investigator ... 6
1.4 Site Principal Investigators ... 6
1.5 Coordinating Investigator ... 7
1.6 Investigators ... 7
1.7 Collaborators ... 7
1.8 Study monitor ... 7
1.9 Guidelines ... 7
1.10 Time plan ... 8
1.11 Signature Page 1 ... 9
1.12 Signature Page 2 ... 10
2 **Introduction** ... 12
2.1 Background and Rationale ... 12
2.1.1 Overview of the Investigational Medicinal Products (IMPs) ... 14
2.1.1.1 Lefitolimod (MGN1703) ... 14
2.1.1.2 3BNC117 and 10-1074 ... 15
2.1.2 Clinical Experience ... 15
2.1.2.1 Lefitolimod (MGN1703) ... 15
2.1.2.2 3BNC117 ... 17
2.1.2.3 10-1074 ... 18
2.1.3 Pharmacokinetics (PK) ... 20
2.1.3.1 Lefitolimod (MGN1703) ... 20
2.1.3.2 3BNC117 ... 21
2.1.3.3 10-1074 ... 21
2.1.4 Interactions with other drugs: ... 21
2.1.4.1 Lefitolimod (MGN1703) ... 21
2.1.4.2 3BNC117 and 10-1074 ... 22
2.1.5 Safety Profile for each investigational medical product ... 23
2.1.5.1 Lefitolimod (MGN1703) ... 23
2.1.5.2 Safety information related to antibody infusions ... 25
2.1.5.3 Safety data directly related to 3BNC117 ... 26
2.1.5.4 Safety data directly related to 10-1074 ... 28
2.1.6 Rationale for dose selection ... 29
2.1.6.1 Lefitolimod (MGN1703) ... 29
2.1.6.2 3BNC117 ... 30
2.1.6.3 10-1074 ... 30
3 **Study Objectives and Endpoints** ... 30
3.1 Objectives ... 31
3.1.1 Primary Objective ... 31
3.1.2 Secondary Objective ... 31
3.1.3 Exploratory Objective ... 31
3.2 Endpoints ... 31
3.2.1 Primary Endpoint ... 31
3.2.2 Secondary Endpoints ... 31

3.2.3 Potential Exploratory Analyses ... 31
4 Study Design and General Procedures ... 32
4.1 Design ... 32
4.2 Health Facilities ... 32
4.3 Randomization ... 33
4.4 Discontinuation of Study ... 33
4.5 Source Data ... 33
5 Selection of Study Subjects ... 34
5.1 Inclusion Criteria ... 34
5.2 Exclusion Criteria ... 35
5.3 Subject Withdrawal from Study ... 35
5.4 Subject Withdrawal from the Investigational Medicinal Product(s) (IMPs) ... 36
6 Study Treatment ... 36
6.1 The Investigational Medicinal Products (IMPs) ... 36
6.2 Dosing Modifications ... 36
6.2.1 Lefitolimod (MGN1703) ... 36
6.2.2 3BNC117 ... 36
6.2.3 10-1074 ... 36
6.2.4 Placebo - sterile physiological saline ... 36
6.3 Consent Procedure ... 36
6.4 Drug Supplies, Packaging and Labelling ... 37
6.4.1 Lefitolimod (MGN1703, Mologen AG for Gilead Sciences Inc.) ... 37
6.4.2 3BNC117 ... 37
6.4.3 10-1074 ... 37
6.4.4 Placebo - sterile physiological saline ... 38
6.5 Drug Storage and Accountability ... 38
6.6 Drug Preparation and Administration ... 38
6.6.1 Lefitolimod ... 39
6.6.2 3BNC117 ... 39
6.6.3 10-1074 ... 39
6.6.4 Placebo - sterile physiological saline ... 39
6.7 Concomitant Medication(s) ... 39
7 Study Overview ... 39
7.1 Study visits (Outlined in Table 4) ... 39
7.1.1 Visit 1/Day -140 to -15; screening ... 40
7.1.2 Visit 2/Day 0. Baseline. Initiation of interventions ... 40
7.1.3 Visit 3/Day 7 (+/- 3 day) ... 40
7.1.4 Visit 4/Day 14 (+/- 3 day) ... 40
7.2 The analytical treatment interruption ... 41
7.2.1 Visit 5/Day 21 (+/- 3 day) ... 41
7.2.2 Visit 6/Day 28 (+/- 3 day) ... 41
7.2.3 Visit 7/Day 35 (+/- 3 day) ... 41
7.2.4 Visit 8/Day 42 (+/- 3 day) ... 41
7.2.5 Visit 9/Day 49 (+/- 3 day) ... 41
7.2.6 Visit 10a/Day 56 (+/- 3 days) ... 41
7.2.7 Visit 10b/Day 63 (+/- 3 days) ... 42

7.2.8 Visit 10c/Day 77 (+/- 3 days) ... 42
7.2.9 Visit 10d/Day 91 (+/- 3 days) ... 42
7.2.10 Visit 10e/Day 105 (+/- 3 days) ... 42
7.2.11 Visit 10f/Day 119 (+/- 3 days) ... 42
7.2.12 Visit 10g/Day 133 (+/- 3 days) ... 42
7.2.13 Visit 10h/Day 147 (+/- 3 days) ... 42
7.2.14 Visit 10i/Day 161 (+/- 3 days) ... 43
7.2.15 Visit 10j/Day 175 (+/- 3 days) ... 43
7.3 Resumption of ART during ATI: ... 43
7.3.1 Visit 11/Day 189 (+/- 3 days) or day of resumption of ART during ATI ... 43
7.4 Re-suppression following end of ATI ... 43
7.4.1 Visit 12, 4-8 weeks following resuppresion of viremia (as defined in 7.3.1) ... 43
7.5 End of trial ... 44
7.6 Blood sampling ... 44
7.6.1 Blood volume per analyses ... 44
7.6.2 Total amount of blood per visit ... 44
7.7 Stool sampling (optional) ... 44
7.7.1 Stool sample analysis ... 45
7.8 Safety monitoring ... 45
7.8.1 Analytical ART Interruption (starting on Day 15) ... 47
7.8.2 Lefitolimod ... 47
7.8.3 3BNC117 ... 49
7.8.4 10-1074 ... 49
7.9 Unscheduled Visits or Telephone Contacts ... 50
7.9.1.1 Withdrawal of ART ... 50
8 Efficacy Assessments ... 50
8.1 Primary Endpoint ... 50
8.1.1 Analytical Treatment Interruption ... 50
8.2 Secondary Endpoints ... 50
8.2.1 Safety and Tolerability assessment ... 50
8.2.2 Plasma HIV-1 RNA ... 50
8.2.3 Potential Exploratory Analyses ... 50
9 Safety Assessments ... 51
9.1 Protocol Safety Review Team ... 51
9.2 Safety Monitoring Committee (SMC) ... 51
9.3 Monitoring ... 52
9.4 Procedure for premature unblinding ... 52
9.5 Adverse Events (AE) ... 52
9.6 Serious Adverse Events (SAE) ... 53
9.6.1 Pregnancies ... 53
9.7 Recording of Adverse Events (AE) ... 53
9.8 Reporting of Serious Adverse Events (SAE) ... 53
9.9.1 Reporting of SUSAR to local Health and Medicines Authority and the Ethics Committee ... 54
9.10 Submission of Development Safety Update Report (DSUR) ... 55
10 Statistical Analyses ... 55
10.1 Sample Size Determination ... 55

10.2 General Data Analyses 55
11 Access to Source Data 56
12 Study Monitoring and Quality Assurance 56
12.1 Sponsor and Investigator's Responsibility 56
12.2 Study Monitoring 56
12.3 Use of Case Report Forms (CRF) 57
13 Ethical Considerations 57
13.1 Study Approvals 57
13.2 Ethical Conduct of the Study 57
13.3 Subject Recruitment, Information and Consent 57
13.4 Harms, Risks and Inconveniences 57
13.5 Evaluation of Ethical and Scientific Aspects 58
14 Data Handling and Record Keeping 59
14.1 Access to Personal Information 59
14.2 Handling of Personal Information 59
14.3 Research Biobank 60
15 Financial Issues and Insurance 60
16 Publication of Study 60
16.1 Public Registration of Study 60
16.2 Publication of Study Results 60
17 Strategy for literature review 61
18 Protocol Synopsis 61
18.1 Title 61
18.2 Background and study rationale 61
18.3 Objectives 62
18.3.1 Primary Objective 62
18.3.2 Secondary Objective 62
18.3.3 Exploratory Objective 62
18.4 Endpoints 62
18.4.1 Primary Endpoint 62
18.4.2 Secondary Endpoints 62
18.4.3 Potential Exploratory Analyses 62
19 List of abbreviations 62
20 List of figures and Tables 64
21 References 64

1 RESPONSIBILITIES AND PARTNERSHIPS

1.1 Sponsor

Lars Østergaard, MD, DMSc, PhD, Head of Department/Professor
Department of Infectious Diseases, Aarhus University Hospital, Denmark
Palle Juul-Jensens Boulevard 99, DK-8200 Aarhus N
Telephone: +45 78 45 28 00, email: larsoest@rm.dk

1.2 EudraCT number

2018-001165-16

1.3 Lead Principal Investigator

Ole Schmeltz Søgaard, MD, PhD, Associate professor
Department of Infectious Diseases, Aarhus University Hospital
Palle Juul-Jensens Boulevard 99, DK-8200 Aarhus N, Denmark
Telephone: +45 78 45 28 42, email: olesoega@rm.dk

1.4 Site Principal Investigators

Ole Schmeltz Søgaard, MD, PhD, Associate professor
Department of Infectious Diseases, Aarhus University Hospital
Palle Juul-Jensens Boulevard 99, DK-8200 Aarhus N, Denmark
Telephone: +45 78 45 28 42, email: olesoega@rm.dk

Sharon Lewin, MD, PhD, Professor
Director - The Peter Doherty Institute for Infection and Immunity
792 Elizabeth Street, Melbourne, Australia 3000
Telephone: (03) 8344 3159, email: Sharon.lewin@unimelb.edu.au

Adam M. Spivak, MD, Assistant Professor
Department of Internal Medicine, University of Utah
30 North 1900 East RM 4B319, Salt Lake City, Utah 84132
Telephone: +1 801-581-8812, email: adam.spivak@hsc.utah.edu

Henrik Nielsen, MD, Professor
Department of Infectious Diseases, Aalborg University Hospital
Mølleparkvej 4, 9000 Aalborg, Denmark
Telephone: +45 97663930, email: henrik.nielsen@rn.dk

Isik Johansen, MD, Professor
Department of Infectious Diseases, Odense University Hospital
Sdr. Boulevard 29, 5000 Odense C, Denmark
Telephone: +45 65412652, email: isik.somuncu.johansen@rsyd.dk

Thomas Benfield, MD, Professor
Department of Infectious Diseases, Amager and Hvidovre Hospitals
Kettegård Allé 30, 2650 Hvidovre, Denmark
Telephone: +45 38622302, email: Thomas.lars.benfield@regionh.dk

Jan Gerstoft, MD, Professor
Department of Infectious Diseases, Rigshospitalet
Blegdamsvej 9, 2100 Copenhagen, Denmark
Telephone: +45 35457744, email: jan.gerstoft@regionh.dk

Dag Henrik Reikvam, MD, PhD
Department of Infectious Diseases, Oslo University Hospital
Kirkeveien 166 (Ullevaal Hospital), 0450 Oslo, Norway
Telephone: +47 22119881, email: dagrei@ous-hf.no

1.5 Coordinating Investigator
Jesper Damsgaard Gunst, MD, Ph.D.,
Department of Infectious Diseases, Aarhus University Hospital
Palle Juul-Jensens Boulevard 99, DK-8200 Aarhus N, Denmark
Telephone: +45 23 88 66 36, mail: jesdam@rm.dk

1.6 Investigators
Thomas Aagaard Rasmussen, MD, Ph.D.,
Department of Infectious Diseases
Alfred Hospital and Monash University, Melbourne, Australia

Martin Tolstrup, Ph.D., MSc.,
Department of Infectious Diseases, Aarhus University Hospital
Palle Juul-Jensens Boulevard 99, DK-8200 Aarhus N, Denmark

Paul W. Denton, Ph.D., MSc.,
Department of Infectious Diseases, Aarhus University Hospital
Palle Juul-Jensens Boulevard 99, DK-8200 Aarhus N, Denmark

James McMahon MBBS MPH PhD
Department of Infectious Diseases
Alfred Hospital and Monash University, Melbourne, Australia

1.7 Collaborators
Michel Nussenzweig, MD, Ph.D., Professor
The Rockefeller University
1230 York Avenue, New York 10065, USA

Steven Deeks, MD, Professor
UCSF School of Medicine
1001 Potrero Ave, San Francisco, CA 94110
Telephone: +1 415-476-4082, email: Steven.Deeks@ucsf.edu

Department of Clinical Biochemistry, Aarhus University Hospital
Palle Juul-Jensens Boulevard 99, DK-8200 Aarhus N, Denmark

Hospital Pharmacy at Aarhus University Hospital
Nørrebrogade 44, DK-8000 Aarhus C, Denmark

1.8 Study monitor
Good Clinical Practice (GCP) unit at Aalborg and Aarhus University Hospitals
Aarhus University, Olof Palmes Allé 15
8200 Aarhus N, Denmark
Telephone: +45 784 13950
http://clin.au.dk/gcp-enheden/

1.9 Guidelines
This study will be conducted in accordance with the protocol, The Helsinki Declaration (1996 version), The International Conference on Harmonization guidelines for GCP and national ethical guidelines and law.

1.10 Time plan

We plan to enroll study participants between the fourth quarter of 2018 and first quarter of 2022. The last planned visit date will be in third quarter of 2022. Expected main study report by the third quarter 2022. Follow-up of patients who remain off antiretroviral treatment after official end-of-study visit may continue until 2025.

1.11 Signature Page 1

The signature below constitutes the approval of this protocol and the attachments, and provides the necessary assurances that this trial will be conducted according to all stipulations of the protocol, including all statements regarding confidentiality, and according to local legal and regulatory requirements and applicable European Union regulations and The International Conference on Harmonization guidelines.

The Sponsor, Lead Principal Investigator and Coordinating Investigator should sign Signature Page 1. A copy of this Signature Page 1 should be filed with the holder of the Regulatory documents and a copy should be maintained at each site.

Sponsor:
Lars Østergaard, Head of Department/Professor, DMSc, Ph.D., MD,
Department of Infectious Diseases, Aarhus University Hospital
Palle Juul-Jensens Boulevard 99, DK-8200 Aarhus N, Denmark

Date and signature: ____

Lead Principal Investigator:
Ole Schmeltz Søgaard, Associate professor, MD, Ph.D.
Department of Infectious Diseases, Aarhus University Hospital
Palle Juul-Jensens Boulevard 99, DK-8200 Aarhus N, Denmark

Date and signature: ____

Coordinating Investigator:
Jesper Falkesgaard Højen, MD, Ph.D.
Department of Infectious Diseases, Aarhus University Hospital
Palle Juul-Jensens Boulevard 99, DK-8200 Aarhus N, Denmark

Date and signature: ____

1.12 Signature Page 2

The signature below constitutes the approval of this protocol and the attachments, and provides the necessary assurances that this trial will be conducted according to all stipulations of the protocol, including all statements regarding confidentiality, and according to local legal and regulatory requirements and applicable European Union regulations and The International Conference on Harmonization guidelines.

The Principal Investigator from each participating clinical site should sign the Signature Page 2 as appropriate. This Signature Page 2 should be maintained at each site.

Principal Investigator (Aarhus):
Ole Schmeltz Søgaard, Associate Professor, MD, Ph.D.
Department of Infectious Diseases, Aarhus University Hospital
Palle Juul-Jensens Boulevard 99, DK-8200 Aarhus N, Denmark
Telephone: +45 78 45 28 42, email: olesoega@rm.dk

Date and signature: ____

Principal Investigator (Melbourne):
Sharon Lewin, MD, PhD, Professor
Director - The Peter Doherty Institute for Infection and Immunity
792 Elizabeth Street, Melbourne, Australia 3000
Telephone: (03) 8344 3159, email: Sharon.lewin@unimelb.edu.au

Date and signature: ____

Principal Investigator (Salt Lake city)
Adam M. Spivak, MD, Assistant Professor
Department of Internal Medicine, University of Utah
30 North 1900 East RM 4B319, Salt Lake City, Utah 84132
Telephone: +1 801-581-8812, email: adam.spivak@hsc.utah.edu

Date and signature: ____

Principal Investigator (Ålborg):
Henrik Nielsen, MD, Professor
Department of Infectious Diseases, Aalborg University Hospital
Mølleparkvej 4, 9000 Aalborg, Denmark
Telephone: +45 97663930, email: henrik.nielsen@rn.dk

Date and signature: ____

Principal Investigator (Odense):
Isik Johansen, MD, Professor
Department of Infectious Diseases, Odense University Hospital
Sdr. Boulevard 29, 5000 Odense C, Denmark
Telephone: +45 65412652, email: isik.somuncu.johansen@rsyd.dk

Date and signature: ____

Principal Investigator (Hvidovre):
Thomas Benfield, MD, Professor
Department of Infectious Diseases, Amager and Hvidovre Hospitals
Kettegård Allé 30, 2650 Hvidovre, Denmark
Telephone: +45 38622302, email: Thomas.lars.benfield@regionh.dk

Date and signature: ____

Principal Investigator (København):
Jan Gerstoft, MD, Professor
Department of Infectious Diseases, Rigshospitalet
Blegdamsvej 9, 2100 Copenhagen, Denmark
Telephone: +45 35457744, email: jan.gerstoft@regionh.dk

Date and signature: ____

Principal Investigator (Oslo):
Dag Henrik Reikvam, MD, PhD
Department of Infectious Diseases, Oslo University Hospital
Kirkeveien 166 (Ullevaal Hospital), 0450 Oslo, Norway
Telephone: +47 22119881, email: dagrei@ous-hf.no

Date and signature: ____

2 INTRODUCTION

2.1 Background and Rationale

Reservoirs of HIV-1 persists in the body despite effective combination antiretroviral therapy (ART) and upon treatment interruption, the virus quickly replicates such that viremia rebounds to pre-treatment levels (2). The primary barrier preventing eradication of HIV-infection by ART is a pool of long-lived persistently (i.e. latently) infected memory CD4+ T-cells (3, 4). In the inactive, resting state these cells are unrecognizable to the immune system and the virus is unresponsive to antiretroviral drugs. These cells harbor integrated proviral DNA capable of resuming HIV-expression upon subsequent activation (5-7). The immune system can target infected cells during active viral replication, but augmentation of antiviral immune responses could enhance the killing effect leading to a better clearance of the infection.

Although the size of the persistent viral reservoir decreases following initiation of ART, the decay rate is so slow that all individuals regardless of the duration of ART harbor thousands to millions of infected CD4+ T cells containing replication competent proviruses. Recent studies demonstrate the complexity of finding a biomarker that accurately predicts viral rebound, although the size of the HIV-1 reservoir appears to be the most important predictor of time from interrupting ART to viral rebound (8, 9). Thus, it has been proposed that reducing the size of the reservoir will increase the chances of achieving ART-free HIV remission. Proof-of-concept trials testing the "shock and kill" approach to eradicate this HIV-1 reservoir (10) have demonstrated that persistently infected cells can be impacted to produce virus, as evidenced by enhanced transcription of HIV-1 RNA following treatment with latency reversing agents (11-17). However, these clinical trials have found no, or only modest, reductions in the size of the HIV-1 reservoir, possibly due to insufficient immune-mediated killing of the persistently infected cells (18). *In vivo* and *ex vivo* studies have demonstrated that priming of specific effector cells, such as cytotoxic T lymphocytes (CTLs) and natural killer (NK) cells, enhance their ability to recognize and eliminate infected cells (18-22). This indicates that therapeutic interventions that boost NK and CTL-mediated cellular immunity in humans may lead to the elimination of cells expressing HIV-1 antigens. In addition to cellular immune responses, broadly neutralizing antibodies (bNAbs) against conserved viral proteins facilitate clearance of cells expressing HIV-1 envelope through FcγR-dependent mechanisms (23). Thus, there is a compelling rationale that combining the qualities of enhanced cellular immunity with potent and broad HIV-specific humoral immunity will lead to effective killing of cells expressing HIV antigens, which in the presence of a latency reversing agent (e.g. histone deacetylase inhibitor or toll-like receptor [TLR] agonist) may result in a significant reduction in the frequency of persistently infected cells.

However, recent studies have shown that even large reductions in the size of the viral reservoir may not be sufficient to induce long-term ART free HIV-1 remission (8, 24-26). Among individuals with undetectable HIV DNA using very large numbers of cells from both blood and tissue, the infection reappears weeks or months after stopping ART suggesting persistent and potent immune surveillance mechanism is needed in addition to reduce the frequency of persistently infected cells to achieve long-term remission.

To tackle the challenge of improving immunity to drastically reduce the size of the persistent HIV reservoir, we propose to combine three immunotherapeutic interventions, a TLR9 agonist and two bNAbs against HIV-1 envelope in a randomized clinical trial. As outlined below, this treatment combination may not only have an immediate effect on the frequency of persistently infected cells, but may also lead to the development of long-lasting immune surveillance mechanisms. While this treatment concept is new is the HIV field, cancer studies have provided proof-of-concept that the effects of TLR9 agonists can synergize with the effects of monoclonal antibodies such as rituximab and cetuximab leading to increased elimination of target cells via FcγR dependent mechanisms (27).

Lefitolimod (MGN1703) is a dumbbell-shaped DNA molecule that stimulates TLR9 in human plasmacytoid dendritic cells (pDCs) and B cells (28). Upon TLR9 stimulation, activated pDCs release type I interferons and migrate to lymphoid tissue where they facilitate B cell maturation, germinal center formation, enhanced cross-priming and CD8 T cell immunity. Another unique feature of TLR9 agonists is their ability to activate NK cells (Figure 1), neutrophils, monocytes and macrophages leading to potent induction of antibody-dependent effector mechanisms such as cellular cytotoxicity (ADCC) (29). Lefitolimod belongs to a class of drugs referred to as immune surveillance reactivators and it is currently in phase III testing for treatment of metastatic colorectal cancer (30). Most synthetic TLR9 agonists, CpG-oligodeoxynucleotides, require

chemical stabilization by a phosphorothioate backbone. This backbone structure confers reactogenic potential resulting in dose-limiting side effects in humans (31, 32). Unlike, CpG-oligonucleotide-based TLR9 agonists, lefitolimod is a double-stranded DNA molecule without extraneous modifications. The closed structure of lefitolimod prevents rapid nuclease-mediated degradation *in vivo*. We recently demonstrated that lefitolimod enhanced the cellular antiviral immune response and increased cell associated unspliced HIV RNA in peripheral blood mononuclear cells (PBMCs) isolated from HIV infected individuals on ART (33). These pre-clinical findings indicated that lefitolimod might confer a dual effect in HIV-1 eradication by reversing latency and enhancing antiviral immunity.

To test the effect of lefitolimod on antiviral immunity and the HIV-1 reservoir *in vivo*, we conducted a trial in which 15 virologically suppressed HIV-1 infected individuals on antiretroviral therapy received 60 mg lefitolimod subcutanously twice weekly for 4 weeks (1). Shortly after administration, we observed pronounced activation of pDCs and elevated levels of plasma interferon-α2 ($p<0.0001$). Subsequently, proportions of activated cytotoxic NK cells and $CD8^+$ T cells expanded significantly. In 6 of 15 participants, plasma HIV-1 RNA blips from 21 copies/mL up to >1500 copies/mL were observed during lefitolimod treatment (Figure 1). Consistent with our hypothesis, these data indicate that TLR9 stimulation with lefitolimod reactivated latent HIV-1 and enhanced antiviral immunity: both of which are key outcomes in HIV-1 eradication therapy.

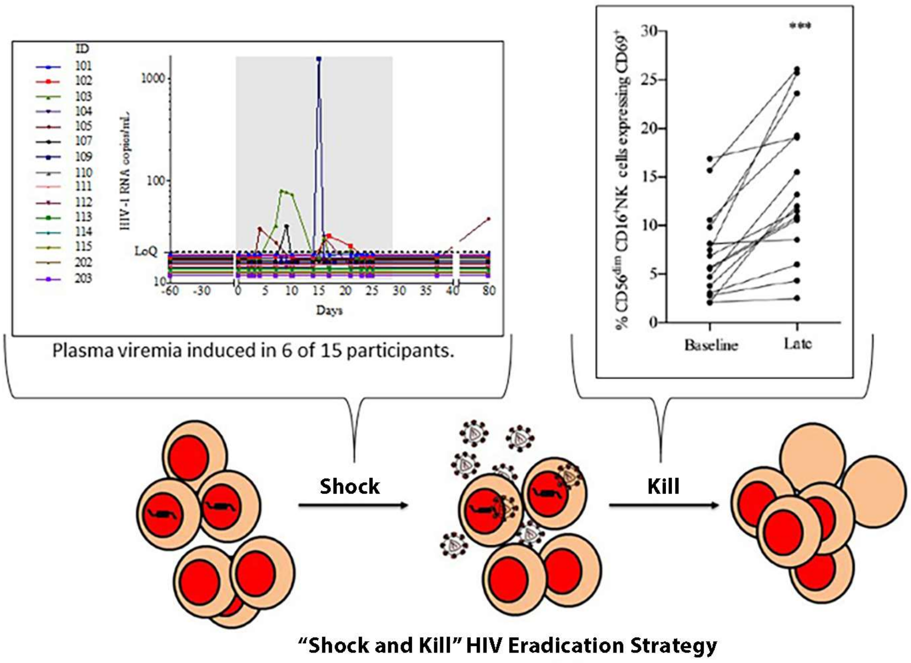

Figure 1. Potential dual role for lefitolimod in "shock and kill" HIV eradication strategies. On the left: HIV plasma viremia was observed in 6 of 15 participants. On the right: Significant increases in the activation of cytotoxic NK cells was observed during lefitolimod dosing. Significant activation of CD8+ T cells was also observed but is not shown here. (1)

To complement the general immunological effects of lefitolimod, we will utilize two highly potent bNAbs: 3BNC117 which targets the CD4-binding site on HIV envelope and 10-1074 which binds to the N332 glycan-site on the V3 loop of the virus envelope protein. A single 3BNC117 infusion lowers plasma HIV RNA by a mean of 1.48 log_{10} in viremic individuals (34) and multiple 3BNC117 infusions significantly prolonged time to viral rebound in ART-treated individuals undergoing analytical treatment interruption (35). A phase 1 trial with 10-1074 was recently completed and the drug was found to have similar antiviral potency and a longer half-life compared to 3BNC117 (Figure 2) (36). Specifically, a single 10-1074 infusion lowers plasma HIV RNA by a mean of 1.52 log_{10} in viremic individuals (36). The drop in viral load depended on the individual's starting viral load and also the sensitivity of their particular strains of HIV to the respective antibodies.

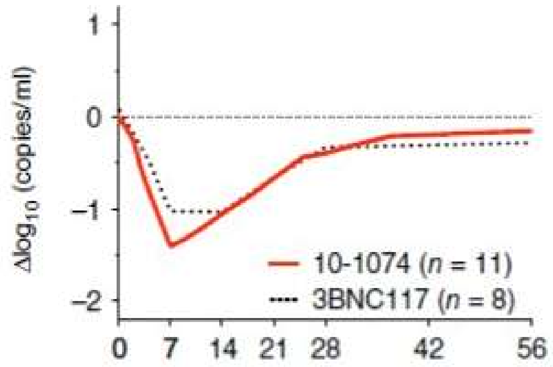

Figure 2. Monotherapy with 30 mg/kg of either 3BNC117 or 10-1074 causes a similarly potent reduction in HIV viremia. Graph shows average log10 change in viremia after 10-1074 (red line) or 3BNC117 infusion (dotted black).

Similar to monotherapy with antiretrovirals, resistance often develops during treatment with a single bNAb (34, 36). This emphasizes that combination bNAb treatment should be utilized to achieve broader coverage and to prevent or delay resistance development. *In vitro* TZM-bl neutralization assays as well as *in vivo* humanized mice and non-human primate studies have shown that treatment combining bNAbs with different binding-sites leads to more potent and durable viral suppression than any single bNAb alone (37-43). Intriguingly, in viremic individuals, bNAbs not only neutralize free virions and block viral replication, they also mediate killing of infected envelope positive cells via FcγR-dependent mechanisms (23) and significantly improved neutralizing responses to heterologous tier 2 viruses (44). These additional favorable mechanisms of action of 3BNC117 and 10-1074 also make them ideal components in approaches aiming to eradicate virus-producing cells in individuals on ART.

In summary, we provide a compelling rationale for combining lefitolimod with bNAbs in order to activate and kill virus-producing cells, but in addition to these immediate effects on persistently infected cells it is also conceivable that bNAbs in combination with lefitolimod will boost pre-existing and/or prime *de novo* HIV specific T and B cell responses. Thus, we hypothesize that co-administration of lefitolimod and bNAbs will (1) induce expression of HIV envelope in latently infected CD4+ T cells leading to their elimination through bNAb-binding and potentiated antibody-dependent effector mechanisms such as ADCC; and (2) elicit *de novo* humoral and cellular immune responses against HIV that will lead to enhanced immunological control in the absence of ART.

2.1.1 Overview of the Investigational Medicinal Products (IMPs)

2.1.1.1 Lefitolimod (MGN1703)

Lefitolimod (synonym dSLIM-30L1; dSLIM = **d**ouble **S**tem **L**oop **I**mmuno**m**odulator) is a novel synthetic DNA-based immunomodulatory molecule with two single-stranded loops separated by a double-stranded stem. Lefitolimod ~~is being~~ has been developed by MOLOGEN AG for the immunological treatment of persons with solid tumors. Current IMP ownership belongs to Gilead Sciences Inc (effective from June 20th, 2020) ~~and is currently in phase 3 testing for treating advanced stage colorectal cancer.~~

Lefitolimod consists of 116 nucleotides linked by phosphorodiester bonds and has a unique covalently-closed dumbbell-shaped structure without phosphorothioate modification (Figure 3). Each loop contains three non-methylated deoxycytidyl-deoxyguanosine motifs (CG-motifs) in a sequence environment known for its immunomodulatory potential.

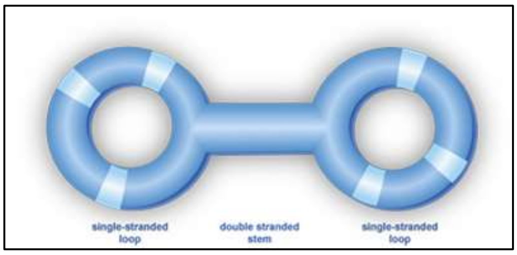

Figure 3. Structure of lefitolimod.

Lefitolimod can be compared with immunomodulatory oligonucleotides also containing CG-motifs (CpG-ODN), which usually are chemically modified (i.e. phosphorothioate backbone) for metabolic stabilization. In contrast to CpG-ODN, lefitolimod lacks these chemical phosphorothioate modifications and therefore, the toxicity and backbone-dependent off-target activity induced by these modifications, was expected to be absent. The absence of such toxicity has been confirmed by the animal toxicology studies and safety observations in human oncological trials reported so far (30, 32, 45). From the clinical trials with lefitolimod

it can be concluded that multiple subcutaneous administrations of 60 mg of lefitolimod are safe and well tolerated. Adverse events reported so far include mild injection-site reactions, nausea, transient neutropenia, fatigue, and mild to moderate flu-like symptoms lasting less than 48 hrs (such as pyrexia, increased body temperature, chills, headache, and myalgia). Such adverse events have to be treated in accordance with the current treatment guidelines (see section 2.1.5.1).

In concordance with the known actions of CpG-ODN, lefitolimod administration induces human peripheral blood mononuclear cells (PBMC) to generate a broad range of cytokines with strong anti-viral activity such as interferon (IFN)-α, IFN-γ, and interferon gamma-induced protein (IP)-10 (28). Importantly, IFN-α is secreted by the TLR9-positive plasmacytoid dendritic cells (pDC). In addition to cytokine/chemokine production, lefitolimod activates the TLR9 receptor expressing cells, pDC and B cells, in humans. Especially with pDC, immunologically relevant surface markers like CD80 and CD86 are up-regulated. Those co-stimulatory molecules are crucial for the stimulation of T cells and therefore for the induction of an adaptive immune response against HIV-1. Indirectly (via stimulation of TLR9-positive pDC and to a lesser extent B cells), lefitolimod administration leads to stimulation of TLR9-negative PBMC including myeloid DC, monocytes, natural killer cells, natural killer T cells and T cells (30). The broad activation of all those cell types indicates a strong induction of the innate and also the adaptive immune system necessary for an anti-viral immune response.

2.1.1.2 3BNC117 and 10-1074

Ten to 25% of HIV-1 infected persons develop sera that contain bNAbs including some bNAbs that neutralize the majority of viruses from diverse genetic subtypes (46-50). Early attempts to use passively transferred bNAbs to HIV-1 infected individuals undergoing treatment interruption showed limited effect (51, 52). These trials were performed before the advent of cloning and structure based design methods that uncovered anti-HIV-1 antibodies that have a broader spectrum of activity and are orders of magnitude more potent than neutralizing antibodies previously available (53-56).

3BNC117 is a monoclonal antibody (mAb) of the IgG1κ isotype that targets the CD4-binding site on the HIV envelope spike gp-120. 3BNC117 was isolated from an HIV-infected individual with high titers of bNAbs and who is an HIV-non-progressor (53). Cloning the heavy and light chain variable regions isolated from a single memory B cell generated the mAb. When compared with previously published VRC01 (a different bNAb) neutralization data, 3BNC117 is unusual in its potency and shows much greater inhibitory potential across a broad panel of HIV-1 (53). In preclinical studies carried out in humanized mice and non-human primates, 3BNC117 alone or in combination with other neutralizing antibodies led to protection from HIV-1 or SHIV infection and also to sustained suppression of (S)HIV-1 plasma viremia for prolonged periods of time (37-43).

10-1074 is a monoclonal antibody of the IgG1λ isotype that recognizes the base of the V3 loop and surrounding glycans on the HIV-1 envelope. 10-1074 was cloned from an African donor (ID 10) (46) infected with an HIV-1 clade A virus (57). In preclinical studies carried out in humanized mice and non-human primates, 10-1074 alone or in combination with other neutralizing antibodies led to protection from HIV-1 or SHIV infection and also to sustained suppression of (S)HIV-1 plasma viremia for prolonged periods of time (37, 43, 58, 59).

2.1.2 Clinical Experience

2.1.2.1 Lefitolimod (MGN1703)

Human experience with lefitolimod as monotherapeutic treatment for cancer includes a Phase 1 clinical study in patients with advanced solid tumors MGN1703-C01 (EudraCT: 2007-006291-10), and a Phase 2 clinical study in patients with advanced colorectal carcinoma MGN1703-C02 (IMPACT; EudraCT: 2009-017432-40). The formulation used since start of phase 2 has been evaluated in study MGN1703-C04 in 13 healthy volunteers for PK and cardiac safety.

Additionally, an exploratory randomized clinical phase 2 study (IMPULSE) investigated lefitolimod maintenance therapy in patients with extensive-disease small-cell lung cancer (ED-SCLC) after response to platinum-based first-line therapy MGN1703-03 (EudraCT: 2013-003503-19). The randomized study evaluated the efficacy and safety of lefitolimod and showed positive overall survival (OS) signals in two subgroups of patients in comparison to the control group (standard therapy). However, in this highly challenging indication

the primary endpoint OS was not met in the overall study population.

The pivotal phase 3 trial "IMPALA" MGN1703-06 (EudraCT: 2014-000834-50) is a randomized, parallel group, multinational trial in patients with unresectable metatstatic colorectal cancer and complete or partial response to induction treatment. This trial is currently ongoing and as of April 2017 nearly reached the targeted inclusion of 540 patients. Moreover, data were gained from five advanced cancer patients treated with lefitolimod in compassionate use programs and named patient use settings independently from clinical trials.

Furthermore, an IIT sponsored by the MD Anderson Cancer Center (USA) in patients with advanced solid malignancies started in June 2016, to explore safety, tolerability, and efficacy signals of lefitolimod administered s.c. or i.tu. in combination with ipilimumab.

Moreover, the Phase 1b/2a IIT "Toll-like receptor 9 enhancement of antiviral immunity in chronic HIV-1 infection: a phase 1b/2a trial (TEACH)" in patients with chronic HIV-1 infection was ongoing at the Aarhus University Hospital (Denmark). Part A of this trial was started on 30-Apr-2015 with 15 patients. Part B started on 25-Apr-2016 and the last patient was treated in May 2017. In total (part A and B) 20 patients have been exposed to lefitolimod.

By end of April 2017, 772 patients have been enrolled in the lefitolimod development program and thereof, 432 patients, and 13 healthy volunteers have been treated with lefitolimod (Berlin, Germany; MGN1703 Investigator's Brochure Version No.: 11; release date: December 13, 2017).

Data from HIV infected individuals

In our TEACH Part A study (1), 15 HIV+ individuals taking suppressive ART were dosed (60 mg subcutaneously) twice weekly for 4 weeks (1, 60). In accordance with the cell type–specific expression of TLR9, lefitolimod treatment led to pronounced activation of plasmacytoid dendritic cells and substantial increases in plasma interferon-α2 levels ($p<0.0001$). Consistently, transcription of interferon-stimulated genes (e.g. OAS1, ISG15, Mx1; each $p<0.0001$) were upregulated in CD4+ T cells as demonstrated by RNA sequencing. Further, proportions of activated cytotoxic NK cells and CD8+ T cells increased significantly during lefitolimod dosing, suggesting an enhancement of cellular immune responses. In 6 of 15 participants, plasma HIV-1 RNA increased from <20 copies/ mL up to >1500 copies/mL (range, 21–1571 copies/mL) during treatment (Figure 1). Thus, TLR9 agonist treatment in HIV infection has a dual potential by increasing HIV-1 transcription and enhancing cytotoxic NK cell activation, both of which are key outcomes in HIV-1 eradication therapy.

In our TEACH Part B follow-up to Part A (NCT02443935), we enrolled 14 HIV-1 infected individuals taking suppressive ART. Of the 14 enrolled participants, one participant withdrew informed consent after receiving study drug and one was lost to follow-up (after week 3). The remaining 12 (1 female and 11 male) participants completed treatment. Lefitolimod (60 mg subcutaneous) was administered twice weekly for 24 weeks while participants remained on ART. After the 24-week dosing period, 9 of 12 participants underwent an optional monitored analytical treatment interruption to assess time to viral rebound (i.e. HIV-RNA>5000copies/mL). Participants were randomized 1:1 to treatment interruption with either (a) 4 additional weeks of lefitolimod as monotherapy or (b) no additional study drug. Large blood volume (<250 mL) collections for multiple analyses were performed at baseline, during week 12, during week 24 and at time of viral rebound. (Unpublished data from this study are reported below)

Lefitolimod was safe and well tolerated. HIV specific immunity was assessed by CD8+ T cell intracellular cytokine stain for IFN-γ^+, TNF-α^+ and IL-2^+. This analysis revealed a significant ($p= 0.0068$) cohort-wide increase in IFN-γ response in HIV-specific CD8+ T effector memory cells and in terminally differentiated HIV-specific CD8+ T cells from baseline to after 24 weeks treatment. On a cohort level, HIV-1 DNA did not change significantly during the treatment period. However, upon interruption of ART, one individual (initiated ART during chronic infection, with pre-ART viral load of (log10) 4.03 copies/mL and nadir CD4 count of 29 cells/µL) demonstrated viral control to levels below limits of detection (20 copies/mL) for >21 weeks. Notably, this individual had a high percentage of polyfunctional HIV-specific CD8+ T effector memory cells (IFN-γ^+, TNF-α^+ and IL-2^+) which further increased 3-fold from baseline to end of treatment indicating that polyfunctional HIV-specific CD8+ T cells might have contributed to the observed virological control. Time to rebound for the remaining 8 individuals participating in the analytical treatment interruption was comparable to historical data on ART interruption.

B cell differentiation status in these individuals was measured on freshly isolated PBMCs (baseline, on week 12 and week 24) via flow cytometry. We found a substantial increase in the proportions of peripheral blood plasmablasts (week 12, p=0.033), which is the precursor of the antibody-secreting plasma cell. Consistent with these changes toward a more differentiated B cell phenotype we found increased levels of total IgG (p=0.019) as well as subclasses IgG1 (p=0.042), IgG2 (p=0.021), and IgG3 (p=0.002), after 12 weeks of treatment. Of note, the IgG3 subclass is superior in its binding affinity for Fc-receptors and is known to be particularly effective in the induction of effector functions (61).

Conclusion to date from the TEACH B study are as follows: Up to 24 weeks of lefitolimod treatment was safe and well tolerated in HIV patients on ART, corroborating the favourable safety profile already seen in cancer patients. This treatment enhanced HIV-specific T cell responses and might increase time to rebound in some individuals with strong polyfunctional HIV-specific CD8+ T effector memory cells responses. At the cohort level, lefitolimod markedly enhanced B cell differentiation in circulating B cells. The increase in plasmablasts is indicative of an enhanced differentiation of activated antibody-secreting plasma cells, which is supported by observed increase in IgG plasma levels in these individuals. Overall, these data suggest improved humoral immune response in HIV+ individuals on ART when treated with lefitolimod.

2.1.2.2 3BNC117

To date, 3BNC117 has been administered to 160 research participants (40 HIV-uninfected and 120 HIV-infected). It has been generally well tolerated at all dose levels tested (up to 30 mg/kg), with an estimated half-life of approximately 17.6 days in HIV-uninfected individuals and 9.6 days in viremic HIV-infected individuals (New York City, NY USA; 3BNC117 Investigator's Brochure Edition No.: 5; release date: May 15, 2018). 3BNC117 has shown *in vivo* antiretroviral activity in HIV-infected individuals when dosed at 10 or 30 mg/kg. 3BNC117 has also demonstrated exceptional breadth and potency against multiple HIV-1 clades when evaluated by an in vitro neutralization assay. It showed an average IC80 on a combined group of 95 tier 2 viruses of 1.4 µg/mL.

Phase 1 clinical trial (Protocol MCA-0835)

This protocol was an open label, dose-escalating, first-in-human phase 1 study to evaluate safety, pharmacokinetics and antiretroviral activity of 3BNC117 in HIV-uninfected and HIV-infected participants. The results are published (34). HIV-infected participants were either on or off ART at the time of enrollment, with HIV-1 viral loads < 100,000 copies/mL and absolute CD4+ T cell counts of > 300 cells/µl. Study participants were administered one or two intravenous infusions of 3BNC117 at one of four increasing dose levels (1 mg/kg, 3 mg/kg, 10 mg/kg and 30 mg/kg) and were followed for 24 weeks after last 3BNC117 administration. In total, 55 participants (22 HIV-uninfected, 17 viremic HIV-infected and 16 ART-treated HIV infected individuals) enrolled in the study. At the highest dosage level tested in the study, 30 mg/kg, all eight infected individuals treated showed rapid decrease in their amount of virus measured in their blood that varied between individuals from 0.8 to 2.5 on log10 scale (34). The median time to reach the lowest viral load was one week, but could be as long as three weeks. The drop in viral load depended on the individual's starting viral load and also the sensitivity of their particular strains of HIV to the antibody. In half of the individuals receiving the highest dose, viral loads remained below starting levels even at the end of the 8-week study period and resistance to 3BNC117 did not occur. It is likely that antibodies may be able to enhance the individual's immune responses against HIV, which can in turn lead to better control of the infection. In addition, antibodies like 3BNC117 may be able to kill viruses hidden in infected cells, which serve as viral reservoirs inaccessible to current ART.

Phase 2a study (Protocol MCA-0867)

This protocol was an open label, phase 2a study to evaluate the safety and antiretroviral activity of two or four 3BNC117 infusions in HIV-infected participants on combination ART during a brief analytical treatment interruption. Results from this study are published (35). Study participants were administered two 30 mg/kg intravenous infusions of 3BNC117 at weeks 0 and 3 (group A), or four infusions at weeks 0, 2, 4 and 6 (group B). ART was discontinued 2 days after the first 3BNC117 infusion. Participants were followed weekly and ART was resumed if viral rebound occurred (HIV-1 RNA > 200 copies/mL in 2 consecutive measurements) or CD4+ T cell counts declined to < 350 cells/mm3. Participants were followed for a total of 9 months after the first 3BNC117 infusion. The infusions were associated with a delay in viral rebound for 5-

9 weeks after 2 infusions, and up to 19 weeks after 4 infusions, or an average of 6.7 and 9.9 weeks respectively, compared with 2.6 weeks for historical controls (p=<1e-5) (35).

Sixteen participants enrolled and follow up was completed on January 25, 2017. All enrolled subjects reinitiated ART after viral rebound and achieved viral suppression. None of the participants experienced symptoms consistent with acute retroviral syndrome at the time of viral rebound.

Phase 2a Study (Protocol MCA-0866)
This study was a Phase 2a, open label study to evaluate the safety, antiretroviral activity and pharmacokinetics of four infusions of 3BNC117 in HIV-infected individuals on combination ART and during analytical treatment interruption. Seventeen study participants enrolled in the study and received up to four intravenous infusions of 3BNC117, administered at 30 mg/kg on day 0, week 12, week 24 and week 27. Antiretroviral therapy was discontinued 2 days after the third 3BNC117 infusion (week 24), until viral rebound (plasma HIV-1 RNA > 200 copies/ml in 2 consecutive measurements). Seventeen participants enrolled in protocol MCA-0866, fifteen participants completed the study, two dropped out prior to week 24. The fifteen participants who completed the study were followed for a total of 60 weeks from enrollment (day 0).

Additional Data
Nonclinical pharmacology studies in humanized mice and in non-human primates have shown that a single intravenous administration of 3BNC117 can protect animals from intravaginal or intra-rectal HIV-1 or SHIV challenge 24 hours later. In addition, 3BNC117 showed in vivo antiretroviral activity during chronic HIV-1 or SHIV infection. 3BNC117 effectively reduced HIV-1 or SHIV plasma viral levels alone or in combination with other anti-HIV-1 neutralizing antibodies. When administered alone, 3BNC117 transiently reduced HIV-1 plasma viremia in humanized mice, which was followed by virological rebound and selection of 3BNC117-escape mutants. In two non-human primates chronically infected with SHIV-AD8, both animals administered 3BNC117 alone experienced rapid declines of plasma viremia to background levels at day 10 following treatment. Single genome amplification did not identify 3BNC117-escape mutations following viral rebound. In both preclinical models, 3BNC117 led to prolonged suppression of plasma viremia when administered in combination with other broadly neutralizing antibodies. Suppression was maintained as long as mAb plasma levels were above a threshold of 1-5 µg/mL. (New York City, NY USA; 3BNC117 Investigator's Brochure Edition No.: 5; release date: May 15, 2018)

Summary
3BNC117 shows significant virologic activity when administered to viremic individuals at 30 mg/kg dose level. In addition, 3BNC117 can delay viral rebound when ART is discontinued in a subset of individuals harboring sensitive proviruses. Additional nonclinical and clinical data on 3BNC117 are collected in the Investigator Brochure (New York City, NY USA; 3BNC117 Investigator's Brochure Edition No.: 5; release date: May 15, 2018).

2.1.2.3 10-1074
To date, 10-1074 has been administered to 81 research participants at doses ranging from 3 to 30 mg/kg (32 HIV-uninfected and 49 HIV-1-infected), and there have been no significant adverse events related to 10-1074. To date, 59 individuals have been administered at least one dose of 30 mg/kg. Nonclinical and clinical data on 10-1074 are collected in the Investigator Brochure (36) (New York City, NY USA; 10-1074 Investigator's Brochure Edition No.: 3.0; release date: August 10, 2017) (New York City, NY USA; 3BNC117 Investigator's Brochure Edition No.: 5; release date: May 15, 2018).

Phase 1 Study (protocol MCA-0885)
In a recent proof-of-concept phase 1 human trial, 14 uninfected and 19 HIV-1-infected individuals were given a single dose of 10-1074 intravenously and monitored for 168 days (36). The preliminary PK data demonstrates that the half-life of 10-1074 in HIV-uninfected individuals is approximately 24.0 d, and 12.8 d in viremic HIV-1 infected individuals. At the highest dosage level tested in the study, 30 mg/kg, 11 of the 13 viremic individuals had 10-1074-sensitive virus. These 11 individuals showed a rapid decrease in viral load in their blood that varied between individuals from 0.9 to 2.06 on log10 scale. The nadir viral load was reached after an average of 10.3 d (range 7–25 d). The drop in viral load depended on the individual's starting viral load and also the sensitivity of their particular strains of HIV to the antibody.

In the half of the individuals receiving the highest 10-1074 dose, viral loads remained below starting levels even at the end of the 24-week study period and resistance to 10-1074 did not occur. It is likely that antibodies may be able to enhance the individual's immune responses against HIV, which can in turn lead to better control of the infection. In addition, antibodies like 10-1074 may be able to kill viruses hidden in infected cells, which serve as viral reservoirs inaccessible to current ART.

Phase 1b Study (Protocol MCA-0906) (combination treatment with 3BNC117 and 10-1074)
This is a phase 1b clinical trial to evaluate the safety, pharmacokinetics and the antiretroviral effects of 3BNC117 in combination with 10-1074, in HIV-infected individuals. Both antibodies are administered intravenously and in sequence. The study includes 5 study groups. Study participants are administered one or three intravenous infusions of 3BNC117 and 10-1074, each mAb dosed at 10 or 30 mg/kg:

- Single dose groups:

- Group 1A (n=6) - HIV-infected individuals, on ART with HIV-1 RNA < 20 copies/mL will be randomized in a 2:1 ratio to receive one intravenous infusion of 3BNC117 and 10-1074, each dosed at 10 mg/kg OR placebo (sterile saline), on day 0.

- Group 1B (n=6) - HIV-infected individuals, on ART with HIV-1 RNA < 20 copies/mL will be randomized in a 2:1 ratio to receive one intravenous infusion of 3BNC117 and 10-1074, each dosed at 30 mg/kg, OR placebo (sterile saline), on day 0.

- Group 1C (n=4) - HIV-infected individuals, off ART will be administered one infusion of 3BNC117 and 10-1074, each dosed at 30 mg/kg, on day 0.

- Three doses groups:

- Group 2 (n=15) - HIV-infected individuals, on ART with HIV-1 RNA < 20 copies/mL will be administered three infusions of 3BNC117 and 10-1074, each dosed at 30 mg/kg, on days 0, 21 and 42. Participants enrolled in Group 2 will discontinue their antiretroviral (ART) regimen on day 2.
- Group 3 (n=6) - HIV-infected individuals, off ART will be administered three infusions of 3BNC117 and 10-1074, each dosed at 30 mg/kg on days 0, 14 and 28.

Thirty-four participants have enrolled in protocol MCA-0906 and completed follow up. Two participants were lost to follow up after week 12 (group 1C) and week 27 (group 2). To date, only 3 participants enrolled in group 3 (New York City, NY USA; 3BNC117 Investigator's Brochure Edition No.: 5; release date: May 15, 2018).
Forty-five adverse events have been reported following 3BNC117 and 10-1074 infusions in protocol MCA-0906. Of these, 10 were of grade 2 severity and two were considered grade 3. Of the reported events, 5 were considered at least possibly related to the antibody infusions.

Phase 1 Study (Protocol YCO-899) (combination treatment with 3BNC117 and 10-1074)
Study YCO-0899 was a randomized, double blind, placebo-controlled phase 1 study to evaluate the safety and pharmacokinetics of the combination of 3BNC117 and 10-1074 in HIV-uninfected adults. The study consisted of three groups: Group 1, which was administered a single infusion of both antibodies at a dose of 10 mg/kg, Group 2, which was administered 3 infusions of both antibodies 8 weeks apart at a dose of 3 mg/kg, and Group 3, which was administered 3 infusions of both antibodies 8 weeks apart at a dose of 10 mg/kg. Each group consisted of 8 participants, of which 6 were randomized to receive active products and 2 were randomized to receive placebo. All participants were followed for 24 weeks after the last infusion. Twenty-four participants enrolled in protocol YCO-0899. All participants except one received all infusions as scheduled. One participant was lost to follow up, all other participants completed study follow up(New York City, NY USA; 3BNC117 Investigator's Brochure Edition No.: 5; release date: May 15, 2018).

Summary
10-1074 was safe and well tolerated at the doses up to 30 mg/kg in the first-in-human study testing its safety, pharmacokinetics and antiviral activity. In the 3BNC117 plus 10-1074 studies no seriuous adverse events have been reported by May 2018 and most reported AE's were graded as mild. Additional nonclinical and clinical data on 3BNC117 and 10-1074 are collected in the respective Investigator's Brochures (New York City, NY

USA; 3BNC117 Investigator's Brochure Edition No.: 5; release date: May 15, 2018) (New York City, NY USA; 10-1074 Investigator's Brochure Edition No.: 3.0; release date: August 10, 2017).

2.1.3 Pharmacokinetics (PK)

2.1.3.1 Lefitolimod (MGN1703)

The pharmacokinetics of lefitolimod were studied by Mologen AG (Berlin, Germany; MGN1703 Investigator's Brochure Version No.: 11; release date: December 13, 2017). Balb/c mice and cynomolgus monkeys were administered a single dose injection of lefitolimod and pharmacokinetic parameters were determined (Table 1).

Mice: Lefitolimod or PBS as control were administered subcutaneously to male and female Balb/c mice in order to investigate the pharmacokinetic profile (Cmax, Tmax and AUC) of lefitolimod. Lefitolimod doses administered were 7, 21 and 63 mg/kg body weight, respectively. Blood sample collections were set at pre-dose, 2, 4, 6, 8, 16, 24 and 96 h after subcutaneous injection.

Monkeys: Nine male and 9 female cynomolgus monkeys were selected for pharmacokinetic investigations. The animals were allocated to 3 test groups: a vehicle control, a low dose and a high dose group. Lefitolimod in doses of either 1.3 or 10.8 mg/kg body weight were administered by subcutaneous bolus injections into the dorsal region. PBS served as control. Animals were observed for body weight and clinical signs. Hematology parameters and differential blood count were determined 96 hr post administration. For serum and pharmacokinetic analysis blood samples were withdrawn from all animals pre-dose, 2, 4, 6, 16, 24 and 96 h after subcutaneous injection of lefitolimod. None of the animals died prematurely. There were no signs of local and systemic intolerance. The feces of all animals were normally formed throughout the study. No influence on body weight was seen. Lefitolimod had no effect on hematology parameters or differential blood count. Pharmacokinetic analysis revealed higher mean AUCs for females in comparison to male monkeys receiving the same dose. No clear conclusions were possible with respect to dose proportionality. When increasing the dose from 1.3 to 10.8 mg/kg body weight one would expect an increase in Cmax and AUC by a factor of 8.3 whereas the actual increase was 4.3-fold in female and 18-fold in male animals based on mean results for AUC_{inf}.

Table 1. Summary of animal pharmacokinetic data for Lefitolimod.

			Female					Male				
	Dose (mg/kg)		C_{max} (ng/ml)	T_{max} (h)	$AUC_{(0-24)}$ (h*ng/ml)	$AUC_{(0-inf)}$ (h*ng/ml)	$T_{1/2}$ (h)	C_{max} (ng/ml)	T_{max} (h)	$AUC_{(0-24)}$ (h*ng/ml)	$AUC_{(0-inf)}$ (h*ng/ml)	$T_{1/2}$ (h)
Mice	7	Mean (SE)	48.8 (18.3)	2	121.0 (36.9)	--	--	46.6 (1.89)	2	120.0 (6.45)	--	--
	12	Mean (SE)	172.0 (95.4)	2	477.0 (195.0)	--	--	178.0 (36.4)	2	489.0 (77.9)	--	--
	63	Mean (SE)	1251.0 (525.0)	2	4183.0 (1089.0)	--	--	785.0 (132.0)	2	3025.0 (290.0)	--	--
Monkeys	1.3	Mean (SD)	338 (276)	5.33 (2.31)	4327 (3015)	6460 (1851)	6.31 (0.01)	191 (96)	4.67 (3.06)	1935 (927)	2317 (1402)	7.50 (1.14)
	10.8	Mean (SD)	1088 (199)	2.00 (0.00)	11984 (2001)	28037 (10339)	6.05 (0.00)	1700 (593)	2.00 (0.00)	22498 (9561)	41905 (23151)	5.59 (0.38)

Note: Analysis included results lower than the lower limit of quantification of 1.0 * 10-11 g/µl (= 10 ng/ml) as quantitative values.

Humans: In addition to these animal studies, Mologen AG recently completed a cross-over PK clinical study: Protocol No. MGN1703-C04. The data are summarized here. Thirteen subjects who received both lefitolimod and placebo provided samples for PK analysis of serum concentrations of lefitolimod. All serum samples were collected as scheduled except for subject 1002, the samples scheduled at 24 and 72 hours after the administration of lefitolimod were collected at 24.1 and 72.1 hours, respectively. All values were used for the calculation of the pharmacokinetic parameters (**Figure 4**).

All 13 subjects received lefitolimod by a subcutaneous administration of 60 mg. The mean Cmax was 189 ± 101 ng/mL (range: 53.4 to 449 ng/mL) and the mean AUC0-t was 5001 ± 2518 ng•hr/mL (range: 1264 to

10984 ng•hr/mL). Although there was a moderately wide range in the Cmax and AUC0-t values, the Tmax and t1/2 were similar for all subjects. Tmax ranged from 8 to 16 hours, with a median of 14 hours. The values for t1/2 ranged from 9.36 to 17.9 hours, with a median of 12.7 hours.

Pharmacokinetic Parameters for MGN1703

Subject (n = 13)	Cmax (ng/ml)	T_{max} (hr)	AUC_{0-t} (ng•hr/mL)	$AUC_{0-\infty}$ (ng•hr/mL)	k_e (hr^{-1})	$t_{1/2}$ (hr)
Mean	188,7	12,9	5001	5012	0,0558	12,8
SD	101,5	3,1	2518	2519	0,0100	2,3
%CV for mean	53,8	24,2	50,3	50,3	17,9	17,9
Median	166	14	4340	4349	0,0544	12,7
Minimum	53,4	8	1264	1276	0,0388	9,36
Maximum	449	16	10984	10992	0,0741	17,9
Geometric Mean	166	na	4415	4428	0,0550	12,6
%CV for Geometric Mean	59,1	na	58,8	58,6	17,9	17,9

Mean Concentrations and Standard Deviations for 60mg MGN1703

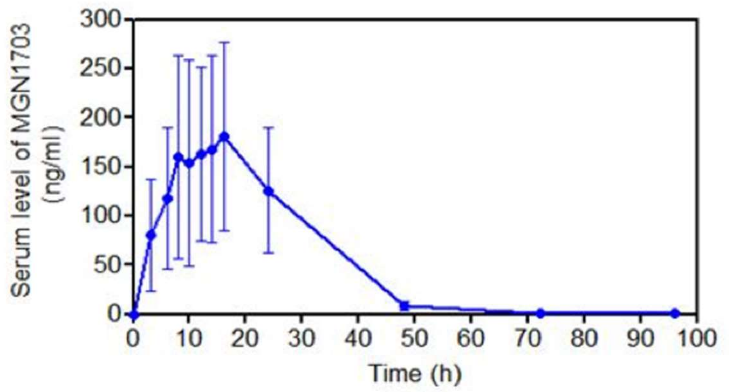

Figure 4. Table and figure summarizing the serum PK data from the 13 healthy participants in the Mologen cross-over study MGN1703-C04.

2.1.3.2 3BNC117

3BNC117 has favorable pharmacokinetic properties with a $T_{1/2}$ of 17.6 days in HIV-uninfected and 9.6 days in viremic HIV-1-infected individuals (**Figure 5**) (34). The elimination of 3BNC117 activity is thus more rapid in viremic HIV-1-infected individuals. 3BNC117 has pharmacokinetic properties consistent with a typical human IgG1 in uninfected individuals and a somewhat faster decay rate in HIV-1-viraemic individuals. Similar antigen-dependent enhanced clearance has been reported with anti-cancer antibodies. Although there may be other explanations, it is conceivable that the increased rate of antibody elimination in the presence of HIV-1 is due to accelerated clearance of antigen–antibody complexes.

2.1.3.3 10-1074

10-1074 has favorable pharmacokinetic properties with a $T_{1/2}$ of 24.0 days in HIV-uninfected and 12.8 days in viremic HIV-1-infected individuals (**Figure 6**) (36). The elimination of 10-1074 activity is thus more rapid in viremic HIV-1-infected individuals. 10-1074 has pharmacokinetic properties consistent with a typical human IgG1 in uninfected individuals and a somewhat faster decay rate in HIV-1-viraemic individuals. Similar antigen-dependent enhanced clearance has been reported with anti-cancer antibodies. Although there may be other explanations, it is conceivable that the increased rate of antibody elimination in the presence of HIV-1 is due to accelerated clearance of antigen–antibody complexes.

2.1.4 Interactions with other drugs:

2.1.4.1 Lefitolimod (MGN1703)

There are currently no known contra-indications for use of lefitolimod with concomitant medications.

2.1.4.2 3BNC117 and 10-1074

Small molecules are usually eliminated by non-catabolic pathways such as hepatic metabolism, renal excretion, and biliary excretion whereas mAbs are eliminated via catabolic processes. Since mAbs and small molecules do not share common or overlapping clearance mechanisms, mAbs are not predicted to affect directly the hepatic, renal, or biliary elimination of small molecules (62). Thus, it is highly unlikely that 3BNC117 or 10-1074 will lead to drug-drug interactions.

In addition, because the disposition of mAbs does not occur through non-catabolic pathways such as hepatic metabolism or transporters, they do not compete directly with chemically derived entities for these pathways. Thus, from a mechanistic perspective, the likelihood of direct drug-drug interaction (mAb as a victim) occurring during co-administration of other concomitant medications is unlikely to be high.

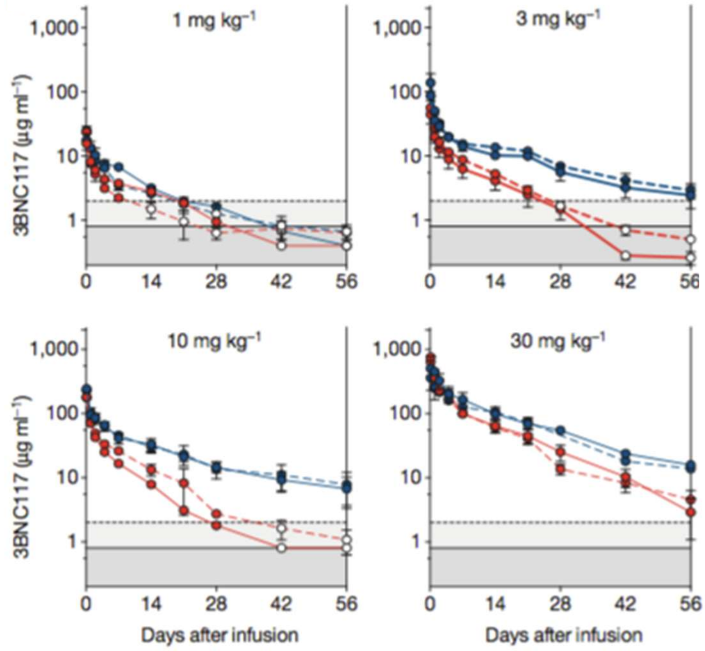

Figure 5. 3BNC117 decay measured in TZM-bl assays (solid lines) and ELISA (dotted lines). Mean values and s.e.m. for uninfected individuals (3 per group) are shown in blue and for HIV-1-infected individuals (2-5 per group) in red. Table summaries 3BNC117 PK based on a 56-day period post infusion.

Dose	HIV-1-status	Subjects	Method (subjects analyzed)	Cmax (μg/ml)			$t_{1/2\ (days)}$ (1)		
				Mean	SD	Range	Mean	SD	Range
30 mg	Neg.	3	ELISA (3)<br>TZM.bl (3)	**495.9**<br>**904.0**	233.1<br>281.7	360.8 - 765.0<br>1166.3 - 606.2	**15.0**<br>**16.4**	1.1<br>1.3	14.2 - 16.3<br>15.2 -17.9
30 mg	Pos.	6	ELISA (6)<br>TZM.bl (3)	**669.8**<br>**840.1**	199.7<br>118.4	410.2 - 976.4<br>717.4 - 953.7	**9.9**<br>**8.9**	3.3<br>2.3	5.8 - 13.7<br>6.7 - 11.2
All	Neg.	12	ELISA (9)<br>TZM.bl (9)	-<br>-	-<br>-	-<br>-	**17.2**<br>**17.6**	5.5<br>5.7	10.7 - 29.0<br>10.0 - 26.6
All	Pos.	15	ELISA (12)<br>TZM.bl (9)	-<br>-	-<br>-	-<br>-	**9.3**<br>**9.6**	2.6<br>2.9	5.7 - 13.7<br>6.1 - 13.7

(1) Estimation of half-lives; SD, standard deviation.

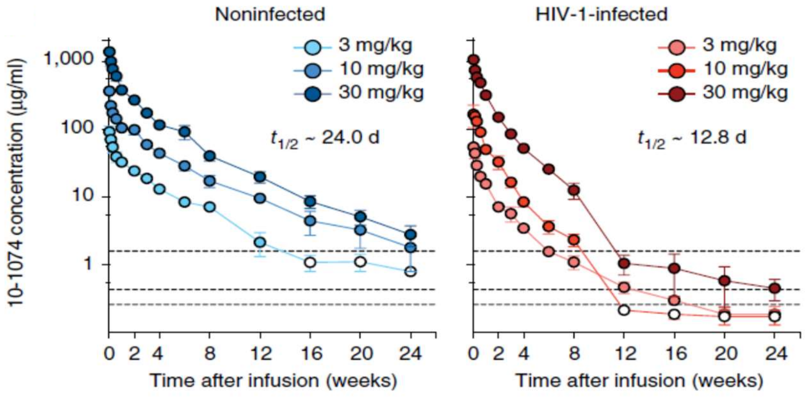

Figure 6. 10-1074 decay measured in TZM-bl assays (solid lines) and ELISA (dotted lines). Mean values and s.e.m. for uninfected individuals are shown in blue and for HIV-1-infected individuals in red. Table summaries 10-1074 PK based on a 168-day period post infusion.

10-1074 dose	HIV-1	# of subjects	C_{max} (μg/ml)			$t_{1/2}$ (days)		
			**Mean**	SD	Range	**Mean**	SD	Range
30 mg/kg	Negative	8	**1,417**	447.9	659.1 - 1,990	**23.0**	5.7	13.8 - 27.9
30 mg/kg	Positive	13	**1,076**	445.0	543.1 - 2,226	**11.9**	5.0	6.9 - 21.8
All	Negative	14				**24.0**	6.6	13.8 - 36.8
All	Positive	19				**12.8**	4.9	6.9 - 21.8

2.1.5 Safety Profile for each investigational medical product

2.1.5.1 Lefitolimod (MGN1703)

Single dose and multiple dose s.c. administrations of 0.25 mg to 60 mg were safe and well tolerated in all clinical studies conducted so far. (Berlin, Germany; Lefitolimod Investigator's Brochure Version No.: 11; release date: December 13, 2017). The most frequently reported drug-related AE in *Phase 1 clinical study MGN1703-C01* (patients with advanced malignant tumors) and the *Phase 2 clinical study MGN1703-C02, IMPACT* are mild to moderate fever, fatigue, chills, dizziness, headache, nausea, arthralgia, myalgia, hyperhydrosis, anorexia, malaise, weight increase, dyspepsia, night sweat, injection site itching, pruritus, rash, exanthema popular, paresthesia, and atypical pneumonia.

Drug-related laboratory abnormalities include only one instance each of increased aPTT and increased anti-nuclear antibody (ANA). Vital signs, ECG and physical examination have not revealed any dose- or time-related changes in course of treatment.

One patient discontinued the *Phase 2 clinical study MGN1703-C02* due to AE Grade 3 "sensory polyneuropathy" after treatment duration of 2.7 months. However, this patient received oxaliplatinum-based induction chemotherapy before inclusion into the study. Another patient developed mild to moderate "atypical pneumonia" for a total of 42 days leading to treatment stop, hospitalization and resulted in a SAE/SUSAR. The patient did not restart lefitolimod treatment, since the patient showed disease progression as well. Besides the above mentioned SAE "atypical pneumonia" no further lefitolimod-related SAE was reported in the described studies.

Furthermore, lefitolimod administrations were well tolerated. Local reactions comprised mainly mild redness and did not cause any physical or psycological discomfort in corresponding patients.
Lefitolimod-related AE include symptoms which are known for immune modulating drugs (i.e. general rash/itching, flu-like symptoms, injection site reactions, fatigue) and thus can be regarded as expected reactions.

One patient in the ongoing compassionate use program CU1 subsequent to the Phase 2 clinical study MGN1703-C02 developed a serious unexpected Grade 3 AE "membranous glomerulonephritis" leading to treatment stop after 57 months of treatment with lefitolimod. Biopsy revealed PLA2RAK and THSD7A positive membranous nephropathy. This supports the autoimmune nature of this case. However, the value of this finding is limited in distinguishing it from the background frequency of idiopathic membranous

nephropathy, which is linked to PLA2RAK in approximately 70% of cases or THSD7A in approximately 5% of cases. Primary membranous nephropathy has its peak incidence in the age group of the patient and accounts for approximately 75% of all membranous nephropathy cases. The patient was treated with six cyclophosphamide cycles at a dose of 1200 mg (absolute) as immunosuppressive treatment. Lefitolimod has been reported as a potential cause ("possibly related") for the event. Eight months after diagnosis of (03-May-2016) mGN the patient reported a good and stable general health status and a partial remission of mGN was documented by decreasing proteinuria (4,7g/day) from a maximum of 18g/day.

One patient of the *Phase 2 clinical study MGN1703-C03, IMPULSE* with ED-SCLC developed a Grade 2 leukoencephalopathy after platinum-based first-line therapy. Lefitolimod was assessed by the investigator as a potential cause ("possibly related") for the event based on the brain MRI pattern. In parallel, underlying ED-SCLC metastasizing in the brain (progressive disease) led to treatment stop; and one could argue that the reported event could also potentially be related to progression of disease under study. Moreover, previous chemotherapy administration of cisplatin/etoposide and the reversibility of the encephalopathy syndrome are possible hints that cisplatin/etoposide could also have been causal for this reversible encephalopathy syndrome. Within this trial, this was the only SAR reported.

The general safety results of the *Phase 1 study MGN1703-C04* (healthy volunteers) demonstrated that a single dose of 60 mg lefitolimod administered s.c. was safe and well tolerated. Seven of 13 subjects experienced 21 AE after receiving lefitolimod during the study, and 6 out of the 7 subjects experienced 16 AE that were considered possibly or probably treatment related. All AE were mild or moderate in severity. The most commonly experienced AE were ECG electrode application site irritation (lefitolimod arm, 23.1% vs. placebo arm, 21.4%) and headache (lefitolimod arm, 23.1% vs. placebo arm, 14.3%). Headache was the most commonly experienced AE related to treatment. There were no SAE reported during the study. No subjects discontinued from the study due to an AE. The ECG data indicate that a single dose of 60 mg lefitolimod did not have a negative effect on the cardiac safety within 5 days post dosing. Changes in ECG numerical data (HR, RR, PR, QRS, QT, QTc) were small and were similar in both lefitolimod and placebo treatment groups. The changes observed were consistent with expected spontaneous variability and circadian change, and therefore not clinically significant. Most ECGs were interpreted as normal, and the distribution of the few ECGs with abnormalities including ECG assessments which were interpreted as clinically significant in two patients were not consistent with a drug effect. At the end of study, both subjects had normal ECG assessments.

One patient with metastatic CRC from the phase III trial MGN1703-C06, IMPALA was hospitalized due to two subsequent episodes of fever (maximum of 39.5°C). The event was assessed as "possibly" related by the investigator leading to lefitolimod treatment stop. The patient received broad spectrum antibiotics (Piperacillin/Tazobactam) with good results – and was discharged fully recovered. Five days later fever reappeared without lefitolimod restart and the patient was hospitalized again with elevated leukocytes (17 x 10^9 /L) and elevated CRP (8.9 mg/dL). Intravenous broad spectrum therapy improved the clinical status and the patient was discharged as fully recovered. The sponsor's medical assessment of this event questions a potential causality of lefitolimod based on the facts that the event reoccurred again (second episode) without new exposure to lefitolimod. Moreover the patient showed positive response to antibiotic treatment and laboratory values suggested some inflammatory process which altogether makes the causality rather "unlikely".

Additionally, a patient from the IMPALA trial was hospitalized due to "nephrotic syndrome". The study medication was interrupted due to a chain of septic conditions. The intensity of the event was moderate and the outcome reported as "resolved/resolved with sequelae". The Investigator stated that while nephrotic syndrome may be secondary to colorectal carcinoma, both nephritis and nephrotic syndrome have been reported in patients treated with immunotherapy agents. The SAE "nephrotic syndrome" was assessed by the investigator as serious and possibly related to the study medication. The company agreed with the investigator assessment of seriousness but based on available safety data disagreed with the investigator assessment of causality. The underlying malignancy is a medically accepted alternative explanation and more likely the cause of nephrotic syndrome.

One further reported serious event was reported by a male patient of the phase III (IMPALA) trial randomized to lefitolimod coming to the emergency room because of hypotension (95/53). The patient received the study medication until two days prior to event onset "hypotension". The SAE "hypotension" was assessed by the study site investigator as possibly related to the study drug. The investigator stated that the event appeared 2 days after the study drug administration, so the event could be related with study drug.

The Medical Monitor disagreed with investigator causality assessment and stated the causality assessment as unlikely related based on the following: 1. In this patient two SAEs are reported as starting on the same date: SAE #119 "pulmonary toxicity/respiratory insufficiency to oxaliplatin" and SAE #118 "hypotension due to IMP". This provides a reasonable alternative causality, which has to be weighed against the temporal relationship. 2. Review including preliminary non-serious safety data from the IMPALA trial to date revealed in both arms a total of four non- serious cases of hypotension with equal distribution (2 cases in each arm) and not considered at least possibly related to the study drug. In conclusion hypotension is considered less likely related to the study drug than to pulmonary toxicity / respiratory insufficiency.

For the listed serious adverse reactions MedDRA based searches for potential similar (non-serious) events in an integrated lefitolimod safety database was not successful and no evaluated safety signal could be detected. The risk of local injection site reactions is confirmed by results from clinical trials with all such reactions being of mild to moderate severity and mainly of transient nature. The assessment of injection site related adverse reactions fails to raise concern about the local tolerability of the treatment with lefitolimod.
In pre-clinical settings administration of lefitolimod was associated with decreases in the absolute numbers of leucocytes, granulocytes and platelets and an increase in monocytes. In clinical trials neutropenia, anaemia, leukopenia, and thrombocytopenia as common reported adverse drug reactions of lefitolimod treatment have been observed. Except neutropenia all were of mild or moderate intensity. Neutropenia was reported with grade 4 intensity (CTCAE 4.03). However, no reported event was considered clinically immediately life-threatening. By assessing this risk the investigated patient population with advanced stage cancer diagnoses, the previous or accompanying chemotherapy treatment might also be taken into consideration as potential causal factor for these hematologic effects. During lefitolimod treatment the risk of hematologic adverse reactions has to be monitored through routine blood parameters testing. The risk-benefit profile of lefitolimod remains favorable and warrants its further clinical development.

2.1.5.2 Safety information related to antibody infusions

mAbs are a growing part of the therapeutic arsenal. While each mAb product has unique safety issues related to its mechanism of action, the major safety concern related to mAbs in general is an infusion/ hypersensitivity reaction. These types of reactions are more common for mAbs that contain murine elements, than humanized or fully human mAbs.

Passive administration of antibodies is successfully used to prevent or treat several viral diseases (63). For example, cytomegalovirus immunoglobulin is used for the prevention of transplant-associated infection. While human rabies immunoglobulin is used in conjunction with vaccine after suspected or proven exposure to rabies. Palivizumab, a humanized monoclonal antibody (IgG) directed against the fusion protein of respiratory syncytial virus, is the first monoclonal antibody approved for clinical use and it is indicated for the prevention of serious lower respiratory tract disease caused by respiratory syncytial virus in children at high risk of respiratory syncytial virus disease. Pavilizumab is generally safe and well-tolerated. Rare cases of severe hypersensitivity reactions (<1 per 100,000 recipients) have been described after an initial dose, as well as after re-exposure. Several other monoclonal antibodies are being developed for use in either prevention or treatment of other viral illnesses.

Passive administration of anti-HIV-1 antibodies has also been evaluated in humans. HIV Immune Globulin was in clinical use in the 1990s before the advent of highly effective ART. HIV Immune Globulin was also evaluated in HIV-infected pregnant females and their newborns in a phase III trial to assess whether HIV Immune Globulin plus single dose nevirapine given to mothers and infants would provide additional benefit over single dose nevirapine alone for prevention of peripartum HIV transmission. Women received a single intravenous infusion of HIV Immune Globulin 240 mL (approximately 200 mg/kg) at 36-38 weeks gestation. Infants born to these mothers received a single intravenous infusion of 24 mL (approximately 400 mg/kg) of HIV Immune Globulin, preferably within 18 hours of birth. Infusion-related events occurred in both mothers and infants but all infusion-related events resolved with no complications. While there was no demonstrable difference in treatment efficacy, the study showed that there were no significant differences in mortality or SAE between the two arms of the trial (64).

Several mAbs that target HIV-1 gp-120 have been evaluated in clinical studies. F105 is an IgG1κ human mAb that targets a discontinuous epitope that overlaps the CD4-binding site of gp-120. It was evaluated in phase I studies in HIV-1-infected individuals at 100 or 500 mg/m^2, single intravenous dose, and was found to

be safe and well-tolerated. Its $T_{1/2}$ was approximately 13 days (65). 2F5 and 4E10 are IgG1κ mAbs that target the membrane-proximal ectodomain of gp-41, while 2G12 binds to a carbohydrate moiety on the silent face of gp-41. These neutralizing antibodies were evaluated in combination in HIV-1-infected individuals. The first two studies included ART-naïve individuals with CD4+ cell counts >350 cells/mm^3, and plasma viral levels ≤10^4 in one study (n=7) or ≤10^5 in another study (n=8). The antibodies were administered intravenously at 0.5 to 1 g doses; 4 four infusions over a three week period and 8 infusions over a four week period. The antibodies were safe and well-tolerated and no clinical or laboratory abnormalities were observed throughout the studies. The median elimination $T_{1/2}$ of these antibodies were 6.6, 3.2 and 14.1 days for 4E10, 2F5 and 2G12, respectively. A low-level antibody response against 2G12 was found in two individuals. Anti-4E10 and anti-2F5 IgM and IgG immune responses were not detected, even after repeated infusions of high doses of the mAbs. Of note, the examination of plasma samples showed no detectable levels of immune complexes containing 2G12 and the evaluation of urine samples did not reveal elevated protein levels to suggest development of immune-complex induced renal disease (66, 67).

Two other studies included HIV-1-infected subjects on ART and plasma viral levels <50 copies/mL (n=14 and n=10). The antibodies were administered intravenously at doses ranging from 1 to 2 g for each antibody; 13 and 16 infusions were given over an 11 and 16 week period, respectively. ART was interrupted following 1 or 4 infusions. Antibody infusions were well-tolerated in most subjects; mild and transient side effects were reported only occasionally. No SAE were recorded. AE included body aches, fatigue, flushed sensation, joint soreness and redness at infusion site. Grade I post-partial thromboplastin time (PTT) prolongations were noted in one of the studies. Viral rebound was observed in eight of ten subjects (28 to 73 days post-ART interruption), while the two remaining subjects were aviremic over the course of the study (52). In seven of eight subjects with viral rebound, clear resistance to 2G12 emerged, whereas reductions in the susceptibilities of plasma-derived recombinant viruses to 2F5 and 4E10 were neither sustained nor consistently measured. Only two of eight individuals showed evidence of a delay in viral rebound during the passive immunization; whereas rebound upon cessation of ART was observed later in antibody-treated acutely infected individuals than in a control group of twelve individuals with acute infection (median of 8 weeks versus 3.75 weeks) (51). Though safe, the use of mAbs generally delayed, but did not prevent, viral rebound. The emergence of resistance to 2G12, however, demonstrated that the antibody exerted selective pressure on the circulating viral strains. It is important to note that the antibodies used in these studies have far lower potency and breadth than the more recently isolated neutralizing antibodies, such as 3BNC117 and 10-1074 (51, 52).

2.1.5.3 Safety data directly related to 3BNC117

To date, 3BNC117 has been administered to 160 research participants. It has been generally well tolerated at all dose levels tested (up to 30 mg/kg).

Phase 1 clinical trial (Protocol MCA-0835)(34)
3BNC117 was generally safe and well tolerated at the doses tested. No grade 3, 4 or serious adverse events, deemed at possibly related to 3BNC117 and no clinically significant treatment related changes in laboratory parameters occurred during study follow up. In total, 221 adverse events were reported during the study, and 70 were considered at least possibly related AEs. Of the related AEs, 59 (84%) were graded mild and 11 (15%) were graded moderate. The most commonly reported related AEs were headache and malaise or fatigue, which were transient and mild in severity in the majority of cases.

Phase 2a study (Protocol MCA-0867)(35)
Sixteen participants enrolled and follow up was completed on January 25, 2017. 3BNC117 showed similar safety profile as in protocol MCA-0835, and has generally been safe and well tolerated in both study group A and B. No grade 3, 4 or serious adverse events deemed at least possibly related to 3BNC117 and no clinically significant treatment-related changes in laboratory parameters have occurred during study follow up. In total, 56 adverse events were reported and 25 were considered at least possibly related to 3BNC117. Most reported AEs were grade 1 (n=44). Overall, the most commonly reported AEs were headache and upper respiratory tract infection. One participant with previous history of asthma was hospitalized with bacterial pneumonia 8 weeks after the second 3BNC117 infusion and while on ART. Another participant was hospitalized 30 weeks after the forth 3BNC117 infusion and while on ART with paresthesia of right upper and lower extremities and was diagnosed with a transient ischemic attack. Both serious AEs were considered not related to 3BNC117 or other study procedures, and both resolved without sequelae.

All enrolled subjects reinitiated ART after viral rebound and achieved viral suppression. None of the participants experienced symptoms consistent with acute retroviral syndrome at the time of viral rebound.

Phase 2a Study (Protocol MCA-0866)
This study was a Phase 2a, open label study to evaluate the safety, antiretroviral activity and pharmacokinetics of four infusions of 3BNC117 in HIV-infected individuals on combination ART and during analytical treatment interruption. Seventeen study participants enrolled in the study. Fifteen participants completed the study. In total 105 adverse events were reported during study follow up. Thirty-one were considered at least possibly related to 3BNC117 infusions, and 27 of those (87%) were grade 1. The most commonly reported AEs under protocol MCA-0866 were dizziness, nausea and malaise/fatigue.

Additional Safety and Efficacy Data
3BNC117 was well tolerated during an *in vivo* toxicology study in rats. There were no macroscopic or microscopic changes related to twice weekly intravenous administration of 3BNC117 over 25 days at doses of 4, 15, and 60 mg/kg. Chronic active inflammation with or without hemorrhage was noted at the subcutaneous injection site of rats following twice weekly subcutaneous administration of 3BNC117 over 25 days at a dose of 60 mg/kg. These findings correlated with macroscopic findings. Overall, due to the absence of 3BNC117-related changes, 60 mg/kg twice weekly for 4 weeks was considered the no observed effect level (NOEL).

In the human studies, the occurrence of ophthalmic complaints after 3BNC117 infusion was solicited at every follow up visit. Fourteen participants reported ophthalmic complaints. The complaints were transient mild eye twitching (2) 2 days and 4 weeks after infusion; periorbital edema/pain (1) 4 weeks after infusion; conjunctival erythema (2) 4 and 16 weeks after infusion; transient lacrimation (2) day of infusion; blurry vision (1) 7 weeks post infusion; pruritus (2) 1 week post infusion and (1) 4 months post infusion; dry eyes (1) 1 month post infusion; intermittent floaters in left eye (1) 20 weeks after infusion; diplopia (1) 5 days after infusion. A causal relationship with 3BNC117 has not been established. To investigate this, a tissue cross-reactivity study was performed on a full panel of tissues from humans and rats. This showed good concordance of binding between the two species. Membrane binding of 3BNC117 was restricted to two limited/rare cell types in conjunctival recesses and in the urinary bladder (neither of which correlated with findings in the repeat dose toxicology study).

3BNC117 has been evaluated in a first-in-human study in HIV-infected and HIV-uninfected subjects and in two other studies were the aim was to evaluate if 3BNC117 can maintain viral suppression during ATI in HIV-infected individuals and to evaluate the potential effects of 3BNC117 on the latent HIV-1 reservoir. In addition, 3BNC117 has been evaluated in combination with 10-1074 in YCO-899 and is currently being evaluated in MCA-0906 (for safety see section 2.1.5.4).
3BNC117 has been generally safe and well tolerated at doses up to 30 mg/kg. Mild transient fatigue and headaches have occurred. No grade 3, grade 4 or serious adverse events considered at least possibly related to 3BNC117 have been reported. Nonclinical and clinical studies performed to date support the expectation that repeated doses of 3BNC117 will be well tolerated and will demonstrate in vivo antiretroviral activity in humans.

Summary
In total, 382 adverse events were reported following 3BNC117 infusions under protocols MCA-0835, MCA-0866 and MCA-0867 (88 participants enrolled). Of these, 126 were considered at least possibly related to 3BNC117 infusion. Under YCO-899 and MCA-0906, 68 adverse have been reported and of these nine are considered at least possibly related to 3BNC117 and/or 10-1074.
Most reported related AE's under protocols with 3BNC117, were of graded 1 severity. The most common related AEs reported were headache, malaise/fatigue and upper respiratory infection, for all related adverse events, see table 2 (New York City, NY USA; 3BNC117 Investigator's Brochure Edition No.: 5; release date: May 15, 2018).

	No. AEs	No. Mild	No. moderate	No. Severe	No. Of participants	% of participants
Headache	20	16	4		16	11,8

Malaise / Fatigue	19	17	2		17	12,5
Upper respiratory tract infection	14	13	1		13	9,6
Nausea	11	11			11	8,1
Myalgia	8	6	2		5	3,7
Feverishness	7	4	2	1	5	3,7
Diarrhea	5	5			5	3,7
Infusion site pain or tenderness	5	5			4	2,9
Pruritus	5	4	1		4	2,9
Chills	4	2	2		2	1,5
Dizziness	4	4			3	2,2
Paresthesia	4	4			4	2,9
Infusion site induration, erythema or ecchymosis	3	3			3	2,2
Arthralgia	2	1	1		1	0,7
Blurry vision	2	2			2	1,5
Chest tightness	2	2			2	1,5
Elevated blood pressure	2	1	1		2	1,5
Eye twitching	2	2			2	1,5
Hyperhidrosis	2	2			1	0,7
Increased lacrimation	2	2			2	1,5
Bloating	1	1			1	0,7
Bradicardia	1		1		1	0,7
Conjunctival erythema	1	1			1	0,7
Diplopia	1	1			1	0,7
Dry mouth	1	1			1	0,7
Elevated bilirubin	2	1		1*	2	1,5
Flushing	1	1			1	0,7
Lightheadedness	1	1			1	0,7
Restlessness	1	1			1	0,7
Vomiting	1	1			1	0,7
Transaminitis	1	1			1	0,7
In total	135	116	17	2		

Table 2. Summary of all related AE's reported for 3BNC117. The table shows all related AE's reported under protocol MCA-835, MCA-866, MCA-867, YCO-899 and MCA-0906. A total of 136 participants have received doses of 3BNC117 under these protocols.
* A direct bilirubin of 0.7 mg/dL was recorded, which is considered a severe adverse event as per the DAIDS Toxicity Table Version 2.0

2.1.5.4 Safety data directly related to 10-1074

A total of 81 subjects have received 10-1074 at doses ranging from 3 to 30 mg/kg (32 HIV-uninfected and 49 HIV-1-infected). The first-in-human clinical trial (MCA-0885) included 33 subjects and is the only clinical trial where 10-1074 is given as monotherapy. 10-1074 was generally safe and well tolerated at the doses tested. No grade 3, 4 adverse events or serious adverse events were considered possibly related to 10-1074 and no clinically significant treatment-related changes in laboratory parameters occurred during study follow up. Nonclinical and clinical data on 10-1074 are collected in the Investigator Brochure (36).

Phase 1b Study (Protocol MCA-0906) (combination treatment with 3BNC117 and 10-1074)

This is a phase 1b clinical trial to evaluate the safety, pharmacokinetics and the antiretroviral effects of 3BNC117 in combination with 10-1074, in HIV-infected individuals. Both antibodies are administered intravenously and in sequence. By May 15, 2018, 45 adverse events have been reported in protocol MCA-0906. Ten of these were graded 2 of severity and two were considered grade 3. Of the reported events, 5

were considered at least possibly related to the antibody infusions. (New York City, NY USA; 3BNC117 Investigator's Brochure Edition No.: 5; release date: May 15, 2018).

Phase 1 Study (Protocol YCO-899) (combination treatment with 3BNC117 and 10-1074)
Study YCO-0899 was a randomized, double blind, placebo-controlled phase 1 study to evaluate the safety and pharmacokinetics of the combination of 3BNC117 and 10-1074 in HIV-uninfected adults. A total of 23 adverse events were reported during study follow up, most reported AEs were grade 1 (n=17, 74%). Sixteen were reported by 10 of the 24 participants administered 3BNC117 plus 10-1074, while the remaining 7 AEs were reported by 5 of the participants administered placebo. Four of the reported AEs by participants administered 3BNC117 plus 10-1074 were considered possibly related to 3BNC117/10-1074 infusions: fever/hyperthermia (n=1), malaise/fatigue (n=1), elevated total bilirubin of 1.3 mg/dL (n=1) and upper respiratory symptoms (n=1). The most common adverse event in both groups were upper respiratory tract infections (n=6) and headache (n=4).
The participant with fever/hyperthermia, had a temperature of 39.5 °C following 3BNC117 infusion. The elevated temperature of 39.5 °C (grade 3) occurred following the second 3BNC117 infusion. It was not associated with other symptoms or vital signs abnormalities and resolved with acetaminophen. Pre-infusion physical exam and laboratory tests were unremarkable. The participant remained asymptomatic 2 days after this episode during a follow up visit. Given the temporal relationship to the 3BNC117 administration this event was considered possibly related to 3BNC117. This participant was not administered the third 3BNC117/10-1074 infusions (New York City, NY USA; 3BNC117 Investigator's Brochure Edition No.: 5; release date: May 15, 2018).

Summary of Safety Data for 10-1074
In total, 68 adverse events have been reported following 3BNC117 and 10-1074 infusions under protocols MCA-0906 and YCO-0899. Nine were considered at least possibly related to 3BNC117 and/or 10-1074 infusions. Under protocol MCA-885 60 AE's were reported and of these 11 were considered related to 10-1074.
Most reported AE's under protocol MCA-885, YCO-899 and MCA-0906 were graded as mild, and the most commonly reported AEs were headache, malaise/fatigue, elevated bilirubin and upper respiratory infection (see table 3). No serious adverse events (SAEs) have been reported during study follow up (New York City, NY USA; 3BNC117 Investigator's Brochure Edition No.: 5; release date: May 15, 2018) (New York City, NY USA; 10-1074 Investigator's Brochure Edition No.: 3.0; release date: August 10, 2017).

	No. AEs	No. Mild	No. moderate	No. Severe	No. Of participants	% of participants
Headache	5	4	1		5	6.2
Dry eyes	1	1			1	1.2
Dizziness	1	1			1	1.2
Abdominal pain	1	1			1	1.2
Malaise / Fatigue	4	4			4	5
Pruritus	1	1			1	1.2
Elevated bilirubin	2	2			2	2.5
Upper respiratory infection	2	2			2	2.5
Fever / Hyperthermia	1			1	1	1.2
Transaminitis	1	1			1	1.2
Nausea	1	1			1	1.2
Total related AE's	20	18	1	1	20	

Table 3. Summary of all related AE's reported for 10-1074. The table shows all related AE's reported under protocol MCA-885, YCO-899 and MCA-0906. A total of 81 participants have received doses of 10-1074.

2.1.6 Rationale for dose selection

2.1.6.1 Lefitolimod (MGN1703)

Lefitolimod is formulated in phosphate buffered saline for subcutaneous (s.c.) administration. The standard dose of 60 mg lefitolimod is administered s.c. twice-weekly in single-agent oncology approaches. This schedule provides repetitive but transient immune surveillance reactivation according to lefitolimod's mode-

of-action. Due to the transient nature of the immune activation, downregulation of the TLR9 receptor and/or downstream signaling pathways which may occur during continuous stimulation is avoided.
Results from cancer patients include not only a favorable safety profile but clear pharmacodynamic effects like stimulation of cytokines/chemokines and immune cell activation. Notably, lefitolimod showed signals of efficacy in subgroups of cancer patients responding to first-line chemotherapy.
Using this dosing scheme, single-agent lefitolimod was evaluated in HIV-patients under ART within the TEACH trial, where systemic immune activation and even local immune responses (e.g. type-I-interferon in the gut) without inflammatory reaction were observed. However, the final goal to significantly reduce the HIV reservoir was not achieved except for one patient. Here, viral rebound was delayed for about 4.5 months. Thus, to further improve the immunological control over the virus, a modification of the dosing scheme is warranted (68).
In contrast to cancer patients who have responded to chemotherapy, HIV-patients treated with ART and exhibiting predominantly latently infected cells, harbor considerably less antigens for presentation and recognition by the immune system. Therefore, a stronger activation of the immune-system is needed, which may be achieved by an increased dose of lefitolimod, i.e 120 mg. However, in HIV patients under ART, especially in a placebo-controlled trial, a twice-weekly dosing is not feasible for compliance reasons. While, when using 120 mg once-weekly, the cumulative dose remains unchanged, the particular immune response to each administration should be increased. To achieve continued immunological control, lefitolimod is further combined with two broadly virus-neutralizing antibodies (bNAbs).
Notably, the use of 120 mg lefitolimod s.c. once-weekly is supported by a favorable safety profile observed in a combination study with the checkpoint inhibitor ipilimumab in vulnerable late-stage cancer patients in an ongoing trial (ClinicalTrials.gov Identifier: NCT02668770).

2.1.6.2 3BNC117

At the highest dosage level tested, 30 mg/kg, eight HIV-1-infected individuals treated showed rapid decrease in their amount of virus measured in their blood that varied between individuals from 0.8 to 2.5 on log10 scale, with most reaching their lowest viral load one week after treatment (34). The effect of 3BNC117 on viral loads was less pronounced in the lower dosing groups.

2.1.6.3 10-1074

At the highest dosage level tested, 30 mg/kg, eight HIV-1-infected individuals treated showed rapid decrease in their amount of virus measured in their blood that varied between individuals from 1.08–1.56 on log10 scale, with most reaching their lowest viral load one week after treatment (36). The half-life of 10-1074 is 12.8 days in HIV-infected individuals compared to 9.6 days for 3BNC117 and the dose is therefore lowered to 20 mg/kg for 10-1074, to obtain the same concentration of the two bNAbs (Figure 7).

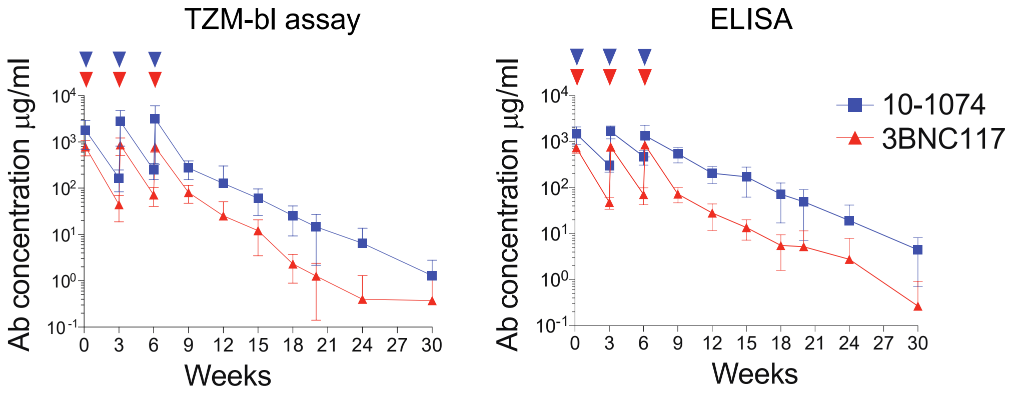

Figure 7. 3BNC117 and 10-1074 decay measured in TZM-bl assays and ELISA. Mean values and s.e.m. for HIV-infected individuals during ATI after 3 doses at 0-3-6 weeks.

3 Study Objectives and Endpoints

3.1 Objectives

3.1.1 Primary Objective

- To compare the effects of a TLR9 agonist (lefitolimod) and/or administration of potent bNAbs (3BNC117 and 10-1074) on time to viral rebound during analytical treatment interruption

3.1.2 Secondary Objective

- To evaluate the safety and tolerability of the Investigational Medicinal Products (IMP)s
- To compare viral load (plasma HIV-1 RNA) kinetics (e.g. doubling time) between study arms
- To compare time without ART between study arms

3.1.3 Exploratory Objective

- To evaluate the effect of the IMPs on the amount of HIV-1 DNA in CD4+ T cells
- To evaluate the effect of the IMPs on the functional HIV-1 reservoir in CD4+ T cells
- To compare HIV-specific immunity, T cell phenotype, immune activation, and cytokine production between study arms

3.2 Endpoints

3.2.1 Primary Endpoint

- Time from day of ART cessation until loss of virological control defined as the day on which plasma HIV-1 RNA levels have been sustained ≧1,000 copies/mL for ≧ 4 consecutive weeks or day of confirmed plasma HIV RNA >100,000 copies/mL.

3.2.2 Secondary Endpoints

- Safety evaluation, as measured by AEs, Adverse Reactions (ARs), SAEs, Serious ARs (SARs) and CD4 cell change from baseline to end of study.
- Rebound virus kinetics including time to >50 copies/mL and >1,000 copies/mL as well as doubling time during the analytical treatment interruption as measured by plasma HIV-1 RNA (Cobas TaqMan; Lower limit of quantitation 20 copies/mL)
- Time from day of cART cessation until day of cART re-iniation.

3.2.3 Potential Exploratory Analyses

- Quantitative HIV RNA/DNA measurements in CD4+ T cells by PCR, flow cytometry and/or imaging techniques (e.g. total and integrated HIV-1 DNA, RNA transcripts, and intact/defective virus RNA/DNA).
- HIV-specific T-cell immunity by intracellular cytokine staining (ICS).
- Plasma cytokine and immune activation biomarker levels
- Genetic, virological, and immunological predictors of treatment response
- Plasma 3BNC117 and 10-1074 concentrations
- Post-hoc sequence HIV-1 envelope and use machine learning technology to assess neutralizing sensitivity of bNAbs.
- Phylogenetic “foot-prints” of the investigational drugs as evidenced by the genetic make-up of viral reservoir, inducible virus and rebound virus during analytical treatment interruption.
- Size of the replication competent HIV-1 reservoir as determined by the number of infectious unit per million total CD4+ T cells using a viral outgrowth assay at baseline and prior to analytical treatment interruption.

4 STUDY DESIGN AND GENERAL PROCEDURES

4.1 Design

An investigator-initiated randomized, placebo-controlled, double-blinded international multicenter interventional phase IIa trial designed to evaluate the safety and efficacy of lefitolimod and 3BNC117/10-1074 in HIV-1-infected individuals on ART and during ATI as intervention to reduce the HIV-1 reservoir. Participants will be randomized 1:1:1:1 in a blinded fashion to receive (**Figure 8**):

Arm A: Placebo – Placebo
Arm B: Lefitolimod – Placebo
Arm C: Placebo – 3BNC117+10-1074
Arm D: Lefitolimod – 3BNC117+10-1074

Targeted enrollment is 48 study subjects – 12 in each arm.

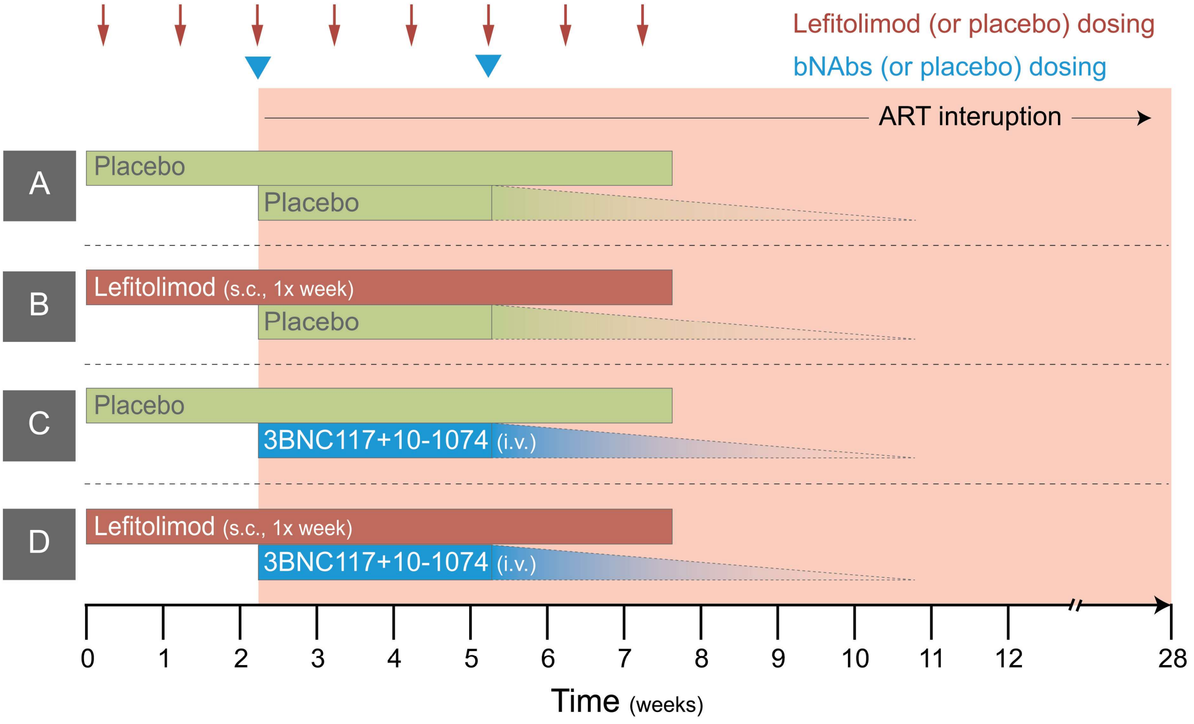

Figure 8. The four TITAN study arms. The red arrows indicate lefitolimod dosing (1x weekly). The blue arrowheads indicate administration of the bNAbs. The fading triangles represent the bNAb concentration decline following the second infusions.

4.2 Health Facilities

The trial will be conducted as an international multicenter study involving institutions in Denmark (including but not limited to the Department of Infectious Diseases at Aarhus University Hospital (AUH); The Department of Infectious Diseases at Aalborg University Hospital; The Department of Infectious Diseases at Odense University Hospital; The Department of Infectious Diseases at Hvidovre University Hospital; The Department of Infectious Diseases at Rigshospitalet), Australia (including but not limited to the The Peter Doherty Institute for Infection and Immunity, Melbourne), United States (including but not limited to the Department of Internal Medicine, University of Utah) and Norway (including but not limited to Department of Infectious Diseases, Oslo University Hospital).

The involved institutions in Denmark besides AUH may choose to screen and enroll individuals on-site, refer participants to AUH for investigational treatment, and continue follow-up visits at their site.

Study site	Site #

The Department of Infectious Diseases at AUH	101
The Department of Infectious Diseases at Aalborg University Hospital	201
The Department of Infectious Diseases at Odense University Hospital	301
The Department of Infectious Diseases at Hvidovre University Hospital	401
The Department of Infectious Diseases at Rigshospitalet	501
Infectious Diseases Clinical Research Unit at The Alfred Hospital, Melbourne	601
Department of Internal Medicine, University of Utah	701
Department of Infectious Diseases at Oslo University Hospital	801

4.3 Randomization

Randomization of subjects to one of the 4 arms will proceed through the use of a central computerized system at Aarhus University Hospital that is accessible to study sites 24 hours a day, 365 days a year. The aim is to include equal number of subjects to each of the 4 arms. To ensure correct randomization, the process will occur in randomly permuted blocks of 4 and 8. No stratification of participants will occur.
An unblinded person at the study site will be provided with a subject identification number and randomize to a study arm through the central computerized system. The subject identification number and the date of randomization will be provided to the study personnel and recorded in the CRF. Once subject identification numbers and randomization to study arm have been assigned, they cannot be reassigned. The computerized system will provide confirmation of the randomized study arm designation to the unblinded study site personnel who will retain the confirmation reports. On the site subject identification list, the site personnel will register subject identification number, randomization date, gender, last and first name, medical records identification (if applicable), and date of birth. All study personnel who directly interact with study participants are blinded to study arm designation. Pharmacy personnel and the personnel who prepare the IMPs for administration at the point-of care (as described in Section 6.6) are not blinded.

4.4 Discontinuation of Study

Study subjects can withdraw from the study or the IMP(s) in accordance with the conditions and procedures described in Sections 5.3 and 5.4 (Subject Withdrawal from Study and Subject Withdrawal from IMPs, respectively).

Premature discontinuation of the study may occur because of a regulatory decision, change in opinion of The Ethics Committee, drug safety problems or at the recommendation of the Safety Monitoring Committee (described in Section 9.2). The sponsor also has the right to temporarily suspend and/or discontinue the study due to, but not limited to, the safety of study subjects, ethical reasons, or serious problems of recruitment.

4.5 Source Data

The following documents are defined as source data:

- Informed consent and power of attorney
- Prints of study visit notes/information from electronic patient records
    - o Note of each visit in the patient's file
    - o Biochemical, immunological and virological measurements
- SAE reports

For the following information, the CRF will be the source data

- <u>Demographics:</u> Name, date of birth age, sex (male, female), and race/ethnicity, study identification number
- <u>HIV status:</u> Presumed date of infection, mode of acquisition of infection
- <u>ART regimen:</u> Start date, type of current regimen
- <u>Medical history:</u> Brief medical history, including list of medical conditions with year, site and sequelae
- <u>Concomitant medicine:</u> List of current medical treatment
- <u>Visit date</u>
- <u>Anthropometric data:</u> Weight, height, blood pressure, pulse and temperature
- <u>Physical exam:</u> Signs and symptoms noted by medical examination
- <u>Pregnancy status</u>
- <u>Contraception/pregnancy counselling:</u> type of contraception and start date

- <u>ECG</u>
- <u>Info on infusion:</u> start and stop time
- <u>AE, AR, SAE, and SAR reported</u>
- <u>Phone contact</u>
- <u>Extra visit</u>

Information on demographics, HIV status, ART regime, medical history and concomitant medicine will be obtained from patient journals.

5 SELECTION OF STUDY SUBJECTS

5.1 Inclusion Criteria

- Documented HIV-1 infection
- Adults age 18-65 years
- On ART for a minimum of 18 months.
- CD4+ T cell count >500 at screening
- HIV-1 RNA plasma level of < 50 copies/mL by standard assays for at least 15 months (a single viral load measurement > 50 but < 500 copies/mL during this time period is allowable).
- Able to give informed consent
- Viral reservoir sensitivity to 3BNC117 and 10-1074.
    - Sensitivity of the viral reservoir to neutralization by 3BNC117 and 10-1074 will be tested following the screening visit (i.e. prior to enrollment and randomization). Isolated PBMCs will be analyzed using the PhenoSense HIV mAb Assay, Monogram Biosciences. The sensitivity of an individuals archieved proviruses to bNAb neutralization will be determined by the IC50 value of PBMC derived pseudovirus inhibition. Subjects that are considered sensitive to both 3BNC117 and 10-1074 AND fulfill the other inclusion/exclusion criteria will proceed to study enrolment and randomization.
        - If sensitivity cannot be determined by the PhenoSense HIVmAb Assay, participants will be screened for 3BNC117 and 10-1074 sensitivity using HIV env sequencing carried out in-house (Aarhus, Denmark). The method was originally established and validated by Rockefeller University that already has this method implemented. The method utilizes HIV-1 DNA envelope sequencing and a mathematical prediction binding algoritm of known binding sites of the antibodies (69). Based on the individual HIV env sequence, proviruses are categorized as "sensitive" or "resistant". Subjects that are determined to be sensitive to both 3BNC117 and 10-1074 (defined as at least 90% of known sequences sensitive to either bNAb) AND fulfill the other inclusion/exclusion criteria will proceed to study enrolment and randomization.
        - In case, sensitivity cannot neither be determined by HIV env sequencing, so that both assays are inconclusive on 3BNC117 and 10-1074 sensitivity, the participants can still be enrolled at the discretion of the investigator. Participants with inconclusive results on both assay have a lower size of the HIV-1 reservoir as measured by total HIV-1 DNA (indicated with -PhenoSense-Env in Figure 9). Excluding these partipants would introduce selection bias.

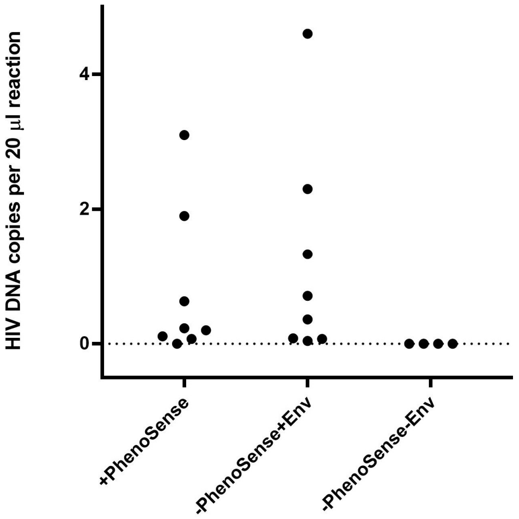

Figure 9. Size of the HIV-1 reservoir according to results of 3BNC117 and 10-1074 sensitivity on the PhenoSense HIVmAb Assay and/or HIV env sequencing.

5.2 Exclusion Criteria

- Any significant acute medical illness requiring hospitalization in the past 4 weeks
- Any evidence of an active AIDS-defining opportunistic infection
- Any condition that, in the Investigator's opinion, will prevent adequate compliance with study therapy
- The following laboratory values at screening, the values can be repeated within the screening period, but test results must be available before baseline (Day 0) and checked for eligibility:
    - Hepatic transaminases (AST or ALT) ≥3 x upper limit of normal (ULN)
    - Serum total bilirubin ≥3 ULN
    - Estimated glomerular filtration rate (eGFR) ≤50 mL/min (based on serum creatinine)
    - Platelet count ≤100 $x10^9$/L
    - Absolute neutrophil count ≤$1x10^9$/L
- Hepatitis B or C infection as indicated by the presence of hepatitis B surface antigen or hepatitis C virus RNA in blood
- History of:
    - Malignancy, excluding non-melanoma skin cancers, or organ transplantation
- Receipt of strong immunosuppressive or systemic chemotherapeutic agents within 28 days prior to study entry
- Known resistance to >2 classes of ART
- Known hypersensitivity to the components of lefitolimod, 3BNC117, 10-1074 or their analogues
- Pre-existing autoimmune or antibody-mediated diseases
- Women who are pregnant or breastfeeding, or with a positive pregnancy test as determined by a positive urine beta- human chorionic gonadotropin test during screening or women of child bearing potential* who are unwilling or unable to use an acceptable method of contraception (combined estrogen and progestogen hormonal contraception (oral, intravaginal or transdermal), progesteron-only hormonal contraception (oral, injectable or implantable), intrauterine device or intrauterine hormone-releasing system) to avoid pregnancy during the first 16 weeks of the study and while the HIV-1 RNA is detectable. Sexual abstinence will only be accepted in cases where this reflect the usual lifestyle.
- Males or females who are unwilling or unable to use barrier contraception during sexual intercourse until plasma HIV-1 RNA is undetectable using standard assays

* Women are considered of childbearing potential following menarche and until becoming post-menopausal unless permanently sterile. Permanent sterilization methods include hysterectomy, bilateral salpingectomy or bilateral oophorectomy (According to the Clinical Trial Facilitation Group, 2014-09-15).

5.3 Subject Withdrawal from Study

Subject withdrawal from study is defined as any subject who does not complete the final follow-up visit (visit 11) as defined in this protocol.
Reasons why a study subjects may be withdrawn from the study include, but are not limited to:

- Subject request (withdrawal of consent)
- Protocol violation
- AE or reactions
- Any condition, interaction, or contraindication where continued participation in the study will result in an unacceptable risk for the subject, as assessed by the Investigators or advisers
- Discontinuation of the study by the Sponsor
- Lost to follow-up

Investigator will contact subjects who fail to return for planned visits and if possible schedule a new visit. Information related to study withdrawal is documented in the CRF including the reason for withdrawal, date of withdrawal, and whether the subject or Investigator made this decision. Subjects withdrawn from the study will contribute with data for the statistical analyses until the date of withdrawal.
Subjects withdrawn from the study will resume routine treatment and control according to standard treatment guidelines.

Subjects withdrawn from the study after the point of randomization will not be replaced.

5.4 Subject Withdrawal from the Investigational Medicinal Product(s) (IMPs)

Withdrawal from the IMP(s) is defined as a subject who discontinues study treatment but agrees to continue follow-up until completion of the study at visit 11. Any subject who withdraws from the IMP(s) after having received at least one dose will be asked to return for follow-up visits 1 week after withdrawal from the IMP(s) (safety monitoring) and final follow-up visits at week 4 after withdrawal from the IMP(s). If withdrawal from the IMP(s) is due to AE, this will be followed up as detailed in Section 7.6 (Safety Monitoring).

Reasons why a study subjects may be withdrawn from the IMP(s) include, but are not limited to:

- Subject request (withdrawal of consent)
- AE or reactions
- Any condition, interaction, or contraindication where continued participation in the study will result in an unacceptable risk for the subject, as assessed by the Investigators

Information related to withdrawal from the IMP(s) is documented in the CRF including the reason for withdrawal, date of withdrawal, and whether the subject or Investigator made this decision. Subjects withdrawn from the IMP(s) will contribute with data for the statistical analyses until the date of withdrawal. Subjects withdrawn from the IMP(s) will resume routine treatment and control according to standard treatment guidelines.

6 STUDY TREATMENT

6.1 The Investigational Medicinal Products (IMPs)

The first IMP is defined as lefitolimod, a TLR9 agonist (120 mg subcutaneously once weekly for 8 weeks). The second and third IMPs are defined as 3BNC117 and 10-1074, respectively. 30 mg/kg of 3BNC117 and 20 mg/kg of 10-1074 will be sequentially administered intravenously over a 1-hour period each on visit 4 and visit 7. Physiological saline will be placebo for both lefitolimod (arm A and C) and for 3BNC117 and 10-1074 (arm A and B).
The study will be conducted among HIV-1-infected individuals undergoing suppressive ART. Subjects will continue background ART while receiving lefitolimod, 3BNC117 and 10-1074 until the analytical treatment interruption is initiated after visit 4.

6.2 Dosing Modifications

6.2.1 Lefitolimod (MGN1703)

If neutropenia (Grade 3 or higher) is observed, doses will be skipped until neutrophil levels are within the normal range.

6.2.2 3BNC117

3BNC117 dose modification is not allowed.

6.2.3 10-1074

10-1074 dose modification is not allowed.

6.2.4 Placebo - sterile physiological saline

Placebo dose modification is only allowed if neutropenia (Grade 3 or higher) is observed and the investigator suspects lefitolimod/placebo to be the cause, doses will be skipped until neutrophil levels are within the normal range.

6.3 Consent Procedure

Prior to the initiation of any study related procedures, the potential subjects will be given a copy of the most recent subject information sheet and informed consent to read. Additionally, the principal investigator or a study physician who has been designated to consent will discuss the specifics of the study including but not limited to the purpose of the research, procedures, time commitment, required tasks, IMP(s), alternative treatments, benefits, risks, confidentiality etc. in a comprehensible (non-scientific) manner, using language readily understandable by the subject. Subjects will be told that participation is voluntary and that, if they do

not consent, they will not be penalized. The person consenting will assure the voluntariness of the subject. Deliberation time is minimum 24 hours.

A private, confidential setting will be provided for the potential subject to read and discuss the informed consent free from coercion, undue influence or constraints of time. All subjects will be given a chance to ask questions and express concerns. They will be given the option to take the consent home and discuss it with family, friends, and/or health care providers. After a subject and the person conducting the consenting signs and dates the consent, the subject will be given a copy of the signed informed consent form.

By given informed consent the subject is informed, that information from the source data (Section 4.5; Source Data) is available to the Sponsor, the regulatory authorities and Lead Principal Investigator.

6.4 Drug Supplies, Packaging and Labelling

6.4.1 Lefitolimod (MGN1703, Mologen AG for Gilead Sciences Inc.)

Lefitolimod is manufactured by Mologen AG, Berlin, Germany for Gilead Sciences Inc. This TLR9 agonist consists of 116 nucleotides linked by natural phosphorodiester bonds and has a unique covalently-closed dumbbell-shaped structure, it avoids chemical phosphorothioate modifications. Each loop contains three non-methylated deoxycytidyl-deoxyguanosine motifs (CG-motifs) which, surrounded by a specific sequence are known for their immunomodulatory potential. The labeled study drug will be provided by Gilead Sciences Inc. with a concentration of 15 mg/mL in phosphate buffered saline. One vial contains 2.0 mL (with a 10% overfill) resulting in 30 mg lefitolimod. Currently two vials are packed into a box (1 kit) therefore two kits will be used for one administration of 120 mg.

Packaging and labelling of lefitolimod (MGN1703) will be performed by Mologen AG and IMP is released in accordance with national regulatory requirements. The Hospital Pharmacy at AUH, Denmark will re-label lefitolimod with the updated expiry date and release of the IMP will be certified by a Qualified Person in accordance with national regulatory requirements. For further details on physical, chemical and pharmaceutical properties we refer to the Investigator Brochure.

6.4.2 3BNC117

3BNC117 is manufactured by Celldex Therapeutics, Inc., New Jersey, US. 3BNC117 is manufactured in accordance with Good Manufacturing Practices. 3BNC117 is a recombinant fully human mAb of the IgG1κ isotype that specifically binds HIV envelope gp120. 3BNC117 is a clear liquid, provided in single-vials containing 10 mL of product at a 20 mg/mL concentration.

Packaging and labelling of 3BNC117 will be performed by Clinigen Clinical Supplies Management, Frankfurt, Germany and released in accordance with national regulatory requirements. If re-labelling of 3BNC117 due to extension of the expiry date is needed, this will be done at the Hospital Pharmacy at AUH, Denmark, and release of the IMP will be certified by a Qualified Person in accordance with national regulatory requirements. The 3BNC117 dosing packs will be prepared on study site (Section 6.6; Drug Preparation and Administration). Labelling will contain project name, and manufacturing date and time. For further details on physical, chemical and pharmaceutical properties we refer to the Investigator Brochure.

6.4.3 10-1074

10-1074 is manufactured by MassBio, Massachusetts Institute of Technology, Cambridge, US. 10-1074 is manufactured in accordance with Good Manufacturing Practices. 10-1074 is a recombinant fully human mAb of the IgG1λ isotype that binds to the N332 glycan-site on the V3 loop of the virus envelope protein. 10-1074 is a clear liquid, provided in single-vials containing 30 mL of product at a 20 mg/mL concentration.

Packaging and labelling of 10-1074 will be performed by Clinigen Clinical Supplies Management, Frankfurt, Germany and released in accordance with national regulatory requirements. If re-labelling of 3BNC117 due to extension of the expiry date is needed, this will be done at the Hospital Pharmacy at AUH, Denmark, and release of the IMP will be certified by a Qualified Person in accordance with national regulatory requirements. The 10-1074 dosing packs will be prepared on study site (Section 6.6; Drug Preparation and Administration). Labelling will contain project name, and manufacturing date and time. For further details on physical, chemical and pharmaceutical properties we refer to the Investigator Brochure.

6.4.4 Placebo - sterile physiological saline

The placebo for lefitolimod, 3BNC117 and 10-1074 is locally available sterile physiological saline (eg. Sodium choloride USP 0.9% for injection). Sterile physiological saline to be used as placebo for lefitolimod will be provided in small (e.g. 10 mL) single use vials. Placebo for 3BNC117 and 10-1074 will the be same volume of saline (250 mL) as used for regular bNAb infusions but without any study compound (Section 6.6; Drug Preparation and Administration). Labeling will contain an expiration date and time.

6.5 Drug Storage and Accountability

The Sponsor and/or Investigators are responsible at AUH, Melbourne, San Francisco and Oslo are responsible for secure and correct storage of lefitolimod after arrival at study site or pharmacy. The hospital pharmacies at AUH (and other Danish sites), Melbourne,San Francisco and Oslo are responsible for secure and correct storage of 3BNC117 and 10-1074 at the study sites. The IMPs will be stored in appropriate conditions in a secure location with controlled access. The storage compartment shall be monitored regularly and the temperature shall be documented. Lists of received, used and remaining quantities of the IMPs will be kept. Any discrepancies must be solved.

Until dispensed to subject the IMPs should be stored as follows:

- Lefitolimod: Store between 2-8°C
- 3BNC117: Store between 2-8°C
- 10-1074: Store between 2-8°C
- Placebo - sterile physiological saline: room temperature

6.6 Drug Preparation and Administration

Subcutaneous injections will be administered by trained study personnel with experience dosing medication in this manner. Infusions will be administered by trained study personnel with experience dosing intravenous medication and training to immediately respond to acute infusion-related reactions. (These persons are referred to here as: “administering personnel”).

At study entry, study personnel will calculate the dose (3BNC117 and 10-1074) for each participant regardless of study arm designation. For dosing appointments, the unblinded personnel at each site can access the centralized (at AUH) randomization portal and be informed of a study participants’s study arm designation in an unblinded fashion (as described in Section 4.3). This will permit the unblinded site personnel to order and/or prepare the proper IMP(s) for a given individual for each dosing visit.

Because the placebo is not specifically manufactured by the producers of the IMPs, it is not possible for the placebo packaging to mimic that of the IMPs. Therefore, in order to ensure the continued blinding of the administering personnel and the participant, a nurse or similarly qualified point-of-care designee (referred to here as: “preparation designee”) will prepare the IMP(s) in a manner so that the administering personnel remains blinded to the IMP packaging.

The preparation designee will strictly maintain confidentiality regarding the product(s) identification as they prepare the IMP(s) for injection (lefitolimod or placebo) or infusion (3BNC117_and_10-1074 or placebo_and_placebo). This preparation step and packaging waste disposal will occur in an area that cannot be observed by the administrating person or the participant (e.g. an adjacent room). All packaging materials will be disposed of in a non-transparent plastic bag and placed in the proper waste receptacle. Disposal of IMP packaging in this manner will prevent any incidental recognition of the contents by administering personnel or participants.

IMPs will be presented by the preparation designee to the administering personnel labeled only with the participant’s ID number to avoid indicating whether the contents are active drug or placebo. In the case of the IV bags for infusion dosing, the bags will be also labeled “first” or “second” to indicate the order of administration. For consistency, 3BNC117 or placebo will always be infused first and 10-1074 or placebo will be infused second.

For lefitolimod or placebo subcutaneous dosing: four syringes, each containing 2 mL of IMP, will be provided ready for injection to the administering personnel.

For 3BNC117_and_10-1074 or placebo_and_placebo infusions: two IV bags, each containing 250 mL of IMP, will be provided ready for infusion to the administering personnel.

6.6.1 Lefitolimod

The labeled study drug will be provided by Gilead Sciences Inc. ~~Mologen AG~~ with a concentration of 15 mg/mL in phosphate buffered saline. One vial contains 2.0 mL (with a 10% overfill) resulting in 30 mg lefitolimod. Currently two vials are packed into a box (1 kit), therefore two kits will be used for one administration of 120 mg. Injections are administered subcutaneously by the study investigator or study co-ordinator as four 2-mL bilateral injections. The bilateral subcutaneous dosings are to alternate between the upper arm + abdomen and thigh + abdomen. [e.g. The first bilateral doses (Day 0) will be administered in the upper arm adjacent to the bicep muscles and in the abdomen adjacent to the external oblique muscles. The second bilateral doses (Day 7) will be administered in the thigh adjacent to the rectus femoris muscles and in the abdomen adjacent to the external oblique muscles. The sequence begins again with the third bilateral doses (Day 14) being administered in the upper arm adjacent to the bicep muscles and in the abdomen adjacent to the external oblique muscles. The sequence continues until all dosing has been completed (Day 49)]

6.6.2 3BNC117

3BNC117 will be provided in single-use vials containing 10 mL of 3BNC117 at a 20 mg/mL concentration. The appropriate dose will be calculated (30 mg/kg) at the study site according to subject's weight. Before intravenous infusion, 3BNC117 is further diluted to a volume of 250 mL in sterile physiological saline for injection, USP. 3BNC117 will be good for infusion for 3 hours after it has been diluted to its final volume of 250 mL and will be administered over 1 hour. Study individuals will be monitored for 1-hour post infusion at the study site.

6.6.3 10-1074

10-1074 will be provided in single-use vials containing 30 mL of 10-1074 at a 20 mg/mL concentration. The appropriate dose will be calculated (20 mg/kg) at the study site according to subject's weight. Before intravenous infusion, 10-1074 is further diluted to a volume of 250 mL in sterile physiological saline for injection, USP. 10-1074 will be good for infusion for 3 hours after it has been diluted to its final volume of 250 mL and will be administered over 1 hour. Study individuals will be monitored for 1-hour after the last infusion at the study site.

6.6.4 Placebo - sterile physiological saline

Sterile physiological saline will be provided by a locally available distributor in single-use vials containing 10 mL or 250 mL infusion bags as placebo for lefitolimod and 3BNC117_10-1074, respectively. The placebo volume for lefitolimod (4 times 2 mL) will be utilized from a placebo vial, as appropriate to match the active product for which the placebo is being provided. The 250 mL infusion bags will be indentical to those used for dilution of 3BNC117 and 10-1074.

6.7 Concomitant Medication(s)

Subjects are required to continue ART regimen in the first two weeks of the study, after this point subjects are asked to discontinue ART. Any unplanned changes in ART during the study will be recorded in the CRF. Any other current medical therapy either recorded at study entry or initiated during the study will be recorded in the CRF. Initiation of medical therapy specified in the exclusion criteria will result in withdrawal from the IMP(s).

7 STUDY OVERVIEW

7.1 Study visits (Outlined in Table 4)

Prior to enrollment and initiation of study-related procedures, written informed consent must be obtained from the subject. In general, any new or previously identified signs or symptoms that the subject has experienced since the last visit will result in a targeted physical examination and if relevant appropriate diagnostic tests.

7.1.1 Visit 1/Day -140 to -15; screening

Potential subjects will undergo screening assessment to evaluate eligibility. The following evaluation will be performed:

- Review of inclusion/exclusion criteria
- If female; pregnancy test (serum/urine)
- Instruction about pregnancy precautions
- Instruction about barrier contraceptions
- Demographics
- Concurrent illness
- Medication history
- Medical and surgical history. Regarding HIV history, the following should be recorded, if possible:
    - HIV-1 diagnosis date
    - Presumed date of infection with HIV-1
    - The last CD4+ T cell measurements prior screening
    - The last viral load measurements prior screening
    - HIV-1 clade, if available
    - Mode of HIV acquisition
- Vital signs (blood pressure, heart rate, ECG, temperature)
- Physical examination (including height and weight)
- Blood samples (70 mL)
    - Routine biochemistry
    - Hepatitis status: HBsAg + anti-HCV (and if positive HCV RNA)
    - HIV viral load
    - T cell counts
    - Analysis of proviral susceptibility to 10-1074 and 3BNC117 neutralization

7.1.2 Visit 2/Day 0. Baseline. Initiation of interventions

- Medical History
- Directed physical exam (adverse events, local/systemic reaction)
- If female; pregnancy test (serum/urine)
- Blood collection (250 mL)
    - Routine biochemistry
    - HIV viral load
    - T cell counts
    - Exploratory analyses
- Stool sample (optional)
- Lefitolimod/placebo dosing (always dosed after blood collection)

7.1.3 Visit 3/Day 7 (+/- 3 day)

- Medical History
- Directed physical exam (adverse events, local/systemic reaction)
- Blood collection (4 mL)
    - HIV viral load
- Lefitolimod/placebo dosing (always dosed after blood collection)

7.1.4 Visit 4/Day 14 (+/- 3 day)

- Medical History
- Directed physical exam (adverse events, local/systemic reaction)
- Blood collection (120 mL)
    - Routine biochemistry
    - HIV viral load
    - T cell counts
    - Exploratory analyses
- Stool sample (optional)
- Lefitolimod/placebo dosing (always dosed after blood collection)

- 3BNC117/placebo infusion (always dosed after blood collection)
- 10-1074/placebo infusion (always dosed after blood collection)
- Instruct participants to cease ART the following day (Day 15). If regimen requires, appropriate measures to ensure a proper ART withdrawal will have been initiated prior to this date according to regiment (Section 7.7.1.1; Withdrawal of ART).
- If female; pregnancy test (serum/urine) prior to ATI initiation

7.2 The analytical treatment interruption

7.2.1 Visit 5/Day 21 (+/- 3 day)

- Medical History
- Directed physical exam (adverse events, local/systemic reaction)
- Blood collection (8 mL)
    - HIV viral load
    - T cell counts
- Lefitolimod/placebo dosing (always dosed after blood collection)

7.2.2 Visit 6/Day 28 (+/- 3 day)

- Medical History
- Directed physical exam (adverse events, local/systemic reaction)
- Blood collection (4 mL)
    - HIV viral load
- Lefitolimod/placebo dosing (always dosed after blood collection)

7.2.3 Visit 7/Day 35 (+/- 3 day)

- Medical History
- Directed physical exam (adverse events, local/systemic reaction)
- Blood collection (22 mL)
    - Routine biochemistry
    - HIV viral load
    - T cell counts
- Lefitolimod/placebo dosing (always dosed after blood collection)
- 3BNC117/placebo infusion (always dosed after blood collection)
- 10-1074/placebo infusion (always dosed after blood collection)

7.2.4 Visit 8/Day 42 (+/- 3 day)

- Medical History
- Directed physical exam (adverse events, local/systemic reaction)
- Blood collection (4 mL)
    - HIV viral load
- Lefitolimod/placebo dosing (always dosed after blood collection)

7.2.5 Visit 9/Day 49 (+/- 3 day)

- Medical History
- Directed physical exam (adverse events, local/systemic reaction)
- Blood collection (8 mL)
    - HIV viral load
    - T cell counts
- Stool sample (optional)
- Lefitolimod/placebo dosing (always dosed after blood collection)

7.2.6 Visit 10a/Day 56 (+/- 3 days)

- Medical History
- Directed physical exam (adverse events, local/systemic reaction)
- Blood collection (120 mL)

    - Routine biochemistry
    - HIV viral load
    - T cell counts
    - Exploratory analyses

7.2.7 Visit 10b/Day 63 (+/- 3 days)

- Medical History
- Directed physical exam (adverse events, local/systemic reaction)
- Blood collection (4 mL)
    - HIV viral load

7.2.8 Visit 10c/Day 77 (+/- 3 days)

- Medical History
- Directed physical exam (adverse events, local/systemic reaction)
- Blood collection (22 mL)
    - Routine biochemistry
    - HIV viral load
    - T cell counts

7.2.9 Visit 10d/Day 91 (+/- 3 days)

- Medical History
- Directed physical exam (adverse events, local/systemic reaction)
- Blood collection (4 mL)
    - HIV viral load

7.2.10 Visit 10e/Day 105 (+/- 3 days)

- Medical History
- Directed physical exam (adverse events, local/systemic reaction)
- Blood collection (120 mL)
    - Routine biochemistry
    - HIV viral load
    - T cell counts
    - Exploratory analyses
    - If female; pregnancy test (serum/urine)

7.2.11 Visit 10f/Day 119 (+/- 3 days)

- Medical History
- Directed physical exam (adverse events, local/systemic reaction)
- Blood collection (4 mL)
    - HIV viral load

7.2.12 Visit 10g/Day 133 (+/- 3 days)

- Medical History
- Directed physical exam (adverse events, local/systemic reaction)
- Blood collection (22 mL)
    - Routine biochemistry
    - HIV viral load
    - T cell counts

7.2.13 Visit 10h/Day 147 (+/- 3 days)

- Medical History
- Directed physical exam (adverse events, local/systemic reaction)
- Blood collection (4 mL)
    - HIV viral load

7.2.14 Visit 10i/Day 161 (+/- 3 days)

- Medical History
- Directed physical exam (adverse events, local/systemic reaction)
- Blood collection (22 mL)
  - o Routine Biochemistry
  - o HIV viral load
  - o T cell counts

7.2.15 Visit 10j/Day 175 (+/- 3 days)

- Medical History
- Directed physical exam (adverse events, local/systemic reaction)
- Blood collection (4 mL)
  - o HIV viral load

7.3 Resumption of ART during ATI:

7.3.1 Visit 11/Day 189 (+/- 3 days) or day of resumption of ART during ATI

- Medical History; vital signs; height/weight
- Directed physical exam (adverse events, local/systemic reaction)
- Blood collection (120 mL)
  - o Routine biochemistry
  - o Plasma virus and proviral sensitivity to bNAbs
  - o HIV viral load
  - o T cell counts
  - o Exploratory analyses

- The subject will be followed every 4 weeks until the HIV-1 RNA is <50 copies/mL using standard assays.
- Subjects whom have not met criteria to re-initiate ART and have undetectable levels of HIV-1 RNA (by standard plasma HIV RNA assay quantification methods, e.g. < 20 copies/mL by the Cobas Taqman assay) by the end of analytical treatment interruption will be asked to continue ART interruption until ART resumption criteria are met. Participants whom have not met criteria to re-initiate ART but have low detectable levels of HIV-1 RNA (by standard plasma HIV RNA assay quantification methods, e.g. 20-1,000 copies/mL by the Cobas Taqman assay) will be offered to re-initiate ART but may continue off ART if they wish. In cases where the analytical treatment interruption is extended, ART will be re-initiated according to the restart criteria outlined in 7.8.1. These study subjects will be followed by study investigators for up to 3 years off ART with a recommended schedule of monthly blood draws (HIV viral load) decided on individual basis between participant and study investigators before being referred back to their local department. Participants who have not met the requirements for re-initiating ART at visit 11 will be asked to supply a leukapheresis sample for additional exploratory analyses to gain insights into the molecular mechanisms of their ability to control HIV viremia in the absence of antiretroviral therapy.

7.4 Re-suppression following end of ATI

7.4.1 Visit 12, 4-8 weeks following resuppresion of viremia (as defined in 7.3.1)

- Blood collection (80 mL)
  - o HIV viral load
  - o CD4 T cell count
  - o Exploratory analyses

7.5 End of trial

End of trial is defined as Visit 12 *or* 6 months after Visit 11, whichever comes first. Afterwards the subject will no longer be part of this protocol. The subject will resume routine control on individual basis following standard guidelines.

7.6 Blood sampling

The blood samples (sampling plan in Table 4) will be analyzed according to the following:

1. Routine biochemistry (safety) includes hematology parameters (hemoglobin, total and differential leukocyte count, platelet count), glucose, ALAT, bilirubin, creatinine, sodium, potassium and albumin (performed at the local reference lab). Laboratory values will be done at screening, visit 2 and at visit 4 and 7, so the results can be checked before infusion of IMPs. During the ATI it will be done every fourth week.
2. CD4/8+ T cell counts will be performed at the local reference lab at least every other week during treatment with the IMP's and at least every fourth week for the rest of the ATI.
3. Plasma HIV-1 RNA will be measured using Cobas TaqMan (Roche) or other validated assay(s) at the local reference lab at every visit.
4. Exploratory analyses on peripheral blood are detailed in Section 8.2.3 (Potential Exploratory Analyses) and will potentially be done at baseline, before ATI, at visit 10a, 10e, 11 and 12.

7.6.1 Blood volume per analyses

1. Routine clinical blood biochemistry: amount 14 mL
2. CD4/8+ T cell count: amount 4 mL
3. Plasma HIV-1 RNA: amount 4 mL
4. Hepatitis status: amount 11 mL
5. Pre-screening of viral bnAb sensitivity: amount 30 mL
6. 3BNC117 sensitivity: amount 18 mL
7. Remainder of blood collected on Visits 2, 4, 10a, 10e, 11 and 12 will be analyzed fresh (e.g. dendritic cell activation by flow cytometry) or preserved for exploratory analyses (cell-associated viral DNA levels, T cell function). Blood will be separated into components (e.g. plasma, PBMC, or CD4+ T cells), then separate components will be properly stored in aliquots for subsequent analyses as described in Section 8.2.3 (Potential Exploratory Analyses).

7.6.2 Total amount of blood per visit

Total blood sample volumes for each visit are detailed per visit in Section 7.1-7.3 as well as in Table 4. The total amount of blood scheduled to be collected during the entire 28 weeks of the study is 866 mL. For the screening visit the total amount of blood is 70 mL. For the re-suppresion visit after end of ATI (visit 12), the total amount of blood is 80 mL.

Blood volumes and collection dates have been structured to ensure that no 8-week interval has greater than 500 mL of blood collected. The maximum amount of blood scheduled to be collected within an 8-week interval is 420 mL during the first 8 weeks following the first dosing with an investigational medical product. At visit 11, a major blood draw (120 mL) is scheduled, but if visit 11 will be within 7 days of another major blood draw (120 mL), the blood draw at visit 11 will not be taken.

7.7 Stool sampling (optional)

As an optional part of the study, the study participants are asked to provide stool samples at three timepoints (visit 2, 4 and 9). The samples should be collected with the collection kit; Stool Nucleic Acid Collection and Preservation System (Product #63700) from Norgen Biotech corporation (Thorold, Canada) and should be collected at home the day before the above mentioned visits. It is advised that the study participants at inclusion receives the stool collection kits and that the participant is reminded about the stool collection 24 hours before the visits.

7.7.1 Stool sample analysis

The purpose of collecting stool samples is to monitor and characterize changes in the microbiome relative to changes in the viral reservoir and host immune responses. This is an exploratory component of the trial and therefore sample size determinations cannot be made accurately. Stool samples collected during the study will be analyzed in Spain and USA. In agreement with the general data protection regulation (GDPR), all personal information will be safeguarded by pseudonymization.

7.8 Safety monitoring

Safety will be monitored by vital signs, clinical laboratory tests, history and physical examinations if needed and the rate and severity of AE.

If indicated in the opinion of the investigator, a physical examination will be performed prior to and after completed dosing (subcutaneous for lefitolimod; intravenous for 3BNC117 and 10-1074). Routine biochemistry (safety) will be performed prior to the first dosing of lefitolimod and prior to 3BNC117 and 10-1074 dosings. Dosing will be postponed in case of unacceptable laboratory values prior to dosing, and laboratory tests may be repeated, as clinically indicated, to obtain acceptable values before withdraw from the study:

- Hepatic transaminases (AST or ALT) ≥3 x upper limit of normal (ULN)
- Serum total bilirubin ≥3 ULN
- Estimated glomerular filtration rate (eGFR) ≤50 mL/min (based on serum creatinine or other appropriate validated markers)
- Platelet count ≤100 $x10^9$/L
- Absolute neutrophil count ≤0.75$x10^9$/L
- Serum potassium or sodium outside ≥1.3 ULN/LLN

Table 4. Overview of study visits, follow-up and safety monitoring

Line 1	Visit	1	2	3	4		5	6	7	8	9	10a	10b	10c	10d	10e	10f	10g	10h	10i	10j	11	12
Line 2		Screening	Dosing and Sampling			Initiate ATI	On study medication - ATI monitoring					Off study medication - ATI monitoring										End of study	Follow-up
Line 3	Week	Weeks -20 to -2	Week 1	Week 2	Week 3	Week 3	Week 4	Week 5	Week 6	Week 7	Week 8	Week 9	Week 10	Week 12	Week 14	Week 16	Week 18	Week 20	Week 22	Week 24	Week 26	Weeks 4 to 28	N.A.
Line 4	Study Day	Days -140 to -15	Day 0	Day 7 (+/-3 day)	Day 14 (+/-3 day)	Day 15 (+/-1 day)	Day 21 (+/-3 day)	Day 28 (+/-3 day)	Day 35 (+/-3 day)	Day 42 (+/-3 day)	Day 49 (+/-3 day)	Day 56 (+/-3 day)	Day 63 (+/-3 day)	Day 77 (+/-3 day)	Day 91 (+/-3 day)	Day 105 (+/-3 day)	Day 119 (+/-3 day)	Day 133 (+/-3 day)	Day 147 (+/-3 day)	Day 161 (+/-3 day)	Day 175 (+/-3 day)	Days 50 to 189 (+/- 2 days)	4-8 weeks following viral resuppression
Line 5	Dosing of MGN1703/placebo		x	x	x		x	x	x	x	x												
Line 6	Dosing of 3BNC117/placebo				x				x														
Line 7	Dosing of 10-1074/placebo				x				x														
Line 8	Informed Consent	x																					
Line 9	Inclusion / Exclusion Criteria	x																					
Line 10	Demography / Medical History / Concurrent Illnesses	x																					
Line 11	Medication History	x	x	x	x		x	x	x	x	x	x	x	x	x	x	x	x	x	x	x	x	
Line 12	Vital signs (temperature)	x																				x	
Line 13	Vital signs (systolic/diastolic BP PR RR)	x																				x	
Line 14	ECG	x																					
Line 15	Weight / Height	x																				x	
Line 16	Pregnancy (serum / urine in women)	x			x											x							
Line 17	Pregnancy counselling (for women)	x																					
Line 18	Contraception counseling (all)	x																					
Line 19	Local or systemic reaction to investigational medicines			x	x		x	x	x	x	x	x	x	x	x	x	x	x	x	x	x	x	
Line 20	Adverse events			x	x		x	x	x	x	x	x	x	x	x	x	x	x	x	x	x	x	
Line 21	General physical	x																				x	
Line 22	Directed physical		x	x	x		x	x	x	x	x	x	x	x	x	x	x	x	x	x	x		
Line 23	Targeted peripheral blood collection volume (study total = 818 mL)	70	250	4	120		8	4	22	4	8	120	4	22	4	120	4	22	4	22	4	120	80
Line 24	Routine clinical blood biochemistry	x	x		x				x			x		x		x		x		x		x	
Line 25	Hepatitis status	x																					
Line 26	Genotyping HLA Class I and IL-28		x																				
Line 27	Anti-bNAb response (sample taken on day of ART resumption)																					x	
Line 28	Characterizing proviral suceptibility to bNAb neutralization	x																				(x)	(x)
Line 29	Characterizing plasma virus suceptibility to bNAb neutralization		(x)																			(x)	(x)
Line 30	Clinical plasma HIV-1 viral load (e.g. Cobas TaqMan)	x	x	x	x		x	x	x	x	x	x	x	x	x	x	x	x	x	x	x	x	x
Line 31	Cell-associated HIV-1 DNA (total)		x		x							x				x						x	
Line 32	Absolute CD4 counts	x	x		x		x		x		x	x		x		x		x		x		x	x
Line 33	Absolute CD8 counts	x	x		x		x		x		x	x		x		x		x		x		x	
Line 34	DC, monocyte, NK cell activation (flow on fresh blood)		x		x							x				x						x	
Line 35	Other exploratory immunological analyses (e.g. NK, B and/or T cell function, anti-HIV tetramer staining)		x		x							x				x						x	x
Line 36	Other exploratory virological analyses (e.g. qVOA, TILDA, CA-US HIV-1 RNA)		x		x							x				x						x	x
Line 37	Other Research Assays		x		x							x				x						x	x

7.8.1 Analytical ART Interruption (starting on Day 15)

Upon completion of Visit 4 on Day 14, subjects will be instructed to discontinue ART starting the next day and to continue off ART for 24 weeks. The analytical treatment interruption is being performed in order to evaluate the effect of the IMPs on virological control in the absence of ART. Enrollment into the analytical treatment interruption is planned for all participants.

The following criteria require resumption of ART during analytical treatment interruption:

- CD4+ T cell count <350 cells/mm^3 or >50% decline compared to baseline (defined as the mean value of the CD4 cell counts on v2 (day 0) and v4 (day 14)). In the case of a CD4+ T cell count <350 cells/mm^3 or a >50% decline from baseline, an additional measurement should be made ~7 days later to confirm before resumption of ART . If this does not comply with the planned visits, an unscheduled visit should be planned.
- Sustained (≧4 weeks) plasma HIV-1 RNA ≧1000 copies/mL or confirmed plasma HIV-1 RNA ＞100,000 copies/mL.
- Any HIV-1 related symptoms (e.g. acute retroviral syndrome or opportunistic infection).
- The subject becomes pregnant
- Subject request
- If continuing the ART interruption, in the opinion of the Sponsor or Investigator, poses an unacceptable risk to the subject

If a subject requires resumption of ART, the subject will be followed at 4 week intervals until the HIV-1 RNA is <50 copies/mL using standard clinical assays (e.g. Cobas Taqman). Afterwards the subject will resume routine treatment and control following standard treatment guidelines.

4-8 weeks after plasma HIV-1 RNA is re-suppressed (<50 copies/mL), subjects will be asked to donate 80 mL blood for exploratory research analyses (detailed in Section 8.2.3) to evaluate the durability of any investigational product-related effects on these parameters.

For scientific purposes, the analytical treatment interruption for each subject is concluded upon resumption of ART or Day 189 (end of 24-week analytical treatment interruption), whichever comes first.

Subjects whom have not met criteria to re-initiate ART and have undetectable levels of HIV-1 RNA by standard plasma HIV RNA assay quantification methods (e.g. < 20 copies/mL by the Cobas TaqMan assay) by the end of analytical treatment interruption will be asked to continue ART interruption until ART resumption criteria are met. Participants whom have not met criteria to re-initiate ART but have low detectable levels of HIV-1 RNA (by standard plasma HIV RNA assay quantification methods, e.g. 20-1,000 copies/mL by the Cobas Taqman assay) will be offered to re-initiate ART but may continue off ART if they wish. In cases where the analytical treatment interruption is extended, ART will be re-initiated according to the restart criteria outlined in 7.6.1.
These study subjects will be followed by study investigators for up to 3 years off ART with a recommended schedule of monthly blood draws (HIV viral load) decided on individual basis between participant and study investigators before being referred back to their local department. Participants who have not met the requirements for re-initiating ART at visit 11 will be asked to supply a leukapheresis sample for additional exploratory analyses to gain insights into the molecular mechanisms of their ability to control HIV viremia in the absence of antiretroviral therapy.

7.8.2 Lefitolimod

As described in Section 2.1.5.1 (lefitolimod), the most frequently occurring AE collected in the investigator brochure for lefitolimod dosing are:

- Mild injection site reaction (e.g. redness); lasting less than 48 hours.
- Transient Neutropenia; lasting less than one week
- Mild to moderate flu like symptoms (such as fever, chills, headache, myalgia and fatigue); lasting less than 48 hours.

Areas of safety considerations are:

- Injection Site Reaction
  - The local reactions reported in clinical and pre-clinical studies, including mild redness, itching, induration, ecchymosis, pain at the injection sites etc. are characteristic to the compounds with immuno-modulating properties. The clinical and pre-clinical experience with lefitolimod shows that all local symptoms were well manageable and did not cause drug discontinuation. The subcutaneous administrations were generally well tolerated. Huge data collection of local tolerability and follow-up data from the MGN1703-C02 study revealed no major findings. Assessment of local tolerability failed to raise any concern about the local tolerability of the treatment with lefitolimod.

- Transient neutropenia
  - Transient neutropenia is sometimes observed in the first 24 to 72 hours after administration of a TLR9 agonist as a class effect; this is likely due to redistribution of leukocytes in the body rather than a cytotoxic effect (70). The risk remains to be monitored through routine blood parameters testing. If any laboratory abnormalities in neutrophil count occur during the study, the participant will be monitored with increased frequency until the abnormality has resolved. If the neutrophil count drops below 0.75 x 10^9/L the dose of lefitolimod will be reduced until the neutrophil count is ≥1.0 x 10^9/L.

- Pregnancy
  - Pregnancy, although not itself a serious adverse event, will also be reported on a Serious Adverse Event form and be followed up to determine outcome, including spontaneous or voluntary termination, details of birth, and the presence or absence of any birth defects, congenital abnormalities or maternal and/or newborn complications.

- Autoimmunity
  - As a precaution patients with pre-existing autoimmune or antibody-mediated diseases should not be administered lefitolimod because this drug, being a (partly double-stranded) nucleic acid molecule, may be recognized by anti-double stranded DNA antibodies. The monitoring of autoimmunity by several biomarkers was conducted in the Phase 1 clinical study MGN1703-C01, and Phase 2 clinical study MGN1703-C02 [Berlin, Germany; MGN1703 Investigator's Brochure Version No.: 11; release date: December 13, 2017]. Specifically, the following tests were performed: (i) serum test for antibodies directed against double-stranded DNA (anti-ds DNA), (ii) serum test for anti-nuclear antibodies, and (iii) serum test for the development of so-called rheumatoid factor, i.e. antibodies directed against the Fc region of antibodies.
  - For the Phase 1 clinical study MGN1703-C01 individual changes in autoimmunity and immunogenicity parameters were slight. There was no indication for any drug-related changes in antibodies against double-stranded DNA, antinuclear antibody and rheumatoid factor. No signs for any autoimmunity were observed.
  - In the Phase 2 clinical study MGN1703-C02 only two laboratory-related AE occurred and were considered possibly related to the study treatment: both were 'antinuclear antibody increased'. The patients with increased anti-nuclear antibody values were analyzed, but no difference between the groups was found.
  - One patient in the ongoing compassionate use program CU1 subsequent to the Phase 2 clinical study MGN1703-C02 developed a serious unexpected Grade 3 AE "membranous glomerulonephritis" leading to treatment stop. Biopsy revealed PLA2RAK and THSD7A positive membranous nephropathy. This supports the autoimmune nature of this case. However, the value of this finding is limited; to distinguish from the background frequency of idiopathic membraneous nephropathy, which is linked to PLA2RAK in approximately 70% of cases of THSD7A in approximately 5% of cases. Primary membranous nephropathy has its peak incidence in the age group of the patient and accounts for approximately 75% of all membranous nephropathy cases. Lefitolimod treatment has been reported as a potential cause ("possibly" related) for the event.

7.8.3 3BNC117

3BNC117 has now been administrated to at least 160 individuals and was generally safe and well tolerated in all doses tested ranging from 1 to 30 mg/kg. To date, 8 ART-treated HIV-1 infected individuals have received two 3BNC117 infusions, administered 3 weeks apart. 3BNC117 have in two clinical trials been administered with 10-1074, but this is the first time 3BNC117 will be administered in combination with lefitolimod. Areas of safety considerations are included in the following routine clinical blood biochemistry (safety): Metabolic panel of sodium, potassium, glucose, creatinine, ALT, bilirubin, white blood cell count, hemoglobin and platelet count.

- Immunologic symptoms such as listed below are possible with administration of a mAb and will be considered adverse events of interest. Potential allergic-type reactions during and immediately following the administration of 3BNC117 will be carefully monitored:
    - Constitutional symptoms, such as fever, rigors/chills
    - Injection site reaction/extravasation changes, pruritus, urticaria
    - Serum sickness like syndromes as evidenced by fever, rash, arthralgia, arthritis, nephritis
    - Deposition of immune complexes in the kidneys leading to renal insufficiency
    - Adult Respiratory Distress Syndrome, bronchospasm/wheezing, anaphylaxis
    - Cytokine release syndrome/ acute infusion reaction
- 3BNC117-resistant viral strains
    - Following administration of 3BNC117. Development of 3BNC117 resistance might limit the future use of 3BNC117 by the study subject, if this monoclonal antibody is licensed for clinical use.
- Conjunctival toxicity
    - In the cross-reactivity study in human tissues, 3BNC117 was found to bind to cells in the conjunctival recesses. It is possible that this binding could lead to conjunctival toxicity. However, when rats and non-human primates were administered 3BNC117, conjunctival toxicity was not observed. At least 117 participants have received 3BNC117, and 14 participants reported mild ophthalmic complaints (such as pruritus, conjunctival erythema, and increased lacrimation) during study follow-up. In all instances symptoms resolved without specific treatment and ophthalmologic evaluations 5 months after 3BNC117 administration did not show changes from baseline

7.8.4 10-1074

10-1074 has now been administrated to 81 individuals and was generally safe and well tolerated in all doses tested ranging from 3 to 30 mg/kg. To date, at least 59 individuals have been administered one dose of 30 mg/kg (36) (New York City, NY USA; 3BNC117 Investigator's Brochure Edition No.: 5; release date: May 15, 2018) (New York City, NY USA; 10-1074 Investigator's Brochure Edition No.: 3.0; release date: August 10, 2017). 10-1074 have in two clinical trials been administered with 3BNC117, but this is the first time 10-1074 will be administered in combination with lefitolimod. Areas of safety considerations are included in the following routine clinical blood biochemistry (safety): Metabolic panel of sodium, potassium, glucose, creatinine, ALT, bilirubin, white blood cell count, hemoglobin and platelet count.

- Immunologic symptoms such as listed below are possible with administration of a mAb and will be considered adverse events of interest. Potential allergic-type reactions during and immediately following the administration of 10-1074 will be carefully monitored:
    - Constitutional symptoms, such as fever, rigors/chills
    - Injection site reaction/extravasation changes, pruritus, urticaria
    - Serum sickness like syndromes as evidenced by fever, rash, arthralgia, arthritis, nephritis
    - Deposition of immune complexes in the kidneys leading to renal insufficiency
    - Adult Respiratory Distress Syndrome, bronchospasm/wheezing, anaphylaxis
    - Cytokine release syndrome/ acute infusion reaction
- 10-1074-resistant viral strains
    - Following administration of 10-1074. Development of 10-1074 resistance might limit the future use of 10-1074 by the study subject, if this monoclonal antibody is licensed for clinical use.

7.9 Unscheduled Visits or Telephone Contacts

An unscheduled telephone contact or visit may be scheduled for further assessment of any AE or if a blood samples needs to be repeated. A medically qualified member of the study staff will assess the symptom that prompted the visit. Findings will be recorded in the CRF.

7.9.1.1 Withdrawal of ART

The prolonged intracellular elimination time of tenofovir provides well-matched pharmacokinetic profiles for the most common ART regimen and would presumably not result in "functional monotherapy" after simultaneous withdrawal. However, to rule out any risk of resistance development the following principles of ART withdrawal have been decided upon:

- Participants on non-nucleoside reverse-transcriptase inhibitors will switch to an integrase inhibitor at least two weeks prior to receiving any study medication, thus a total of four weeks before the ATI.

8 EFFICACY ASSESSMENTS

Every effort will be made to ensure that the protocol required tests and procedures are completed as described. When a protocol-required test cannot be performed as specified, the Investigator will document the reason for this and any corrective or preventive actions that has been taken to ensure that protocol requirements are adhered to as soon as possible. In these cases, the Investigator will take all steps necessary to ensure the safety and well-being of the subject. Any subject who receives at least one dose of the IMP(s) will be included in the efficacy assessment.

For a detailed specification of study endpoints refer to Section 3.2 (Endpoints).

8.1 Primary Endpoint

8.1.1 Analytical Treatment Interruption

Plasma HIV-1 RNA levels will be monitored at each study visit and CD4+ T cell counts will be monitored at least every other week during administration of the IMPs and at least every fourth week during the rest of the study period. ART will be restarted after sustained (≧4 weeks) plasma HIV-1 RNA ≧1000 copies/mL, confirmed plasma HIV-1 RNA >100,000 copies/mL, if CD4+ is found to be < 350 cells/μL or >50% declined compared to baseline (defined as the mean value of the CD4 cell counts on v2 (day 0) and v4 (day 14)) on two consecutive measurements, if the participant experience any HIV-1 related symptoms (e.g. acute retroviral syndrome or opportunistic infection), the participant becomes pregnant, or if otherwise clinically indicated.

8.2 Secondary Endpoints

8.2.1 Safety and Tolerability assessment

Subject who receives at least one dose of the IMP(s) will be included in the evaluation for safety. Safety assessment will be done by recording of all participant-reported AE and SAE. For each AE/SAE the relationship to the IMP(s) will be evaluated and the severity graded according to the CTCAE v5.0.

8.2.2 Plasma HIV-1 RNA

Quantitative plasma HIV-1 RNA will be measured by the Cobas Taqman assay (Roche), which is a routine clinical assay with a sensitivity of 20 HIV-1 RNA copies/mL

8.2.3 Potential Exploratory Analyses

- Plasma from participants may be evaluated for the presence of soluble markers of inflammation (e.g. IFN-γ, IFN-α, IP-10, IL-2, TNF-α, IL-6, IL-12p40 and IL-8). Plasma protein concentrations may be determined using multiplex technology (e.g. Mesoscale) according to manufacturer's recommendations.
- CD4+ T cells may be isolated from thawed PBMCs using a CD4+ T cell isolation kit and magnetic-activated cell sorting columns. Cellular DNA may be extracted using a Qiagen DNA Blood Midi Kit. Total HIV-1 DNA quantifications may be assessed using the QX100™ Droplet Digital™ PCR system (Bio-Rad). For quantification of integrated HIV-1 DNA, CD4+ T cells may be isolated from

thawed PBMCs using a CD4+ T cell isolation kit and magnetic-activated cell sorting columns. For integrated HIV-1 DNA quantification, a two-step real-time PCR targeting Alu-long terminal repeat can be used with primers and probes as previously described by Brussel and Sonigo (71).

- T cell polyfunctionality may be determined by flow cytometry following an ICS. Cryopreserved PBMCs may be stimulated with an overlapping gag-peptide pool.
- A limiting dilution viral outgrowth assay may be used to quantitate the size of the latent HIV-1 reservoir at baseline and prior to analytical treatment interruption, as we have previously done (16, 17, 72). Virus positive cultures can be sequenced and used to assess virus phylogeny. We may also use the TILDA assay to quantitate transcriptionally competent HIV as we have previously done (17).
- Absolute cell counts for relevant lymphocytes (e.g. CD4+ and CD8+ T cells) will be assessed at each site's clinical immunology laboratory according to their standard operating procedures
- Expression of activation markers on peripheral blood NK and T cells may be performed using standard flow cytometry methods on a flow cytometer. Cryopreserved PBMCs can be thawed and stained with Live-dead dye and lineage- or function-specific antibodies (e.g. NK cell activation profile; T cell activation profile: and DC [lineage negative, HLA-DR positive] activation profile). Only live, singlet cells will be included in the analysis. Lymphocytes will be identified by size and granularity. Population gating will be based upon appropriate expression of lineage specific markers. Gates for activation marker positivity will be determined using isotope control antibodies.
- Post hoc, virus from participants at baseline (cultured ex-vivo) and plasma viruses at viral rebound may be screened for sensitivity to 3BNC117 and 10-1074 neutralization. Further, plasma from a subset of participants may be screened at baseline and at viral rebound using a broader panel of tier-2 viruses to investigate changes in host humoral immunity. Plasma bNAb concentrations may be evaluated as predictors of treatment response.
- The optional stool samples may be used to perform microbiome analysis. The composition of an individual's microbiome has previously been associated with response to immunotherapy.

9 SAFETY ASSESSMENTS

9.1 Protocol Safety Review Team

The Protocol Safety Review Team (PSRT) will include the available site PIs and co-investigators. Additional members could include senior clinical research nursing staff and site staff. The PSRT will meet on a monthly basis to review any adverse events; minutes will be recorded. For any AE Grade 3 or above deemed possibly, probably or definitely related to lefitolimod, 3BNC117 and/or 10-1074, the site PI will notify the PSRT within 24 hours and the PSRT will convene within 1 business day to review these AEs. The PSRT will decide by consensus whether AEs should also be reviewed by the Study Monitoring Comittee.

9.2 Safety Monitoring Committee (SMC)

A SMC will be established to monitor the study. The charter of the SMC is to provide an ongoing assessment of volunteer safety during the conduct of the study. The SMC will consist of three independent individuals who have no relationship to the Principal Investigators and Co-Investigators involved in the trial. No member of the SMC will have any direct responsibility for the clinical care of trial volunteers. No representative of Gilead Sciences Inc. ~~Mologen~~ or the Rockefeller University, or their designees, may be a member of the SMC. However, the SMC may invite the principal investigators (PI) or designee and a Gilead Sciences Inc. ~~Mologen~~ and/or Rockefeller University representative to an open session of a SMC meeting to provide information on study conduct, present data, or to respond to the members' questions.

At least two members of the SMC must be in attendance (phone, video, or in-person meetings) to constitute a quorum for an SMC meeting. SMC members may also review and comment by email, if scheduling cannot be worked out in a timely manner. One member of the SMC will be appointed as chair of the committee. The SMC chair (or his/her alternate) will be responsible for summarizing and communicating in writing SMC acknowledgments and recommendations to the PI within 5 business days following each SMC meeting and/or review.

All available safety data will be reviewed by the SMC four weeks after the first eight participants started the ATI, and every 6 months thereafter. The study will not pause, but following each review, SMC member(s) will make a recommendation to the principal investigator(s) regarding the continuation of the trial.

SAEs and unanticipated adverse events will be reported to the SMC within 2 working days of the site becoming aware of the event. If there is one at least grade 4 SAE judged as possibly, probably or definitely related to the administration of lefitolimod, 3BNC117 and/or 10-1074 by the principal investigator or designee, no additional enrollment will take place pending a review by at least two members of the SMC. Following this review, the SMC member(s) will make a recommendation to the principal investigator regarding the continuation of the trial.

All updated versions of the protocol will be provided to the SMC members. For SMC meetings, the study team will provide the SMC with updated records of all adverse events (AEs) of a grade 2 or greater toxicity.

The SMC will provide a written report to the sponsor at Aarhus Univeristy Hospital and the site PIs after each evaluation. The PIs will, in turn, distribute these reports to the study team, the local Institutional review boards and the regulatory authorities (if needed).

9.3 Monitoring

Safety monitoring (Section 7.6) at all sites will be conducted by the study investigators and by an external monitor. An external SMC will review SAE's and Unanticipated AEs and will be available to the study investigators for consultation. The GCP unit at Aalborg and Aarhus University Hospitals will conduct the initial review of the proposed study and will regularly review the trial's progress throughout the course of the study.

9.4 Procedure for premature unblinding

As a safety consideration for the participants, it is important that the participants study arm designation can be revealed at all times, if necessary. When a participant enters the study, and is randomized to one of the four study arms, the information about the study arm designation will be printed out and placed in a sealed envelope at Department of Infectious Diseases, Aarhus University Hospital. There will only be information about one participant in each envelope. If needed, study arm designation for a participant can be achieved by contacting study personnel (Time: 8-15, Tel. 24 77 79 95) or the doctor on duty at Department of Infectious diseases, Aarhus University Hospital (Time: 24 hours a day, Tel. 78 45 28 11 / 40 45 96 77).

9.5 Adverse Events (AE)

An AE is any untoward medical occurrence in a patient or clinical investigation subject administered a pharmaceutical product that does not necessarily have a causal relationship with this treatment. An AE can therefore be any unfavorable and unintended sign (including an abnormal laboratory finding – Section 7.6; Safety Monitoring), symptom, or disease temporally associated with the use of the IMP(s), whether or not causally linked to the IMP(s).

Abnormal laboratory values or test results constitute AE only if they induce clinical signs or symptoms, are considered clinically significant, or require intervention.

In addition, all cases of drug-drug interaction, pregnancy (with or without outcome), paternal exposure, lactation, lack of efficacy, overdose, drug abuse and misuse, drug maladministration or accidental exposure and dispensing errors are collected and data based even if no adverse event has been reported.

All AE will be reported in accordance with the principles of GCP and the latest requirements of the Medicines for Human Use (Clinical Trials) Regulations.

All AEs must be scored according to the CTCAE (v5.0) and recorded on the AE form in the CRF with the following information:

1. The severity grade (mild, moderate, severe)
2. Its relationship to the IMP(s) (suspected/not suspected)
3. Its duration (start and end dates or if continuing at final exam)
4. Whether it constitutes a serious adverse event (SAE)
5. Action taken with the IMP(s)
6. Outcome

9.6 Serious Adverse Events (SAE)

An SAE is any untoward medical occurrence that at any dose:

- Results in death
- Is life-threatening
- Results in persistent or significant disability/incapacity
- Constitutes a congenital abnormality/birth defect
- Requires inpatient hospitalization or prolongation of existing hospitalization, *unless hospitalization is for:*
    - o Routine treatment or monitoring of the studied indication, not associated with any deterioration in condition
    - o Elective or pre-planned treatment for a pre-existing condition that is unrelated to the indication under study and has not worsened since the start of the IMP(s)
    - o Treatment on an emergency outpatient basis for an event not fulfilling any of the definitions of a SAE given above and not resulting in hospital admission
    - o Social reasons and respite care in the absence of any deterioration in the participant's general condition
- Is medically significant (i.e. defined as an event that jeopardizes the participant or may require medical or surgical intervention to prevent one of the outcomes listed above)

Unlike routine safety assessments, SAEs are monitored continuously and have special reporting.

9.6.1 Pregnancies

Pregnancy, although not itself a serious adverse event, will also be reported on a SAE form and be followed up to determine outcome, including spontaneous or voluntary termination, details of birth, and the presence or absence of any birth defects, congenital abnormalities or maternal and/or newborn complications. Pregnancy should be recorded on a Clinical Trial Pregnancy Form and reported by the Investigator to the local Drug Safety Departments at Gilead Sciences Inc. ~~Mologen AG~~ and Rockefeller University.

9.7 Recording of Adverse Events (AE)

At each contact with the subject, the Investigator seeks information on AE by specific questioning and, as appropriate, by examination. Information on all AE will be recorded immediately in the appropriate AE module of the CRF. All clearly related signs, symptoms, and abnormal diagnostic procedures results will be recorded in the source document, though they should be grouped under one diagnosis.

All AEs occurring between the day of IMP dosing initiation (baseline) and ART resumption in the analytical treatment interruption must be recorded. The clinical course of each event will be followed until resolution, stabilization, or until it has been determined that the study treatment or participation is not the cause. SAE that are still ongoing at the end of the observational follow-up phase must be followed up to determine the final outcome. Any SAE that occurs after the study period and is considered to be possibly related to the study treatment or study participation will be recorded and reported immediately.

9.8 Reporting of Serious Adverse Events (SAE)

The SAE will be noted in the CRF. Information about all SAE is collected and recorded on the SAE Report Form. The Investigator must assess the relationship to the IMP(s), complete the SAE Report Form in English, send the completed, signed form electronically to the Sponsor, and the local Drug Safety Department at Gilead Sciences Inc. and Rockefeller (**Figure 10**). The original copy of the SAE Report Form and a confirmation sheet must be at the study site.

Gilead Sciences Inc.
Att: Devi SenGupta
333 Lakeside Drive Bldg 353
Foster City, CA 94404
T: (650) 425-5060
E-mail: Devi.sengupta@gilead.com

The Rockefeller University Hospital, New York NY
Att. Sarah Schlesinger
1230 York Ave
New York, NY 10065
Phone Number: (212) 327-7396
Fax Number: (212) 327-7234

9.9 SUSAR (Suspected Unexpected Serious Adverse Reaction):

If the SAE is not previously documented in the investigator brochure (new occurrence) and is thought related to the IMP(s), it qualifies as a SUSAR (**Figure 10**). Similar, a suspected SAR which is not referred to in the RSI, qualifies as a SUSAR.

The minimum necessary information to be provided at the time of the initial report includes:

- An identifiable patient
- An identifiable medicinal and/or pharmaceutical product
- An identifiable reporter
- A SAE

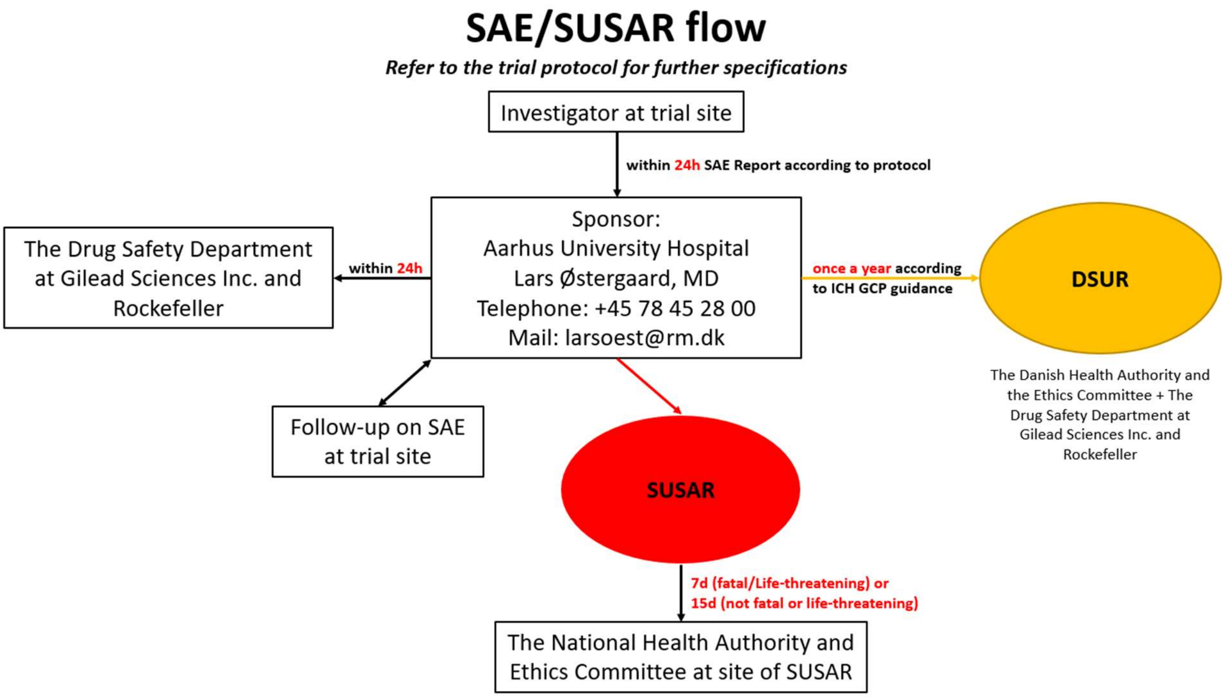

Figure 10. Work flow when reporting SAE and SUSAR.

9.9.1 Reporting of SUSAR to local Health and Medicines Authority and the Ethics Committee

Safety reporting requirements by timeframe for reporting the listed types of events:

- ***Within 7 calendar days***
  - Any study event that is:
    - Serious and
    - Unexpected and
    - Suspected and
    - Fatal or life-threatening

- ***Within 15 calendar days***
  - Any study event that is:
    - Serious and

- Unexpected and
- Suspected

The Sponsor is responsible for submission of reportable SUSAR to the local Health and Medicines Authority and the Ethics Committee for their country according to regulatory requirements.

Danish Medicines Agency
Axel Heides Gade 1
2300 Copenhagen S, Denmark
Tel.: +45 7222 7400

The Norwegian Medicines Agency
Grensesvingen 26
0663 Oslo, Norway
Tel.: +47 2289 7700

SUSARs will be reported by filling in and submitting the Danish Medicines Agency's e-form for reporting of SUSARs (http://laegemiddelstyrelsen.dk/en/licensing/clinical-trials/adverse-reactions/reporting-of-suspected-unexpected-serious-adverse-reactions-susars-seen-in-clinical-trials-e-form).

9.10 Submission of Development Safety Update Report (DSUR)

In addition to the expedited reporting required for SUSAR, Sponsors are required to submit a safety report to the local Health Authority and the Ethics Committee according to local regulations, once a year throughout the clinical trial or on request. The Sponsor will also provide Gilead Sciences Inc. ~~Mologen AG~~ and Rockefeller with a copy of the DSUR at the time of submission to the local Health Authority and the Ethics Committee.

10 STATISTICAL ANALYSES

10.1 Sample Size Determination

The sample size calculation is based on the primary endpoint: time to viral rebound during ATI. We will compare days from date of ART interruption to loss of virological control (defined as sustained (≧4 weeks) plasma HIV-1 RNA ≧1000 copies/mL or confirmed plasma HIV-1 RNA >100,000 copies/mL) in Arm C to Arm D. If loss of virological control occurs, the date of the last measurement of HIV-1 RNA ≧ 1,000 copies/mL or confirmed plasma HIV-1 RNA >100,000 copies/mL will be defined as "date of viral rebound." Using a 2-sample comparison of means with a standard deviation (SD) of 11 days (11, 16), 10 evaluable participants in each of the two arms will have 90% power to detect ≥ 16 days difference in time to viral rebound at a 5% significance level. In addition, arms A and B will be used to explore the role of lefitolimod treatment during the ATI. Arms A and C will be used to explore the role of bNAb treatment during the ATI. Arms B and C will be used to compare bNAb treatment to lefitolimod treatment during the ATI. Arms B and D will be used to explore the additive role of bNAbs to lefitolimod treatment during the ATI. Arms A and D will be used to explore the role of no intervention (placebo) vs the combined intervention (bNAbs+lefitolimod) during the ATI.
To accommodate for dropouts, we aim to enroll 12 participants in each arm. Participants will be enrolled from multiple study sites using competitive enrolment.

10.2 General Data Analyses

The Kaplan-Meier estimator will be used to address the primary outcome variable. We will use the log-rank test or Gehan test to compare time to viral rebound during ATI in arms C and D. The primary analysis will be conducted both as intention-to-treat and per-protocol analyses on the total participant population. We will also use the log-rank test or Gehan test to compare time to viral rebound during ATI between arms A and B; A and C; A and D; B and C; B and D.

The number and percentage of subjects experiencing one or more AEs will be summarized by relationship to study drug and severity. AEs will also be summarized by severity grade and by relationship to study drug according to the CTCAE v5.0 (including infusion-related AEs). Changes will be calculated relative to the values collected at baseline

Secondary analyses may be stratified according to characteristics that may influence either the effect of the interventions and/or the virological/immunological outcome measures. Such characteristics include, but are not limited to, virus sensitivity to 3BNC117 and/or 10-1074 neutralization, time from infection to enrollment, baseline CD4+ T cell count, baseline plasma HIV RNA, duration of VL suppression, reservoir size at baseline, smoking, HLA-type, age, gender. Finally, Cox proportional hazards regression will be used to identify predictors of time to viral rebound

Exploratory analyses will accessed according to the type of data collected. For example paired student t-test or paired Wilcoxon test will be used to determine changes from baseline to the post-analytical treatment interruption time-point in reservoir size for each group..

Fishers exact or chi^2 test will be used to compare the proportion of subjects in each group with detectable viremia during the 8-week treatment period. Functional properties of cytotoxic T cells and NK cells will be correlated to changes in HIV reservoir measures. Flow cytometry data will be evaluated by Boolean partitioning of NK and T cell responses into distinct responding populations (73).

P-values <0.05 will be considered statistically significant. No interim analyses will be done.

11 ACCESS TO SOURCE DATA

The Sponsor and Lead Principal Investigator can – based on approval from the regional Ethics Committee – have access to all source data (Section 4.5; Source Data). During study monitoring, auditing, and/or inspection all relevant source data and study documents will be made available to the study monitor, The Danish Medicines Agency (or country specific medicines agency), The Ethics Committee of the participating country and their collaborators. Personal information about participants from their own countries will similar be made available.

12 STUDY MONITORING AND QUALITY ASSURANCE

12.1 Sponsor and Investigator's Responsibility

It is Sponsor's responsibility to establish and maintain a quality assurance system that guarantees the quality of the study in all aspects. Sponsor can appoint qualified staff Investigators that may assist in the conduct of the study in accordance with the study protocol. All Investigators must be appointed and recorded on the study personnel list in due time before any study related procedures are carried out and must be supplied with the study protocol and all necessary information. Investigators are supervised by Sponsor or the Principal Investigator and act under their responsibility.

Sponsor will notify The Danish Medicines Agency and medicines agencies from involved countries and The Ethics Committees of the participating countries about the completion of the study within 90 days and within 1 year, respectively, and report the findings of the study as early as possible. If the study is prematurely terminated these agencies must be notified within 15 days and the reason for termination must be clarified. Investigator's responsibility is to conduct the clinical trial at the study site, and if the study site consists of a team of individuals, it is the Investigators responsibility to be leader of the team.

12.2 Study Monitoring

The study is monitored by the GCP-Unit at Aalborg and Aarhus University Hospitals. The GCP-Unit and their national and international collaborators will through regular contacts, monitoring visit, telephone contacts, or written correspondence, monitor and assess the conduct of the study and contribute to high ethical, scientific, and legal standards in all aspects of the study. Sponsor, Investigators and study subjects' adherence to protocol requirements will be monitored as well as the handling of irregularities if such occur. During monitoring visits all, but not necessarily limited to, of the following issues will be discussed and

assessed: informed consent, subject recruitment, follow-up, documentation, recording and reporting of AE and SAE, compliance, data quality, and data handling. Monitor will have access to all relevant data material as specified in Section 11 (Access to Source Data).

12.3 Use of Case Report Forms (CRF)

Sponsor is responsible for keeping an updated and accurate electronic CRF (eCRF) intended to correctly register all observations and data related to the study. Recording in the eCRF will usually be done after every visit. Data entry into the eCRF must be done comprehensively and carefully to ensure correct data interpretation. The eCRF have a logging system, which logs; date, time, action in the eCRF and the person responsible for the action. If corrections are introduced, previous text/data will still appear in the eCRF logging system.

13 ETHICAL CONSIDERATIONS

13.1 Study Approvals

Sponsor must have prospective approval of the study protocol, protocol amendments, informed consent documents, recruitment advertisements, and other relevant documents from The Danish Medicines Agency, The Central Denmark Region Committee on Health Research Ethics, and The Danish Data Protection Agency before study initiation. Subsequently the other study sites must obtain approval of the same documents as mentioned above by their respective national regulatory authorities.

13.2 Ethical Conduct of the Study

It is Sponsor and Investigators' responsibility to plan and conduct the study in accordance with the protocol, The Helsinki Declaration (1996 version), guidelines for GCP; International Conference on Harmonization 1996, and national ethical guidelines and law.

13.3 Subject Recruitment, Information and Consent

Written information and advertisements approved by The Ethics Committee and Investigator databases may be used for recruitment purposes (Section 6.3; Consent Procedure). Eligible study subjects will be recruited according to local norms. To direct the information for potential eligible participants, screening on inclusion and exclusion criterias can be done by nurses or doctors at local sites involved in the study. Thus, pass on of information from the patient charts will be allowed before written informed consent has been obtained from the relevant subject. For example, potentially eligible subjects may receive a letter (e.g. mail or e-boks) which contains written information, inviting them to participate in the study. They may be given written and oral study participation details during an office visit or they may respond to an advertisement to receive written study details. Interested subjects can contact a study site to talk with or schedule an appointment with an Investigator to receive additional, possibly attended with a companion. If the subject then wishes more time to consider participation in the study, presence of a companion, or repetition of information, a new appointment will be scheduled for this within the time plan. It is expected that the subject will either reject participation or sign the informed consent within two weeks after both written and oral information has been given.

The Investigator must ensure that each subject is fully informed about the nature and objectives of the study and possible risks associated with participation.

An Investigator will obtain written informed consent from each subject before any study-specific activity is initiated, using a consent form prospectively approved by The Ethics Committee. The study site will retain the original of each subject's consent form; a copy will be given the subject.

13.4 Harms, Risks and Inconveniences

There are certain risks associated with participating in a clinical trial. In this clinical trial the three IMPs; lefitolimod, 3BNC117 and 10-1074 have all been tested in clinical trials earlier.
For lefitolimod, there is now considerable experience with the human safety profile. By end of April 2017, 772 persons have been enrolled in the lefitolimod development program and thereof, 432 patients and 13 healthy volunteers have been treated with lefitolimod (Berlin, Germany; MGN1703 Investigator's Brochure Version No.: 11; release date: December 13, 2017). All safety data is included in the analyses presented in the Investigators Brochure. In addition

to those data, we have published a clinical trial detailing lefitolimod treatment in antiretroviral treated, HIV positive individuals (n=15) where the drug was safe and well-tolerated (1).
3BNC117 has by march 2017 been administered to 160 research participants and it has generally been well tolerated at all dose levels tested (up to 30 mg/kg). Most reported AE's were of graded 1 severity of the 135 AE's that were considered at least possibly related to 3BNC117 infusion.
A total of 81 subjects have received 10-1074 at doses ranging from 3 to 30 mg/kg and there have been no significant adverse events related to 10-1074.
In conclusion, the IMP's have generally been safe and well tolerated, but all IMPs require close monitoring and participation in the study poses the risk of AE's and SAE's.

The safety profile for all IMP's is detailed described in Section 2.1.5 (Safety profile) and all safety data is included in the analyses presented in the Investigators Brochure for each IMP. Safety monitoring is described in Section 7.6 (Safety monitoring).

Since the short- and long term benefits and potential harms of the active study drugs are unknown, some participants will be randomized to receive placebo either with (Arm B and C) or without (arm A) an active compound. Lefitolimod and 3BNC117 / 10-1074 have been administered as monotherapy and these studies have not revealed that is should pose any risk for the participants. The participants who do not receive any active compounds will be as closely monitored as the other participants and will restart ART when the predescribed criteria are met (section 7.6.1). The ATI is used in clinical studies to evaluate full effect of the intervention on the course of HIV infection in the participants. Short and well-monitored ATIs do not seem to pose any risk to the participants.

The study participants might find it inconvenient to attend to visits at the hospital every or every other week during the study.

13.5 Evaluation of Ethical and Scientific Aspects

The study treatment and procedures hold the risks, potential harms, and inconveniences mentioned above in Section 13.4 (Harms, Risks and Inconveniences), but several benefits to the study subjects and the scientific development counterbalance these disadvantages.

The objective of this study is to test and evaluate a specific combination treatment that addresses a fundamental problem in the treatment of HIV-infection: that despite highly effective ART it is still not possible to cure the disease. Consequently, HIV-infected persons are burdened with stigmatization and life-long treatment that may contain unpleasant side effects as well as yet unknown long-term toxicities. In addition, even well-treated HIV-infection still causes chronic immune activation and dysfunction, which is likely the reason for the increased rate of non-AIDS related mortality and morbidity seen among HIV-infected individuals, even in the late ART era (74, 75). In light of our previous results showing latency reversal by lefitolimod to purge and revert HIV-1 latency (1, 33) and the demonstrated ability of 3BNC117 and 10-1074 to reduce viral burden (34-36) , this combination strategy could prove to be an important step towards a treatment strategy with a curing potential.

The ultimate proof of a clinically important reduction of the reservoir is to demonstrate a longer than expected time from stopping ART to viral rebound during an analytical treatment interruption. However, there are several safety issues that need to be addressed when interrupting ART. One is the increased rate of clinical events associated with treatment interruption in individualss with CD4+ T cell count between 250 and 300 cells/mm^3 seen in the SMART study (76). Of note, the absolute rate of events in the interruption arm of that study was very small and not significantly different from the continuous therapy arm in the first 3-4 months after study entry (77). Thus, treatment interruptions of short periods (e.g. 12 weeks) hold little risk from this perspective. To further protect from this risk, it is recommended to re-initiate ART at CD4+ cell-counts <350/mm^3, but can be conducted safely under these circumstances (77, 78). In addition, re-suppression of virus upon treatment resumption has generally been unproblematic without emergence of drug resistance (77, 79, 80).

The scientific value of the study is extensive as there is now an urgent need for clinical studies to support the numerous findings and hypotheses generated by *in vitro, ex vivo* and in non-clinical studies. Careful

evaluation of the efficacy, safety, tolerability, and immunological function of lefitolimod, 3BNC117 and 10-1074, will generate essential new knowledge regarding the possibilities and limitations of achieving cure of HIV-infection through eradication of the HIV-1 reservoir.

Against this background and with the careful monitoring of the study subject's safety, well-being, and privileges, which at any time must receive higher priority than the interests of society and scientific development, this study is found to be ethically justified and scientifically well-balanced.

14 DATA HANDLING AND RECORD KEEPING

14.1 Access to Personal Information

All study subjects will be asked to sign a consent form at study entry allowing Sponsor and Investigators, the GCP units, Ethics Committees, and regulatory authorities to access the source data. All personnel must treat personal data as confidential.
After the informed consent is signed, information on demographics, HIV status, ART regime, medical history and concomitant medicine will be taken from patient journals to get the most accurate informations. The informed consent allows the investigator, sponsor, sponsors representative, study monitor, The Danish Medicines Agency (or country specific medicines agency), The Ethics Committees of the participating countries and their collaborators to have access to the study documents and personal information about participants of their own countries to monitor all data is correct.

14.2 Handling of Personal Information

Sponsor and Investigators including designated personnel must handle and keep all study material and documents as confidential information and take steps to prevent wrongful or premature destruction of these. All personal data are protected in accordance with the relevant national law, the EU General Data Protection Regulation (GDPR) and Data Protection Agency.

Blood samples and subject-specific documents will not contain information that directly identifies the subject, but will be supplied with a study identification code unique for each subject. All study material will be treated in accordance with the national law and Data Protection Agency.

Biological samples including stool samples will be transferred as de-identifiable samples to Roger Paredes, MD, PhD. Infectious Diseases Service & Institut de Recerca de la SIDA - irsiCaixa | Hospital Universitari Germans Trias i Pujol | UVic-UCC & Universitat Autònoma de Barcelona | Ctra. de Canyet s/n, Planta 2a, 08916 Badalona, Catalonia, Spain. Doc Paredes is a world-leading expert in microbiome/HIV interaction analysis and in-depth association analyses of the trial outcomes and changes in the microbiome may reveal novel findings of great importonce to HIV cure research.

Upon completion of all study related analyses all personal data will be rendered anonymous. However, according to GCP-guidelines data will remain personally identifiable for a minimum of 5 years after completion of study procedures defined as last subject's final follow-up contact. Any retained research data will be kept for a period of 20 years.

Data sharing plan: Individual deidentified participant data (including data dictionaries) will be shared following the publication of the primary and secondary endpoints as outlined in this protocol. Data to be shared includes deidentified data points in published, peer-reviewed articles. Additional, related documents will also be available (study protocol, informed consent form, statistical analysis plan). Data will become available following publication with no planned end date. Access to the data sharing will be given to researchers who provide a methodologically sound proposal for any type of analysis and requires IRB/Ethics committee approval (if applicable). Proposal should be addressed to olesoega@rm.dk.

14.3 Research Biobank

The biological material collected during the study will be used for the analyses specified in this protocol and as an integral part of a biomedical research project fulfil the criteria for a research biobank. Therefore, the following information is supplied:

- At each visit (how much and when depicted in Tables 4 as well as Section 7.1-7.3; Study Visits) blood draws will be performed
- The purpose of the research biobank is to store biological material to be used for the analyses described in this protocol (Section 8; Efficacy Assessments)
- Upon completion of all study-related analyses any remaining biological material will be transferred to a biobank for future research conditioned by prior approval by The Danish Data Protection Agency. At enrollment, study subjects will be asked to give consent to storage of their remaining biological material in the biobank for future research (termination 01.01.2038).
- Regarding harms, risks and inconveniences related to collection of samples, please refer to the above section on the subject (Section 13.4; Harms, Risks and Inconveniences)

15 FINANCIAL ISSUES AND INSURANCE

The total costs of the study are estimated to approximately $50,000 per subject amounting to approximately $2,750,000 (including screening failures and unforeseen expenses). The funding will be used for running costs, standard and laboratory analyses, direct and indirect costs of clinical procedures, reimbursement of participants expenses, data analyses and publication/dissemination. Gilead Sciences Inc.~~Mologen AG~~ and Rockefeller University will supply the IMPs free of charge. Subjects enrolled in Denmark are under The Danish Patient Insurance that covers all study subjects as guaranteed by The Danish Act on the Right to Complain and Receive Compensation within the Health Service administered by The Patient Assurance Association.
Study subjects will be reimbursed for transport expenses relating to the study, but will otherwise not receive any financial compensation for participating in the study. Study subjects have the possibility of recovering documented lost earnings, the amount is taxable.
Scientific and technical assisting personnel may be hired directly into the study to carry out designated tasks.

The trial has received a $2,750,000 research grant from Gilead Sciences. The grant will be transferred to an account at Aarhus University Hospital, who administer the grant and is responsible for proper accounting. Official sponsor is Lars Østergaard.

There will be no wage or other benefits neither for the project site or members of the project team or subjects. None of the members of the project are associated with Gilead Sciences and Gilead Sciences will have no access to the person identifiable data.

16 PUBLICATION OF STUDY

16.1 Public Registration of Study

The study will be registered at https://eudract.ema.europa.eu and http://clinicaltrials.gov prior to study start upon receipt of all necessary approvals. A resume and brief outline of the study will be available according to current guidelines.

16.2 Publication of Study Results

Publication of the results of this trial will be governed by requirements for authorship promulgated by the International Committee of Medical Journal Editors http://www.icmje.org. Any presentation, abstract, or manuscript will be made available for review by the pharmaceutical companies providing the IMPs at least thirty (30) days (or, for abstracts, at least five (5) working days) prior to submission.

Results from the study will be published in international peer-reviewed journals, and negative, positive as well as inconclusive test results will be attempted published, the results will also be published on http://clinicaltrials.gov.

A final list of authors cannot be specified at this time, but all contributing researchers who fulfilled the Vancouver criteria for authorship will be included in the final publication.

17 Strategy for Literature Review

A literature search of peer-reviewed publications on the PubMed database was conducted in October 2017. The following search terms were used alone or in combination: “HIV”, “HIV latency”, “HIV reservoir”, “HIV eradication”, “HIV cure”, “MGN1703”, “TLR9 agonist”, “3BNC117”, “10-1074”, and “bNAbs”. Titles and abstracts were reviewed and studies that were judged relevant for the context of the above-mentioned clinical trial were included.

18 Protocol Synopsis

18.1 Title

TITAN – Combining a TLR9 agonist with broadly neutralizing antibodies for reservoir reduction and immunological control of HIV infection: An investigator-initiated randomized, placebo-controlled, phase IIa trial

18.2 Background and study rationale

Although the size of the viral reservoir decreases following initiation of ART, the decay rate is so slow that all individuals regardless of the duration of ART harbor thousands to millions of infected CD4+ T cells containing replication competent proviruses. It has been proposed that reducing the size of the reservoir will increase the chances of achieving ART free HIV remission. Therapeutic interventions that boost NK and CTL-mediated cellular immunity in humans may lead to elimination of cells expressing HIV-1 antigens. In addition to cellular immune responses, broadly neutralizing antibodies (bNAbs) against conserved viral proteins facilitate clearance of cells expressing HIV-1 envelope through FcγR-dependent mechanisms. Thus, there is a compelling rationale that combining the qualities of enhanced cellular immunity with potent and broad HIV-specific humoral immunity will lead to effective killing of cells expressing HIV antigens, which in the presence of a latency reversing agent (e.g. histone deacetylase inhibitor or TLR agonist) may significantly reduce the frequency of latently infected cells.

We are combining three immunotherapeutic interventions, a TLR9 agonist and two bNAbs against HIV-1 envelope in a randomized clinical trial. As outlined below, this treatment combination may not only have an immediate effect on the frequency of latently infected cells, but may also lead to the development of long-lasting immune surveillance mechanisms. While this treatment concept is new in the HIV field, cancer studies have provided proof-of-concept that the effects of TLR9 agonists can synergize with the effects of monoclonal antibodies such as rituximab and cetuximab leading to increased elimination of target cells via FcγR dependent mechanisms.

Lefitolimod stimulates TLR9 in human pDCs and B cells. Upon TLR9 stimulation, activated pDCs release interferons and migrate to lymphoid tissue where they facilitate B cell maturation, germinal center formation, enhanced cross-priming, and CD8 T cell immunity. TLR9 agonists also activate NK, neutrophils, monocytes and macrophages leading to potent induction of antibody-dependent effector mechanisms such as ADCC. Lefitolimod is a double-stranded DNA molecule without extraneous modifications. To complement the general immunological effects of lefitolimod, we will utilize two highly potent bNAbs, 3BNC117 which targets the CD4-binding site on HIV envelope, and 10-1074 which binds to the N332 glycan-site on the V3 loop of the virus envelope protein. Similarly to monotherapy with antiretrovirals, resistance often develops during treatment with a single bNAb which emphasizes that combination bNAb treatment offer broader coverage and may prevent or delay resistance development.

In summary, we are combining lefitolimod with bNAbs in order to activate and kill virus-producing cells, but in addition to these immediate effects on latently infected cells it is also conceivable that bNAbs in combination with lefitolimod will boost pre-existing and/or prime *de novo* HIV specific T and B cell responses. Thus, we hypothesize that co-administration of lefitolimod and bNAbs will (1) induce expression of HIV envelope in infected CD4+ T cells leading to their elimination through bNAb-binding and potentiated antibody-dependent effector mechanisms such as ADCC; and (2) elicit *de novo* humoral and

cellular immune responses against HIV that will lead to enhanced immunological control in the absence of ART.

18.3 Objectives

18.3.1 Primary Objective

- To evaluate the effects of TLR9 agonism (lefitolimod) and/or administration of potent bNAbs (3BNC117 and 10-1074) on virological control of HIV during analytical treatment interruption

18.3.2 Secondary Objective

- To evaluate the safety and tolerability of the Investigational Medicinal Products (IMP)s
- To compare viral load (plasma HIV-1 RNA) kinetics between the different study arms
- To compare time without ART between study arms

18.3.3 Exploratory Objective

- To evaluate the effect of the IMPs on the amount of HIV-1 DNA in persisting in CD4+ T cells
- To evaluate the effect of the IMPs on the functional HIV-1 reservoir in CD4+ T cells
- To compare HIV-specific immunity, T cell phenotype, immune activation, and cytokine production between the investigational treatment regimens

18.4 Endpoints

18.4.1 Primary Endpoint

- Time from day of cART cessation until loss of virological control defined as day on which plasma HIV-1 RNA levels have been sustained ≧1,000 copies/mL for ≧ 4 consecutive weeks or day of confirmed plasma HIV RNA >100,000 copies/mL.

18.4.2 Secondary Endpoints

- Safety evaluation, as measured by AEs, Adverse Reactions (ARs), SAEs, Serious ARs (SARs) and (SUSAR)
- Plasma HIV-1 RNA, as measured by a routine clinical assay (Cobas TaqMan; Lower limit of quantitation 20 copies/mL)
- Time to first plasma HIV-1 RNA >50 copies/mL and first plasma HIV-1 RNA >1,000 during the analytical treatment interruption

18.4.3 Potential Exploratory Analyses

- Total HIV-1 DNA and multiple-spliced HIV-1 RNA in total CD4+ T cells.
- HIV-specific T-cells will be investigated by ICS.
- Plasma cytokine and immune activation biomarker levels
- Genetic, virological, and immunological predictors of treatment response
- Plasma 3BNC117 and 10-1074 concentrations
- Phylogenetic “foot-prints” of the investigational drugs as evidenced by the genetic make-up of viral reservoir, inducible virus and rebound virus during analytical treatment interruption.
- Size of the replication competent HIV-1 reservoir as determined by the number of infectious unit per million total CD4+ T cells using a viral outgrowth assay at baseline and prior to analytical treatment interruption.

19 LIST OF ABBREVIATIONS

10-1074	Anti-HIV-1 bNAb targeting the V3 glycan supersite on HIV-1 envelope
3BNC117	Anti-HIV-1 bNAb targeting the CD4-binding site of HIV-1 envelope glycoprotein 120

Ab	Antibody
ADCC	Antibody-dependent cell-mediated cytotoxicity
AE	Adverse Event
AIDS	Acquired Immune Deficiency Syndrome
ALT	Alanine aminotransferase
(c)ART	(combination) Antiretroviral Therapy
AR	Adverse Reactions
AST	Aspartate transaminase
ATI	Analytical treatment interruption
AUC	Area under curve
AUH	Aarhus University Hospital
bNAb	Broadly neutralizing Ab
CD	Cluster of differentiation
Cmax	The maximum plasma concentration
CpG-ODN	CpG oligodeoxynucleotides
CRF	Case report form
CTCAE	Common Terminology Criteria for AEs
CTL	Cytotoxic T lymphocyte
DC	Dendritic cell
DNA	Deoxyribonucleic acid
dSLIM	Double stem loop Immunomodulator
DSUR	Development Safety Update Report
ECG	Electrocardiogram
eGFR	Estimated glomerular filtration rate
FACS	Fluorescence-activated cell sorting
FcγR	FC gamma receptor
GCP	Good Clinical Practice
HIV	Human immunodeficiency virus
HR	Heart rate
ICS	Intracellular cytokine staining
IL	Interleukin
IFN	Interferon
IMP	Investigational Medicinal Product
INR	The international normalized ratio
IP	Interferon gamma-induced protein
LLN	Lower limit of normal
mAb	Monoclonal Ab
NK	Natural killer cells
PBMCs	Peripheral blood mononuclear cells
PCR	Polymerase chain reaction
pDC	Plasmacytoid dendritic cell
PK	Pharmacokinetics
PTT	The post-partial thromboplastin time
PR	P-R interval
PSRT	Protocol Safety Review Team
PT	The prothrombin time
QRS	QRS complex
QTc	QT-interval correction
RNA	Ribonucleic acid
RR	QT/Heart Rate
SAE	Serious AE
SAR	Serious AR
SD	Standard deviation
SEM	Standard error of mean
SHIV	Simian immunodeficiency virus/HIV chimeric virus
SMC	Safety Monitoring Committee

SUSAR	Suspected Unexpected Serious Adverse Reaction
Tmax	Time of Cmax
$T_{1/2}$	Half-life
TLR	Toll-like receptor
TNF	Tumor necrosis factor
ULN	Upper limit of normal

20 LIST OF FIGURES AND TABLES

Label	Page	Title
Figure 1	14	Potential dual role for lefitolimod in “shock and kill” HIV eradication strategies.
Figure 2	15	Monotherapy with 30 mg/kg of either 3BNC117 or 10-1074 causes a similarly potent reduction in HIV viremia.
Figure 3	15	Structure of lefitolimod.
Table 1	21	Summary of animal pharmacokinetic data for lefitolimod.
Figure 4	22	Table and figure summarizing the serum PK data from the 13 healthy participants in the Mologen cross-over study MGN1703-C04.
Figure 5	23	3BNC117 decay measured in TZM-bl assays (solid lines) and ELISA (dotted lines).
Figure 6	24	10-1074 decay measured in TZM-bl assays (solid lines) and ELISA (dotted lines).
Table 2	29	Summary of all related AE’s reported for 3BNC117
Table 3	30	Summary of all related AE’s reported for 10-1074
Figure 7	31	3BNC117 and 10-1074 decay measured in TZM-bl assays and ELISA
Figure 8	33	The four TITAN study arms.
Figure 9	35	Size of the HIV-1 reservoir according to results of 3BNC117 and 10-1074 sensitivity on the PhenoSense HIVmAb Assay and/or HIV env sequencing.
Table 4	47	Overview of study visits, follow-up and safety monitoring
Figure 10	55	Work flow when reporting SAE and SUSAR.

21 REFERENCES

1. Vibholm L, Schleimann MH, Hojen JF, Benfield T, Offersen R, Rasmussen K, et al. Short-course TLR9 Agonist Treatment Impacts Innate Immunity and Plasma Viremia in Individuals with HIV infection. Clin Infect Dis. 2017:doi: 10.1093/cid/cix201.

2. El-Sadr WM, Lundgren JD, Neaton JD, Gordin F, Abrams D, Arduino RC, et al. CD4+ count-guided interruption of antiretroviral treatment. N Engl J Med. 2006;355(22):2283-96.

3. Chomont N, El-Far M, Ancuta P, Trautmann L, Procopio FA, Yassine-Diab B, et al. HIV reservoir size and persistence are driven by T cell survival and homeostatic proliferation. Nat Med. 2009;15(8):893-900.

4. Finzi D, Hermankova M, Pierson T, Carruth LM, Buck C, Chaisson RE, et al. Identification of a reservoir for HIV-1 in patients on highly active antiretroviral therapy. Science. 1997;278(5341):1295-300.

5. Finzi D, Blankson J, Siliciano JD, Margolick JB, Chadwick K, Pierson T, et al. Latent infection of CD4+ T cells provides a mechanism for lifelong persistence of HIV-1, even in patients on effective combination therapy. Nat Med. 1999;5(5):512-7.

6. Archin NM, Espeseth A, Parker D, Cheema M, Hazuda D, Margolis DM. Expression of latent HIV induced by the potent HDAC inhibitor suberoylanilide hydroxamic acid. AIDS Res Hum Retroviruses. 2009;25(2):207-12.

7. Bosque A, Planelles V. Induction of HIV-1 latency and reactivation in primary memory CD4+ T cells. Blood. 2009;113(1):58-65.

8. Henrich TJ, Hanhauser E, Marty FM, Sirignano MN, Keating S, Lee TH, et al. Antiretroviral-free HIV-1 remission and viral rebound after allogeneic stem cell transplantation: report of 2 cases. Ann Intern Med. 2014;161(5):319-27.

9. Li JZ, Etemad B, Ahmed H, Aga E, Bosch RJ, Mellors JW, et al. The size of the expressed HIV reservoir predicts timing of viral rebound after treatment interruption. AIDS (London, England). 2016;30(3):343-53.

10. Deeks SG. HIV: Shock and kill. Nature. 2012;487(7408):439-40.

11. Leth S, Schleimann MH, Nissen SK, Højen JF, Olesen R, Graversen ME, et al. The combined effect of Vacc-4x/rhuGM-CSF vaccination and romidepsin on the HIV-1 reservoir: a phase Ib/IIa, single group, clinical trial. Lancet HIV. 2016:online July 7, 2016.

12. Elliott JH, McMahon JH, Chang CC, Lee SA, Hartogensis W, Bumpus N, et al. Short-term administration of disulfiram for reversal of latent HIV infection: a phase 2 dose-escalation study. Lancet HIV. 2015;2(12):e520-9.

13. Elliott JH, Wightman F, Solomon A, Ghneim K, Ahlers J, Cameron MJ, et al. Activation of HIV Transcription with Short-Course Vorinostat in HIV-Infected Patients on Suppressive Antiretroviral Therapy. PLoS pathogens. 2014;10(10):e1004473.

14. Archin NM, Bateson R, Tripathy MK, Crooks AM, Yang KH, Dahl NP, et al. HIV-1 expression within resting CD4+ T cells after multiple doses of vorinostat. J Infect Dis. 2014;210(5):728-35.

15. Archin NM, Liberty AL, Kashuba AD, Choudhary SK, Kuruc JD, Crooks AM, et al. Administration of vorinostat disrupts HIV-1 latency in patients on antiretroviral therapy. Nature. 2012;487(7408):482-5.

16. Rasmussen TA, Tolstrup M, Brinkmann CR, Olesen R, Erikstrup C, Solomon A, et al. Panobinostat, a histone deacetylase inhibitor, for latent-virus reactivation in HIV-infected patients on suppressive antiretroviral therapy: a phase 1/2, single group, clinical trial. Lancet HIV. 2014;1(1):e13-21.

17. Sogaard OS, Graversen ME, Leth S, Olesen R, Brinkmann CR, Nissen SK, et al. The Depsipeptide Romidepsin Reverses HIV-1 Latency In Vivo. PLoS pathogens. 2015;11(9):e1005142.

18. Shan L, Deng K, Shroff NS, Durand CM, Rabi SA, Yang HC, et al. Stimulation of HIV-1-specific cytolytic T lymphocytes facilitates elimination of latent viral reservoir after virus reactivation. Immunity. 2012;36(3):491-501.

19. Buzon MJ, Yang Y, Ouyang Z, Sun H, Seiss K, Rogich J, et al. Susceptibility to CD8 T-cell-mediated killing influences the reservoir of latently HIV-1-infected CD4 T cells. Journal of acquired immune deficiency syndromes. 2014;65(1):1-9.

20. Carrington M, Alter G. Innate immune control of HIV. Cold Spring Harb Perspect Med. 2012;2(7):a007070.

21. Deng K, Pertea M, Rongvaux A, Wang L, Durand CM, Ghiaur G, et al. Broad CTL response is required to clear latent HIV-1 due to dominance of escape mutations. Nature. 2015;517(7534):381-5.

22. Vieillard V, Fausther-Bovendo H, Samri A, Debre P, French Asymptomatiques a Long Terme A-COSG. Specific phenotypic and functional features of natural killer cells from HIV-infected long-term nonprogressors and HIV controllers. Journal of acquired immune deficiency syndromes. 2010;53(5):564-73.

23. Lu CL, Murakowski DK, Bournazos S, Schoofs T, Sarkar D, Halper-Stromberg A, et al. Enhanced clearance of HIV-1-infected cells by broadly neutralizing antibodies against HIV-1 in vivo. Science. 2016;352(6288):1001-4.

24. Hill AL, Rosenbloom DI, Fu F, Nowak MA, Siliciano RF. Predicting the outcomes of treatment to eradicate the latent reservoir for HIV-1. Proceedings of the National Academy of Sciences of the United States of America. 2014;111(37):13475-80.

25. Hill AL, Rosenbloom DI, Goldstein E, Hanhauser E, Kuritzkes DR, Siliciano RF, et al. Real-Time Predictions of Reservoir Size and Rebound Time during Antiretroviral Therapy Interruption Trials for HIV. PLoS pathogens. 2016;12(4):e1005535.

26. Luzuriaga K, Gay H, Ziemniak C, Sanborn KB, Somasundaran M, Rainwater-Lovett K, et al. Viremic relapse after HIV-1 remission in a perinatally infected child. N Engl J Med. 2015;372(8):786-8.

27. Kohrt HE, Houot R, Marabelle A, Cho HJ, Osman K, Goldstein M, et al. Combination strategies to enhance antitumor ADCC. Immunotherapy. 2012;4(5):511-27.

28. Kapp K, Kleuss C, Schroff M, Wittig B. Genuine Immunomodulation With dSLIM. Molecular therapy Nucleic acids. 2014;3:e170.

29. Damiano V, Caputo R, Garofalo S, Bianco R, Rosa R, Merola G, et al. TLR9 agonist acts by different mechanisms synergizing with bevacizumab in sensitive and cetuximab-resistant colon cancer xenografts. Proceedings of the National Academy of Sciences of the United States of America. 2007;104(30):12468-73.

30. Wittig B, Schmidt M, Scheithauer W, Schmoll HJ. MGN1703, an immunomodulator and Toll-like receptor-9 agonist: from bench to bedside. Crit Rev Oncol/Hematol. 2015;94(1):31-44.

31. Heikenwalder M, Polymenidou M, Junt T, Sigurdson C, Wagner H, Akira S, et al. Lymphoid follicle destruction and immunosuppression after repeated CpG oligodeoxynucleotide administration. Nat Med. 2004;10(2):187-92.

32. Schmidt M, Hagner N, Marco A, Konig-Merediz SA, Schroff M, Wittig B. Design and Structural Requirements of the Potent and Safe TLR-9 Agonistic Immunomodulator MGN1703. Nucleic Acid Ther. 2015;25(3):130-40.

33. Offersen R, Nissen SK, Rasmussen T, Ostergaard L, Denton PW, Sogaard OS, et al. A novel Toll-like receptor-9 agonist, MGN1703, enhances HIV-1 transcription and NK cell-mediated inhibition of HIV-1 infected autologous CD4+ T-cells. Journal of virology. 2016;90(9):4441-53.

34. Caskey M, Klein F, Lorenzi JC, Seaman MS, West AP, Jr., Buckley N, et al. Viraemia suppressed in HIV-1-infected humans by broadly neutralizing antibody 3BNC117. Nature. 2015;522(7557):487-91.

35. Scheid JF, Horwitz JA, Bar-On Y, Kreider EF, Lu CL, Lorenzi JC, et al. HIV-1 antibody 3BNC117 suppresses viral rebound in humans during treatment interruption. Nature. 2016;535(7613):556-60.

36. Caskey M, Schoofs T, Gruell H, Settler A, Karagounis T, Kreider EF, et al. Antibody 10-1074 suppresses viremia in HIV-1-infected individuals. Nat Med. 2017;23(2):185-91.

37. Halper-Stromberg A, Lu CL, Klein F, Horwitz JA, Bournazos S, Nogueira L, et al. Broadly neutralizing antibodies and viral inducers decrease rebound from HIV-1 latent reservoirs in humanized mice. Cell. 2014;158(5):989-99.

38. Klein F, Nogueira L, Nishimura Y, Phad G, West AP, Jr., Halper-Stromberg A, et al. Enhanced HIV-1 immunotherapy by commonly arising antibodies that target virus escape variants. The Journal of experimental medicine. 2014;211(12):2361-72.

39. Barouch DH, Whitney JB, Moldt B, Klein F, Oliveira TY, Liu J, et al. Therapeutic efficacy of potent neutralizing HIV-1-specific monoclonal antibodies in SHIV-infected rhesus monkeys. Nature. 2013;503(7475):224-8.

40. Bournazos S, Klein F, Pietzsch J, Seaman MS, Nussenzweig MC, Ravetch JV. Broadly neutralizing anti-HIV-1 antibodies require Fc effector functions for in vivo activity. Cell. 2014;158(6):1243-53.

41. Horwitz JA, Halper-Stromberg A, Mouquet H, Gitlin AD, Tretiakova A, Eisenreich TR, et al. HIV-1 suppression and durable control by combining single broadly neutralizing antibodies and antiretroviral drugs in humanized mice. Proceedings of the National Academy of Sciences of the United States of America. 2013;110(41):16538-43.

42. Shingai M, Donau OK, Plishka RJ, Buckler-White A, Mascola JR, Nabel GJ, et al. Passive transfer of modest titers of potent and broadly neutralizing anti-HIV monoclonal antibodies block SHIV infection in macaques. The Journal of experimental medicine. 2014;211(10):2061-74.

43. Shingai M, Nishimura Y, Klein F, Mouquet H, Donau OK, Plishka R, et al. Antibody-mediated immunotherapy of macaques chronically infected with SHIV suppresses viraemia. Nature. 2013;503(7475):277-80.

44. Schoofs T, Klein F, Braunschweig M, Kreider EF, Feldmann A, Nogueira L, et al. HIV-1 therapy with monoclonal antibody 3BNC117 elicits host immune responses against HIV-1. Science. 2016;352(6288):997-1001.

45. Weihrauch MR, Richly H, von Bergwelt-Baildon MS, Becker HJ, Schmidt M, Hacker UT, et al. Phase I clinical study of the toll-like receptor 9 agonist MGN1703 in patients with metastatic solid tumours. European journal of cancer. 2015;51(2):146-56.

46. Simek MD, Rida W, Priddy FH, Pung P, Carrow E, Laufer DS, et al. Human immunodeficiency virus type 1 elite neutralizers: individuals with broad and potent neutralizing activity identified by using a high-throughput neutralization assay together with an analytical selection algorithm. Journal of virology. 2009;83(14):7337-48.

47. Stamatatos L, Morris L, Burton DR, Mascola JR. Neutralizing antibodies generated during natural HIV-1 infection: good news for an HIV-1 vaccine? Nat Med. 2009;15(8):866-70.

48. Sather DN, Armann J, Ching LK, Mavrantoni A, Sellhorn G, Caldwell Z, et al. Factors associated with the development of cross-reactive neutralizing antibodies during human immunodeficiency virus type 1 infection. Journal of virology. 2009;83(2):757-69.

49. Li Y, Migueles SA, Welcher B, Svehla K, Phogat A, Louder MK, et al. Broad HIV-1 neutralization mediated by CD4-binding site antibodies. Nat Med. 2007;13(9):1032-4.

50. Doria-Rose NA, Klein RM, Daniels MG, O'Dell S, Nason M, Lapedes A, et al. Breadth of human immunodeficiency virus-specific neutralizing activity in sera: clustering analysis and association with clinical variables. Journal of virology. 2010;84(3):1631-6.

51. Trkola A, Kuster H, Rusert P, Joos B, Fischer M, Leemann C, et al. Delay of HIV-1 rebound after cessation of antiretroviral therapy through passive transfer of human neutralizing antibodies. Nat Med. 2005;11(6):615-22.

52. Mehandru S, Vcelar B, Wrin T, Stiegler G, Joos B, Mohri H, et al. Adjunctive passive immunotherapy in human immunodeficiency virus type 1-infected individuals treated with antiviral therapy during acute and early infection. Journal of virology. 2007;81(20):11016-31.

53. Scheid JF, Mouquet H, Ueberheide B, Diskin R, Klein F, Oliveira TY, et al. Sequence and structural convergence of broad and potent HIV antibodies that mimic CD4 binding. Science. 2011;333(6049):1633-7.

54. Wardemann H, Yurasov S, Schaefer A, Young JW, Meffre E, Nussenzweig MC. Predominant autoantibody production by early human B cell precursors. Science. 2003;301(5638):1374-7.

55. Tiller T, Meffre E, Yurasov S, Tsuiji M, Nussenzweig MC, Wardemann H. Efficient generation of monoclonal antibodies from single human B cells by single cell RT-PCR and expression vector cloning. Journal of immunological methods. 2008;329(1-2):112-24.

56. Scheid JF, Mouquet H, Feldhahn N, Walker BD, Pereyra F, Cutrell E, et al. A method for identification of HIV gp140 binding memory B cells in human blood. Journal of immunological methods. 2009;343(2):65-7.

57. Mouquet H, Scharf L, Euler Z, Liu Y, Eden C, Scheid JF, et al. Complex-type N-glycan recognition by potent broadly neutralizing HIV antibodies. Proceedings of the National Academy of Sciences of the United States of America. 2012;109(47):E3268-77.

58. Nishimura Y, Gautam R, Chun TW, Sadjadpour R, Foulds KE, Shingai M, et al. Early antibody therapy can induce long-lasting immunity to SHIV. Nature. 2017;543(7646):559-63.

59. Gautam R, Nishimura Y, Pegu A, Nason MC, Klein F, Gazumyan A, et al. A single injection of anti-HIV-1 antibodies protects against repeated SHIV challenges. Nature. 2016;533(7601):105-9.

60. Krarup AR, Abdel-Mohsen M, Schleimann MH, Vibholm L, Engen PA, Dige A, et al. The TLR9 agonist MGN1703 triggers a potent type I interferon response in the sigmoid colon. Mucosal immunology. 2017;11(2):449-61.

61. Vidarsson G, Dekkers G, Rispens T. IgG subclasses and allotypes: from structure to effector functions. Frontiers in immunology. 2014;5:520.

62. Zhou H, Mascelli MA. Mechanisms of monoclonal antibody-drug interactions. Annual review of pharmacology and toxicology. 2011;51:359-72.

63. Hemming VG. Use of intravenous immunoglobulins for prophylaxis or treatment of infectious diseases. Clinical and diagnostic laboratory immunology. 2001;8(5):859-63.

64. Onyango-Makumbi C, Omer SB, Mubiru M, Moulton LH, Nakabiito C, Musoke P, et al. Safety and efficacy of HIV hyperimmune globulin for prevention of mother-to-child HIV transmission in HIV-1-infected pregnant women and their infants in Kampala, Uganda (HIVIGLOB/NVP STUDY). Journal of acquired immune deficiency syndromes. 2011;58(4):399-407.

65. Cavacini LA, Samore MH, Gambertoglio J, Jackson B, Duval M, Wisnewski A, et al. Phase I study of a human monoclonal antibody directed against the CD4-binding site of HIV type 1 glycoprotein 120. AIDS Res Hum Retroviruses. 1998;14(7):545-50.

66. Armbruster C, Stiegler GM, Vcelar BA, Jager W, Michael NL, Vetter N, et al. A phase I trial with two human monoclonal antibodies (hMAb 2F5, 2G12) against HIV-1. AIDS (London, England). 2002;16(2):227-33.

67. Armbruster C, Stiegler GM, Vcelar BA, Jager W, Koller U, Jilch R, et al. Passive immunization with the anti-HIV-1 human monoclonal antibody (hMAb) 4E10 and the hMAb combination 4E10/2F5/2G12. The Journal of antimicrobial chemotherapy. 2004;54(5):915-20.

68. Vibholm LK, Frattari G, Schleimann MH, Olesen R, Lichterfeld M, Winckelmann A, et al., editors. Effect of 24 Weeks TLR9 Agonist Therapy on CTL Responses and Viral Rebound During ATI. CROI conference, Poster ID 357; 2018.

69. West AP, Jr., Scharf L, Horwitz J, Klein F, Nussenzweig MC, Bjorkman PJ. Computational analysis of anti-HIV-1 antibody neutralization panel data to identify potential functional epitope residues. Proc Natl Acad Sci U S A. 2013;110(26):10598-603.

70. Krieg AM, Efler SM, Wittpoth M, Al Adhami MJ, Davis HL. Induction of systemic TH1-like innate immunity in normal volunteers following subcutaneous but not intravenous administration of CPG 7909, a synthetic B-class CpG oligodeoxynucleotide TLR9 agonist. J Immunother. 2004;27(6):460-71.

71. Brussel A, Sonigo P. Analysis of early human immunodeficiency virus type 1 DNA synthesis by use of a new sensitive assay for quantifying integrated provirus. Journal of virology. 2003;77(18):10119-24.

72. Leth S, Nymann R, S JO, Olesen R, Rasmussen TA, Ostergaard L, et al. HIV-1 transcriptional activity during frequent longitudinal sampling in aviremic patients on ART. AIDS (London, England). 2016;30(5):713-21.

73. Lamoreaux L, Roederer M, Koup R. Intracellular cytokine optimization and standard operating procedure. Nature protocols. 2006;1(3):1507-16.

74. Deeks SG, Phillips AN. HIV infection, antiretroviral treatment, ageing, and non-AIDS related morbidity. BMJ. 2009;338:a3172.

75. Sogaard OS, Lohse N, Gerstoft J, Kronborg G, Ostergaard L, Pedersen C, et al. Mortality after hospitalization for pneumonia among individuals with HIV, 1995-2008: a Danish cohort study. PLoS One. 2009;4(9):e7022.

76. El-Sadr WM, Lundgren J, Neaton JD, Gordin F, Abrams D, Arduino RC, et al. CD4+ count-guided interruption of antiretroviral treatment. N Engl J Med. 2006;355(22):2283-96.

77. Kutzler MA, Jacobson JM. Treatment interruption as a tool to measure changes in immunologic response to HIV-1. Current opinion in HIV and AIDS. 2008;3(2):131-5.

78. Paton NI. Treatment interruption strategies: how great are the risks? Current opinion in infectious diseases. 2008;21(1):25-30.

79. Papasavvas E, Grant RM, Sun J, Mackiewicz A, Pistilli M, Gallo C, et al. Lack of persistent drug-resistant mutations evaluated within and between treatment interruptions in chronically HIV-1-infected patients. AIDS (London, England). 2003;17(16):2337-43.

80. Azzoni L, Papasavvas E, Montaner LJ. Lessons learned from HIV treatment interruption: safety, correlates of immune control, and drug sparing. Current HIV research. 2003;1(3):329-42.
