# Supplementary material for: Impact of a TLR9 agonist and broadly neutralizing antibodies on HIV-1 persistence: the randomized phase 2a TITAN trial
Source: Nat Med. 2023 Sep 11;29(10):2547–58. doi: 10.1038/s41591-023-02547-6 (PMC10579101; doi:10.1038/s41591-023-02547-6)
Supplement: Supplementary file 2 — Reporting Summary [file 41591_2023_2547_MOESM2_ESM.pdf]

nature portfolio

Corresponding author(s): Ole Schmeltz Søgaard

Last updated by author(s): Aug 9, 2023

Reporting Summary

Nature Portfolio wishes to improve the reproducibility of the work that we publish. This form provides structure for consistency and transparency in reporting. For further information on Nature Portfolio policies, see our Editorial Policies and the Editorial Policy Checklist.

Statistics

For all statistical analyses, confirm that the following items are present in the figure legend, table legend, main text, or Methods section.

n/a	Confirmed	
☐	☒	The exact sample size (*n*) for each experimental group/condition, given as a discrete number and unit of measurement
☐	☒	A statement on whether measurements were taken from distinct samples or whether the same sample was measured repeatedly
☐	☒	The statistical test(s) used AND whether they are one- or two-sided<br>*Only common tests should be described solely by name; describe more complex techniques in the Methods section.*
☐	☒	A description of all covariates tested
☐	☒	A description of any assumptions or corrections, such as tests of normality and adjustment for multiple comparisons
☐	☒	A full description of the statistical parameters including central tendency (e.g. means) or other basic estimates (e.g. regression coefficient) AND variation (e.g. standard deviation) or associated estimates of uncertainty (e.g. confidence intervals)
☐	☒	For null hypothesis testing, the test statistic (e.g. *F*, *t*, *r*) with confidence intervals, effect sizes, degrees of freedom and *P* value noted<br>*Give P values as exact values whenever suitable.*
☒	☐	For Bayesian analysis, information on the choice of priors and Markov chain Monte Carlo settings
☒	☐	For hierarchical and complex designs, identification of the appropriate level for tests and full reporting of outcomes
☐	☒	Estimates of effect sizes (e.g. Cohen's *d*, Pearson's *r*), indicating how they were calculated

Our web collection on statistics for biologists contains articles on many of the points above.

Software and code

Policy information about availability of computer code

Data collection	Study data were collected and managed in Research Electronic Data Capture (REDCap) electronic data capture tools version 13.1.29 hosted at the Clinical Trial Unit, Department of Clinical Medicine, Aarhus University, Aarhus, Denmark.
Data analysis	We used Stata version 17.0, Prism version 7.0 for statistical analyses, and QuantaSoft software (BioRad, version 1.7.4).

For manuscripts utilizing custom algorithms or software that are central to the research but not yet described in published literature, software must be made available to editors and reviewers. We strongly encourage code deposition in a community repository (e.g. GitHub). See the Nature Portfolio guidelines for submitting code & software for further information.

Data

Policy information about availability of data

All manuscripts must include a data availability statement. This statement should provide the following information, where applicable:

- Accession codes, unique identifiers, or web links for publicly available datasets
- A description of any restrictions on data availability
- For clinical datasets or third party data, please ensure that the statement adheres to our policy

Data are not available for download due to privacy/ethical restrictions under the EU GDPR. Specific requests for access to the trial data may be sent to olesoega@rm.dk and access may be provided to a named individual in agreement with the rules and regulations (https://www.datatilsynet.dk/english/legislation) of the Danish Data Protection agency and the Danish National Center for Ethics with a 2-week response time-frame to requests.

All viral sequences have been deposited in GenBank with accession numbers OR014503 to OR015782 (www.ncbi.nlm.nih.gov/nucleotide/).

Research involving human participants, their data, or biological material

Policy information about studies with human participants or human data. See also policy information about sex, gender (identity/presentation), and sexual orientation and race, ethnicity and racism.

Reporting on sex and gender	The results were not disaggregated by sex and gender due to the nature of the design and sample size.
Reporting on race, ethnicity, or other socially relevant groupings	The results were not disaggregated by race, ethnicity or other socially relevant groupings due to the nature of the design and sample size.
Population characteristics	People living with HIV-1 aged 18-65 years on ART for at least 18 months, with plasma HIV-1 RNA <50 copies/mL for at least 15 months, and a CD4 T cell count >500 cells/mm3 at screening.
Recruitment	This was a phase 2a, investigator-initiated, randomized placebo-controlled, double-blinded international multicenter trial enrolling at six sites in Denmark, one site in Norway and one site in Australia.<br><br>Participants were recruited in the out-patient clinics or invitiated to a pre-screening meeting through invitations. Recruitment may have been bias towards socioeconomically advantaged individuals wanting to participant more than socioeconomically disadvantaged individuals, but recruitment was based among others on CD4+ T cell count as well as plasma HIV-1 RNA levels and not on socioeconomical data, which is an inherent bias in clinical trials, but here counteracted by study design.
Ethics oversight	The protocol was approved by the Danish Medicine Authorities (#2018092874) and the Norwegian Medicines Agency (#20/16305-25) as well as the National Committee on Health Research Ethics in Denmark (#1-10-72-292-18), the Regional Committee on Medical and Health Research Ethics in Norway (#184485) and the Alfred Human Research Ethics Committee in Australia (#Project 258/20).<br><br>The study was monitored by the Danish Good Clinical Practice Units (https://gcp-enhed.dk/) in Denmark and Australia and by the Section for Monitoring, Clinical Trial Unit, Olso (https://www.ous-research.no/ctu/) in Norway.

Note that full information on the approval of the study protocol must also be provided in the manuscript.

Field-specific reporting

Please select the one below that is the best fit for your research. If you are not sure, read the appropriate sections before making your selection.

☒ Life sciences ☐ Behavioural & social sciences ☐ Ecological, evolutionary & environmental sciences

For a reference copy of the document with all sections, see nature.com/documents/nr-reporting-summary-flat.pdf

Life sciences study design

All studies must disclose on these points even when the disclosure is negative.

Sample size	Of the total 46 participants enrolled in the study, 43 received the random allocation treatment.<br><br>The sample size calculation was based on the primary endpoint: time to viral rebound during ATI. Time from stopping ART to loss of virological control was compared in the four randomization groups. If loss of virological control occurred, the date of the last measurement of plasma HIV-1 RNA ≧1,000 copies/mL or confirmed >100,000 copies/mL was defined as “date of viral rebound”. Using a 2-sample comparison of means with a standard deviation (s.d.) of 11 days, 10 evaluable participants in each of the two groups would have 90% power to detect ≥16 days difference in time to viral rebound at a 5% significance level. To accommodate for dropouts, we aimed for 12 participants in each group. We considered a two-sided α value of less than 0.05 significant with no adjustments made for multiple comparisons. We used the IPDA and d3PCR assays as our primary reservoir measurement as intact proviral DNA is superior to total HIV-1 DNA in terms of estimating the intact HIV-1 reservoir6. Protocol amendments did not affect the analysis plan besides the reservoir size analyzes described above.
Data exclusions	No data were excluded.
Replication	This study was a clinical trial and the analyses were performed on individual trial participants. Experiments did not include replicates as all participants and data points are unique. All available data is included in the manuscript.
Randomization	The Clinical Trial Unit at Aarhus University generated the randomization sequence using permuted blocks of 4 or 8 by computer-generated random numbers. Randomization assignment was provided to each site through using REDCap.
Blinding	Participants, study physicians and nurses handling administrations of the study drugs as well as those doing the analyses were blinded to interventions. Only pharmacy and study personnel preparing the study drugs were unblinded to interventions.

Reporting for specific materials, systems and methods

We require information from authors about some types of materials, experimental systems and methods used in many studies. Here, indicate whether each material, system or method listed is relevant to your study. If you are not sure if a list item applies to your research, read the appropriate section before selecting a response.

Materials & experimental systems

n/a	Involved in the study
☐	☒ Antibodies
☒	☐ Eukaryotic cell lines
☒	☐ Palaeontology and archaeology
☒	☐ Animals and other organisms
☐	☒ Clinical data
☒	☐ Dual use research of concern
☒	☐ Plants

Methods

n/a	Involved in the study
☒	☐ ChIP-seq
☐	☒ Flow cytometry
☒	☐ MRI-based neuroimaging

Antibodies

Antibodies used

3BNC117 and 10-1074 are investigational anti-HIV-1 neutralizing antibody manufactured for clinical use. 3BNC117 is being investigated under US FDA INDs 118225 and 123713, respectively.

In the AIM assay, the following antibodies were used:
CD3 (PerCP/Cy5.5 anti-human CD3, SK7, BioLegend)
CD4 (BV650 anti-human CD4, RPA-T4, BioLegend)
CD8 (BV605 anti-human CD8a, RPA-T8, BioLegend)
4-1BB (PE anti-human CD137, 4B4-1, BioLegend)
CD69 (APC anti-human CD69, FN50, BioLegend)
PD-L1 (BV421 anti-human CD274, B7-H1, BioLegend)
OX40 (FITC anti-human CD134, Ber-ACT35 (ACT35), BioLegend)

Validation

3BNC117 and 10-0174 that were administered to the participants were manufactured by Celldex Therapeutics under Good Manufacturing Practice and has been fully characterized in terms of biophysical properties and potency (INDs 118225 and 123713). 3BNC117 and 10-1074 are under long-term stability monitoring.

3BNC117: Scheid, J. F. et al. Sequence and structural convergence of broad and potent HIV antibodies that mimic CD4 binding. Science 333, 1633–7 (2011).
10-1074: Mouquet, H. et al. Complex-type N-glycan recognition by potent broadly neutralizing HIV antibodies. Proc. Natl. Acad. Sci. U. S. A. 109, E3268-77 (2012).

Clinical data

Policy information about clinical studies
All manuscripts should comply with the ICMJE guidelines for publication of clinical research and a completed CONSORT checklist must be included with all submissions.

Clinical trial registration

Clinicaltrial.gov: NCT03837756, EudraCT number: 2018-001165-16.

Study protocol

TITAN-001, verison 3.0, 02 July 2021.

Data collection

Eligible individuals were recruited from 12 April 2019 to 05 November 2021, and the last follow-up visit occurred on 09 June 2022.

Study data were collected and managed in Research Electronic Data Capture (REDCap) electronic data capture tools hosted at the Clinical Trial Unit, Department of Clinical Medicine, Aarhus University, Aarhus, Denmark.

Outcomes

The primary endpoint was 1) time to loss of virological control during ATI. We defined loos of virologic control as sustained plasma HIV-1 RNA >=1000 copies/mL for 4 weeks or confirmed >100,000 copies/mL. Secondary endpoints were 1) safety including CD4+ T cell counts, and 2) viral kinetics during ATI: time to plasma HIV-1 RNA >50 and >1,000 copies/mL as well as doubling time of the initial increase in plasma HIV-1 RNA. Exploratory endpoints were 1) changes in reservoir size measured by intact HIV-1 proviruses, and 2) effects on HIV-1-specific T-cell imunity using the AIM assay.

Flow Cytometry

Plots

Confirm that:

☒ The axis labels state the marker and fluorochrome used (e.g. CD4-FITC).

☒ The axis scales are clearly visible. Include numbers along axes only for bottom left plot of group (a 'group' is an analysis of identical markers).

☒ All plots are contour plots with outliers or pseudocolor plots.

☒ A numerical value for number of cells or percentage (with statistics) is provided.

Methodology

Sample preparation	AIM: Cryopreserved PBMCs were thawed, washed and rested at 37̊C for 3 hours. Cells were then plated into wells of a 96-well plate, at a total of 1x10^6 cells per well and stimulated with the different conditions. Following 20 hours incubation at 37̊C, cells were washed with PBS and stained for viability for 20 minutes. Cells were then incubated with Human TruStain FcX in PBS 2% FBS for 10 minutes and stained 30 minutes with surface markers antibodies. Cells were washed twice and acquired.
Instrument	MACSQuant® Analyzer 16 Flow Cytometer (Miltenyi Biotec)
Software	Flow cytometry data were analyzed using FlowJo software, version 10.7.1 (Tree Star).
Cell population abundance	No sorting was performed.
Gating strategy	Live cells > single cells > lymphocytes > CD3+ cells > CD4+ and CD8+ cells. The frequency of antigen-specific cells (AIM+ cells) was determined by subtracting the frequency of the non-stimulation condition from the antigen stimulated conditions (Gag, Env, Nef and Pol). Gag-specific AIM+ cells were considered as the addition of the frequency of cells that were either CD69+PD-L1+4-1BB+OX40+, CD69+PD-L1+OX40+, 4-1BB+OX40+PD-L1+, CD69+PD-L1+4-1BB+, OX40+CD69+4-1BB+, CD69+PD-L1+, CD69+4-1BB+, OX40+PD-L1+, CD69+OX40+, 4-1BB+OX40+ or PD-L1+4-1BB+. Total HIV-specific AIM+ cells was calculated as summation of each of the 11 populations for the four antigen-stimulations.

☒ Tick this box to confirm that a figure exemplifying the gating strategy is provided in the Supplementary Information.
